# Supplementary material for: Molecular Basis of Histone Tail Recognition by Human TIP5 PHD Finger and Bromodomain of the Chromatin Remodeling Complex NoRC
Source: Structure. 2015 Jan 6;23(1):80–92. doi: 10.1016/j.str.2014.10.017 (PMC4291147; doi:10.1016/j.str.2014.10.017)
Supplement: Document S1. Supplemental Experimental Procedures, Figures S1–S6, and Table S1 [file mmc1.pdf]

Structure, Volume *23*

Supplemental Information

Molecular Basis of Histone Tail Recognition

by Human TIP5 PHD Finger and Bromodomain

# of the Chromatin Remodeling Complex NoRC

Cynthia Tallant, Erica Valentini, Oleg Fedorov, Lois Overvoorde, Fleur M. Ferguson, Panagis Filippakopoulos, Dmitri I. Svergun, Stefan Knapp, and Alessio Ciulli

A

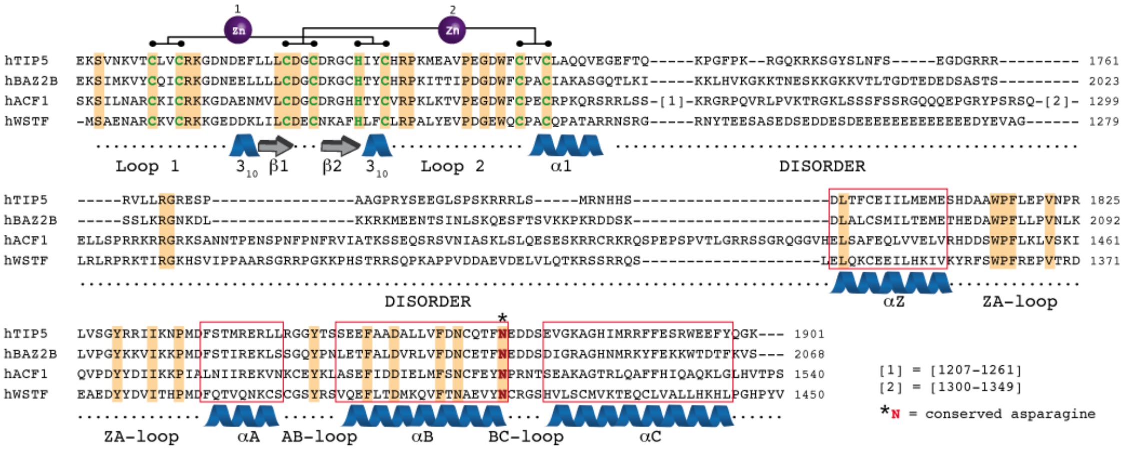

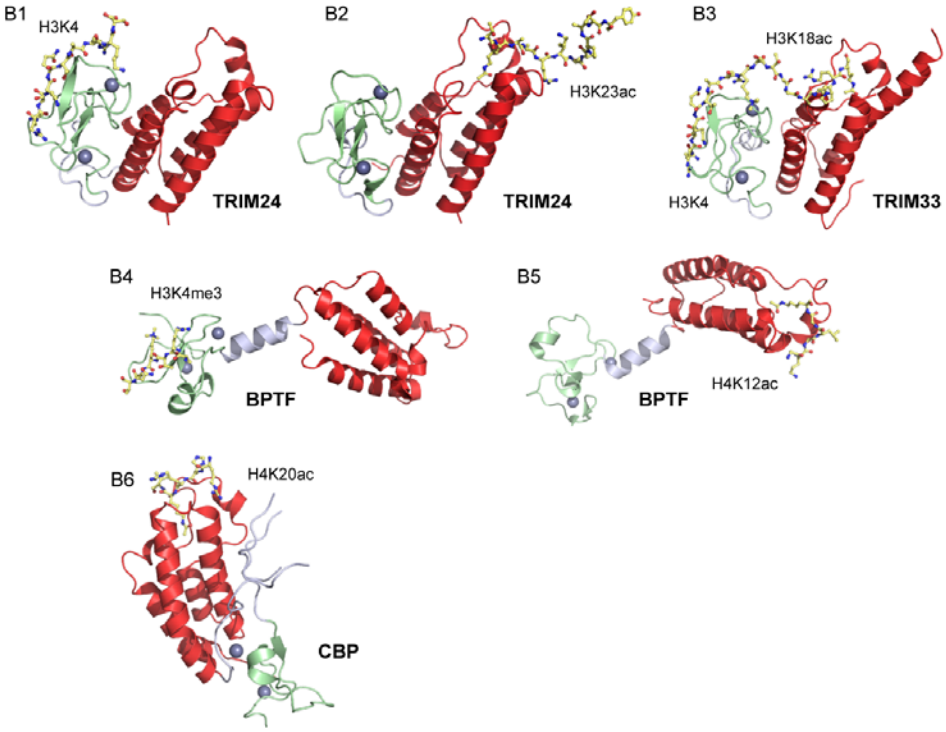

Figure S1 (related to Figure 1). A. Structure-based sequence alignment of the PHD zinc finger and bromodomain region of human chromatin remodelled proteins: hTIP5 (Q9UIF9_BAZ2A), hBAZ2B (Q9UIF8_BAZ2B), hACF1 (Q9NRL2_BAZ1A) and hWSTF (Q9UIG0_BAZ1B). **B.** Histone bound crystal structures of related proteins with PHD zinc fingers adjacent to bromodomains. **B1**. TRIM24 PHD-Bromo in complex with H3(1-10)K4 histone (3O37.pdb). **B2**. TRIM24 PHD-Bromo in complex with H3(13-32)K23ac histone (3O34.pdb). **B3**. TRIM33 PHD-Bromo in complex of single H3(1-28) K9me3K14acK18acK23ac histone (3U5P.pdb). **B4**. BPTF PHD-linker-bromodomain in complex with H3(1-15)K4me3 histone (2F6J.pdb). **B5**. BPTF PHD-linker-bromodomain in complex with H4(7-12)K12ac histone (3QZV.pdb). **B6**. CBP Bromodomain-PHD in complex with H4(13-26)K20ac histone (4N3W.pdb).

A

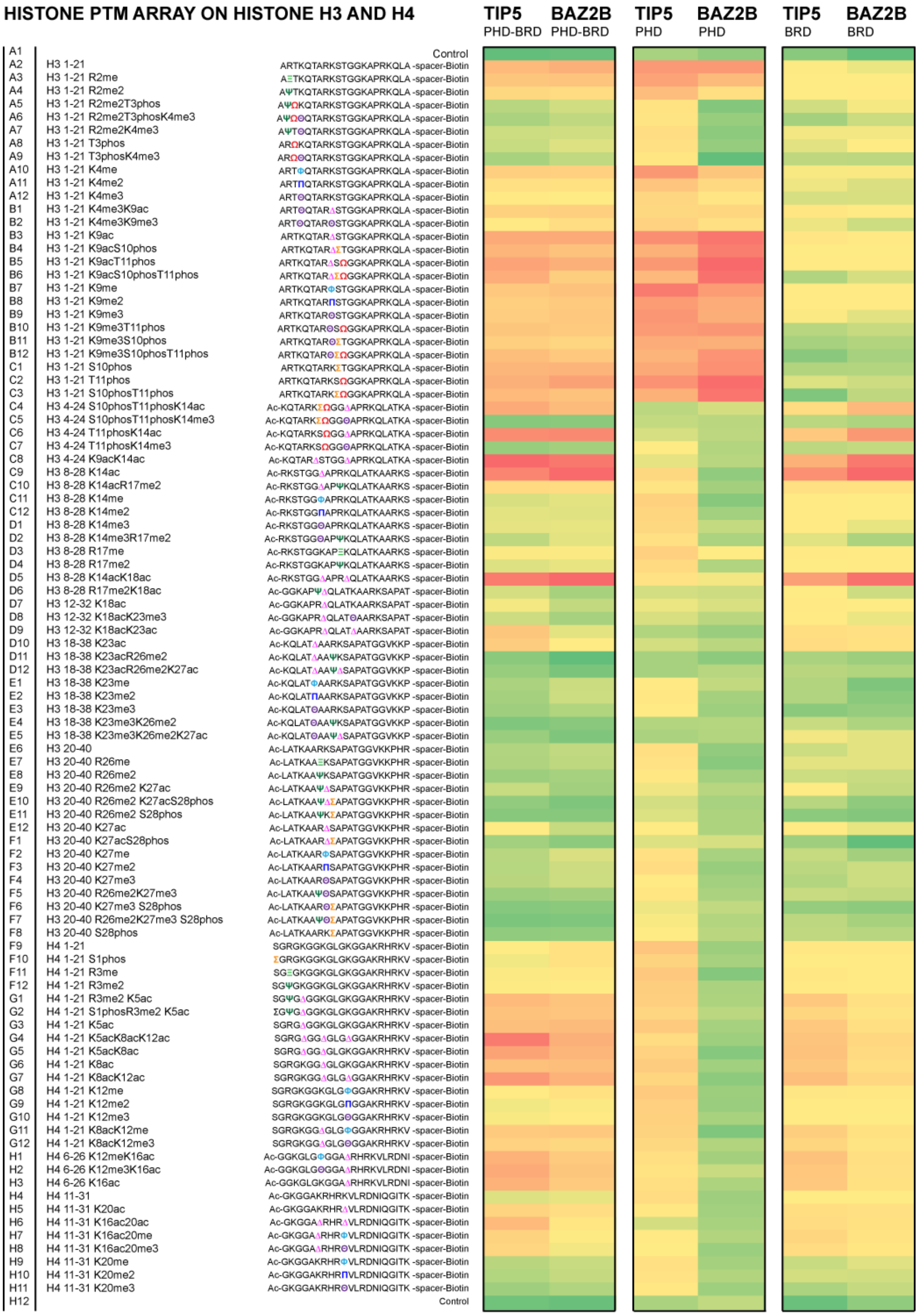
HISTONE PTM ARRAY ON HISTONE H3 AND H4
TIP5 PHD-BRD
BAZ2B PHD-BRD
TIP5 PHD
BAZ2B PHD
TIP5 BRD
BAZ2B BRD
A1 Control
A2 H3 1-21 ARTKQTARKSTGGKAPRKQLA -spacer-Biotin
A3 H3 1-21 R2me AΞTKQTARKSTGGKAPRKQLA -spacer-Biotin
A4 H3 1-21 R2me2 AΨTKQTARKSTGGKAPRKQLA -spacer-Biotin
A5 H3 1-21 R2me2T3phos AΨΩKQTARKSTGGKAPRKQLA -spacer-Biotin
A6 H3 1-21 R2me2T3phosK4me3 AΨΩΘQTARKSTGGKAPRKQLA -spacer-Biotin
A7 H3 1-21 R2me2K4me3 AΨTΘQTARKSTGGKAPRKQLA -spacer-Biotin
A8 H3 1-21 T3phos ARΩKQTARKSTGGKAPRKQLA -spacer-Biotin
A9 H3 1-21 T3phosK4me3 ARΩΘQTARKSTGGKAPRKQLA -spacer-Biotin
A10 H3 1-21 K4me ARTΦQTARKSTGGKAPRKQLA -spacer-Biotin
A11 H3 1-21 K4me2 ARTΠQTARKSTGGKAPRKQLA -spacer-Biotin
A12 H3 1-21 K4me3 ARTΘQTARKSTGGKAPRKQLA -spacer-Biotin
B1 H3 1-21 K4me3K9ac ARTΘQTARΔSTGGKAPRKQLA -spacer-Biotin
B2 H3 1-21 K4me3K9me3 ARTΘQTARΘSTGGKAPRKQLA -spacer-Biotin
B3 H3 1-21 K9ac ARTKQTARΔSTGGKAPRKQLA -spacer-Biotin
B4 H3 1-21 K9acS10phos ARTKQTARΔΣTGGKAPRKQLA -spacer-Biotin
B5 H3 1-21 K9acT11phos ARTKQTARΔSΩGGKAPRKQLA -spacer-Biotin
B6 H3 1-21 K9acS10phosT11phos ARTKQTARΔΣΩGGKAPRKQLA -spacer-Biotin
B7 H3 1-21 K9me ARTKQTARΦSTGGKAPRKQLA -spacer-Biotin
B8 H3 1-21 K9me2 ARTKQTARΠSTGGKAPRKQLA -spacer-Biotin
B9 H3 1-21 K9me3 ARTKQTARΘSTGGKAPRKQLA -spacer-Biotin
B10 H3 1-21 K9me3T11phos ARTKQTARΘSΩGGKAPRKQLA -spacer-Biotin
B11 H3 1-21 K9me3S10phos ARTKQTARΘΣTGGKAPRKQLA -spacer-Biotin
B12 H3 1-21 K9me3S10phosT11phos ARTKQTARΘΣΩGGKAPRKQLA -spacer-Biotin
C1 H3 1-21 S10phos ARTKQTARKΣTGGKAPRKQLA -spacer-Biotin
C2 H3 1-21 T11phos ARTKQTARKSΩGGKAPRKQLA -spacer-Biotin
C3 H3 1-21 S10phosT11phos ARTKQTARKΣΩGGKAPRKQLA -spacer-Biotin
C4 H3 4-24 S10phosT11phosK14ac Ac-KQTARKΣΩGGΔAPRKQLATKA -spacer-Biotin
C5 H3 4-24 S10phosT11phosK14me3 Ac-KQTARKΣΩGGΘAPRKQLATKA -spacer-Biotin
C6 H3 4-24 T11phosK14ac Ac-KQTARKSΩGGΔAPRKQLATKA -spacer-Biotin
C7 H3 4-24 T11phosK14me3 Ac-KQTARKSΩGGΘAPRKQLATKA -spacer-Biotin
C8 H3 4-24 K9acK14ac Ac-KQTARΔSTGGΔAPRKQLATKA -spacer-Biotin
C9 H3 8-28 K14ac Ac-RKSTGGΔAPRKQLATKAARKS -spacer-Biotin
C10 H3 8-28 K14acR17me2 Ac-RKSTGGΔAPΨKQLATKAARKS -spacer-Biotin
C11 H3 8-28 K14me Ac-RKSTGGΦAPRKQLATKAARKS -spacer-Biotin
C12 H3 8-28 K14me2 Ac-RKSTGGΠAPRKQLATKAARKS -spacer-Biotin
D1 H3 8-28 K14me3 Ac-RKSTGGΘAPRKQLATKAARKS -spacer-Biotin
D2 H3 8-28 K14me3R17me2 Ac-RKSTGGΘAPΨKQLATKAARKS -spacer-Biotin
D3 H3 8-28 R17me Ac-RKSTGGKAPΞKQLATKAARKS -spacer-Biotin
D4 H3 8-28 R17me2 Ac-RKSTGGKAPΨKQLATKAARKS -spacer-Biotin
D5 H3 8-28 K14acK18ac Ac-RKSTGGΔAPRΔQLATKAARKS -spacer-Biotin
D6 H3 8-28 R17me2K18ac Ac-GGKAPΨΔQLATKAARKSAPAT -spacer-Biotin
D7 H3 12-32 K18ac Ac-GGKAPRΔQLATKAARKSAPAT -spacer-Biotin
D8 H3 12-32 K18acK23me3 Ac-GGKAPRΔQLATΘAARKSAPAT -spacer-Biotin
D9 H3 12-32 K18acK23ac Ac-GGKAPRΔQLATΔAARKSAPAT -spacer-Biotin
D10 H3 18-38 K23ac Ac-KQLATΔAARKSAPATGGVKKP -spacer-Biotin
D11 H3 18-38 K23acR26me2 Ac-KQLATΔAAΨKSAPATGGVKKP -spacer-Biotin
D12 H3 18-38 K23acR26me2K27ac Ac-KQLATΔAAΨΔSAPATGGVKKP -spacer-Biotin
E1 H3 18-38 K23me Ac-KQLATΦAARKSAPATGGVKKP -spacer-Biotin
E2 H3 18-38 K23me2 Ac-KQLATΠAARKSAPATGGVKKP -spacer-Biotin
E3 H3 18-38 K23me3 Ac-KQLATΘAARKSAPATGGVKKP -spacer-Biotin
E4 H3 18-38 K23me3K26me2 Ac-KQLATΘAAΨKSAPATGGVKKP -spacer-Biotin
E5 H3 18-38 K23me3K26me2K27ac Ac-KQLATΘAAΨΔSAPATGGVKKP -spacer-Biotin
E6 H3 20-40 Ac-LATKAARKSAPATGGVKKPHR -spacer-Biotin
E7 H3 20-40 R26me Ac-LATKAAΞKSAPATGGVKKPHR -spacer-Biotin
E8 H3 20-40 R26me2 Ac-LATKAAΨKSAPATGGVKKPHR -spacer-Biotin
E9 H3 20-40 R26me2 K27ac Ac-LATKAAΨΔSAPATGGVKKPHR -spacer-Biotin
E10 H3 20-40 R26me2 K27acS28phos Ac-LATKAAΨΔΣAPATGGVKKPHR -spacer-Biotin
E11 H3 20-40 R26me2 S28phos Ac-LATKAAΨKΣAPATGGVKKPHR -spacer-Biotin
E12 H3 20-40 K27ac Ac-LATKAARΔSAPATGGVKKPHR -spacer-Biotin
F1 H3 20-40 K27acS28phos Ac-LATKAARΔΣAPATGGVKKPHR -spacer-Biotin
F2 H3 20-40 K27me Ac-LATKAARΦSAPATGGVKKPHR -spacer-Biotin
F3 H3 20-40 K27me2 Ac-LATKAARΠSAPATGGVKKPHR -spacer-Biotin
F4 H3 20-40 K27me3 Ac-LATKAARΘSAPATGGVKKPHR -spacer-Biotin
F5 H3 20-40 R26me2K27me3 Ac-LATKAAΨΘSAPATGGVKKPHR -spacer-Biotin
F6 H3 20-40 K27me3 S28phos Ac-LATKAARΘΣAPATGGVKKPHR -spacer-Biotin
F7 H3 20-40 R26me2K27me3 S28phos Ac-LATKAAΨΘΣAPATGGVKKPHR -spacer-Biotin
F8 H3 20-40 S28phos Ac-LATKAARKΣAPATGGVKKPHR -spacer-Biotin
F9 H4 1-21 SGRGKGGKGLGKGGAKRHRKV -spacer-Biotin
F10 H4 1-21 S1phos ΣGRGKGGKGLGKGGAKRHRKV -spacer-Biotin
F11 H4 1-21 R3me SGΞGKGGKGLGKGGAKRHRKV -spacer-Biotin
F12 H4 1-21 R3me2 SGΨGKGGKGLGKGGAKRHRKV -spacer-Biotin
G1 H4 1-21 R3me2 K5ac SGΨGΔGGKGLGKGGAKRHRKV -spacer-Biotin
G2 H4 1-21 S1phosR3me2 K5ac ΣGΨGΔGGKGLGKGGAKRHRKV -spacer-Biotin
G3 H4 1-21 K5ac SGRGΔGGKGLGKGGAKRHRKV -spacer-Biotin
G4 H4 1-21 K5acK8acK12ac SGRGΔGGΔGLGΔGGAKRHRKV -spacer-Biotin
G5 H4 1-21 K5acK8ac SGRGΔGGΔGLGKGGAKRHRKV -spacer-Biotin
G6 H4 1-21 K8ac SGRGKGGΔGLGKGGAKRHRKV -spacer-Biotin
G7 H4 1-21 K8acK12ac SGRGKGGΔGLGΔGGAKRHRKV -spacer-Biotin
G8 H4 1-21 K12me SGRGKGGKGLGΦGGAKRHRKV -spacer-Biotin
G9 H4 1-21 K12me2 SGRGKGGKGLGΠGGAKRHRKV -spacer-Biotin
G10 H4 1-21 K12me3 SGRGKGGKGLGΘGGAKRHRKV -spacer-Biotin
G11 H4 1-21 K8acK12me SGRGKGGΔGLGΦGGAKRHRKV -spacer-Biotin
G12 H4 1-21 K8acK12me3 SGRGKGGΔGLGΘGGAKRHRKV -spacer-Biotin
H1 H4 6-26 K12meK16ac Ac-GGKGLGΦGGAΔRHRKVLRDNI -spacer-Biotin
H2 H4 6-26 K12me3K16ac Ac-GGKGLGΘGGAΔRHRKVLRDNI -spacer-Biotin
H3 H4 6-26 K16ac Ac-GGKGLGKGGAΔRHRKVLRDNI -spacer-Biotin
H4 H4 11-31 Ac-GKGGAKRHRKVLRDNIQGITK -spacer-Biotin
H5 H4 11-31 K20ac Ac-GKGGAKRHRΔVLRDNIQGITK -spacer-Biotin
H6 H4 11-31 K16ac20ac Ac-GKGGAΔRHRΔVLRDNIQGITK -spacer-Biotin
H7 H4 11-31 K16ac20me Ac-GKGGAΔRHRΦVLRDNIQGITK -spacer-Biotin
H8 H4 11-31 K16ac20me3 Ac-GKGGAΔRHRΘVLRDNIQGITK -spacer-Biotin
H9 H4 11-31 K20me Ac-GKGGAKRHRΦVLRDNIQGITK -spacer-Biotin
H10 H4 11-31 K20me2 Ac-GKGGAKRHRΠVLRDNIQGITK -spacer-Biotin
H11 H4 11-31 K20me3 Ac-GKGGAKRHRΘVLRDNIQGITK -spacer-Biotin
H12 Control

B

HISTONE ACETYL LYSINE ARRAY ON H2A, H2B, H3, H4

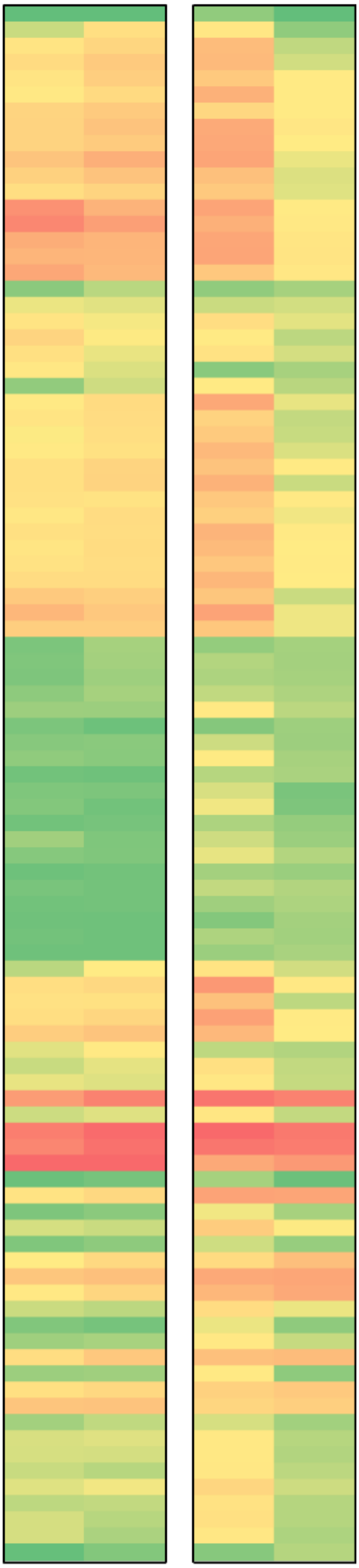

A1		Control
A2	H4 1-21	SGRGKGGKGLGKGGAKRHRKV-spacer-Biotin
A3	H4 1-21 K5ac	SGRG△GGKGLGKGGAKRHRKV-spacer-Biotin
A4	H4 1-21 K8ac	SGRGKGG△GLGKGGAKRHRKV-spacer-Biotin
A5	H4 1-21 K12ac	SGRGKGGKGLG△GGAKRHRKV-spacer-Biotin
A6	H4 1-21 K16ac	SGRGKGGKGLGKGGA△RHRKV-spacer-Biotin
A7	H4 1-21 K5acK8ac	SGRG△GG△GLGKGGAKRHRKV-spacer-Biotin
A8	H4 1-21 K5acK12ac	SGRG△GGKGLG△GGAKRHRKV-spacer-Biotin
A9	H4 1-21 K5acK16ac	SGRG△GGKGLGKGGA△RHRKV-spacer-Biotin
A10	H4 1-21 K8acK12ac	SGRGKGG△GLG△GGAKRHRKV-spacer-Biotin
A11	H4 1-21 K8acK16ac	SGRGKGG△GLGKGGA△RHRKV-spacer-Biotin
A12	H4 1-21 K12acK16ac	SGRGKGGKGLG△GGA△RHRKV-spacer-Biotin
B1	H4 1-21 K5acK8acK12ac	SGRG△GG△GLG△GGAKRHRKV-spacer-Biotin
B2	H4 1-21 K8acK12acK16ac	SGRGKGG△GLG△GGA△RHRKV-spacer-Biotin
B3	H4 1-21 K5acK12acK16ac	SGRG△GGKGLG△GGA△RHRKV-spacer-Biotin
B4	H4 1-21 K5acK8acK16ac	SGRG△GG△GLGKGGA△RHRKV-spacer-Biotin
B5	H4 1-21 K5acK8acK12acK16ac	SGRG△GG△GLG△GGA△RHRKV-spacer-Biotin
B6	H4 9-29	Ac-GLGKGGAKRHRKVLRDNIQGI-spacer-Biotin
B7	H4 9-29 K20ac	Ac-GLGKGGAKRHR△VLRDNIQGI-spacer-Biotin
B8	H4 9-29 K16ac	Ac-GLGKGGA△RHRKVLRDNIQGI-spacer-Biotin
B9	H4 9-29 K12acK16ac	Ac-GLG△GGA△RHRKVLRDNIQGI-spacer-Biotin
B10	H4 9-29 K16acK20ac	Ac-GLGKGGA△RHR△VLRDNIQGI-spacer-Biotin
B11	H4 9-29 K12acK16acK20ac	Ac-GLG△GGA△RHR△VLRDNIQGI-spacer-Biotin
B12	H2a 1-21	SGRGKQGGKARAKAKTRSSRA-spacer-Biotin
C1	H2a 1-21 K5ac	SGRG△QGGKARAKAKTRSSRA-spacer-Biotin
C2	H2a 1-21 K9ac	SGRGKQGG△ARAKAKTRSSRA-spacer-Biotin
C3	H2a 1-21 K13ac	SGRGKQGGKARA△AKTRSSRA-spacer-Biotin
C4	H2a 1-21 K15ac	SGRGKQGGKARAKA△TRSSRA-spacer-Biotin
C5	H2a 1-21 K5acK9ac	SGRG△QGG△ARAKAKTRSSRA-spacer-Biotin
C6	H2a 1-21 K5acK13ac	SGRG△QGGKARA△AKTRSSRA-spacer-Biotin
C7	H2a 1-21 K5acK15ac	SGRG△QGGKARAKA△TRSSRA-spacer-Biotin
C8	H2a 1-21 K9acK13ac	SGRGKQGG△ARA△AKTRSSRA-spacer-Biotin
C9	H2a 1-21 K9acK15ac	SGRGKQGG△ARAKA△TRSSRA-spacer-Biotin
C10	H2a 1-21 K13acK15ac	SGRGKQGGKARA△A△TRSSRA-spacer-Biotin
C11	H2a 1-21 K5acK9acK13ac	SGRG△QGG△ARA△AKTRSSRA-spacer-Biotin
C12	H2a 1-21 K5acK13acK15ac	SGRG△QGGKARA△A△TRSSRA-spacer-Biotin
D1	H2a 1-21 K9acK13acK15ac	SGRGKQGG△ARA△A△TRSSRA-spacer-Biotin
D2	H2a 1-21 K5acK9acK15ac	SGRG△QGG△ARAKA△TRSSRA-spacer-Biotin
D3	H2a 1-21 K5acK9acK13acK15ac	SGRG△QGG△ARA△A△TRSSRA-spacer-Biotin
D4	H2b 1-21	PEPAKSAPAPKKGSKKAVTKA-spacer-Biotin
D5	H2b 1-21 K5ac	PEPA△SAPAPKKGSKKAVTKA-spacer-Biotin
D6	H2b 1-21 K11ac	PEPAKSAPAP△KGSKKAVTKA-spacer-Biotin
D7	H2b 1-21 K12ac	PEPAKSAPAPK△GSKKAVTKA-spacer-Biotin
D8	H2b 1-21 K15ac	PEPAKSAPAPKKGS△KAVTKA-spacer-Biotin
D9	H2b 1-21 K5acK11acK16ac	PEPA△SAPAP△KGSK△AVTKA-spacer-Biotin
D10	H2b 1-21 K5acK12ac	PEPA△SAPAPK△GSKKAVTKA-spacer-Biotin
D11	H2b 1-21 K5acK15ac	PEPA△SAPAPKKGS△KAVTKA-spacer-Biotin
D12	H2b 1-21 K5acK16ac	PEPA△SAPAPKKGSK△AVTKA-spacer-Biotin
E1	H2b 1-21 K11acK12ac	PEPAKSAPAP△△GSKKAVTKA-spacer-Biotin
E2	H2b 1-21 K11acK15ac	PEPAKSAPAP△KGS△KAVTKA-spacer-Biotin
E3	H2b 1-21 K11acK16ac	PEPAKSAPAP△KGSK△AVTKA-spacer-Biotin
E4	H2b 1-21 K5acK11acK12ac	PEPA△SAPAP△△GSKKAVTKA-spacer-Biotin
E5	H2b 1-21 K5acK15acK16ac	PEPA△SAPAPKKGS△△AVTKA-spacer-Biotin
E6	H2b 1-21 K11acK12acK15acK16ac	PEPAKSAPAP△△GS△△AVTKA-spacer-Biotin
E7	H2b 1-21 K11acK15acK16ac	PEPAKSAPAP△KGS△△AVTKA-spacer-Biotin
E8	H2b 1-21 K12acK15acK16ac	PEPAKSAPAPK△GS△△AVTKA-spacer-Biotin
E9	H2b 1-21 K11acK12acK15ac	PEPAKSAPAP△△GS△KAVTKA-spacer-Biotin
E10	H2b 1-21 K11acK12acK16ac	PEPAKSAPAP△△GSK△AVTKA-spacer-Biotin
E11	H2b 1-21 K5acK11acK12acK15acK16ac	PEPA△SAPAP△△GS△△AVTKA-spacer-Biotin
E12	H2b 13-33	Ac-GSKKAVTKAQKKDGKKRKRSR-spacer-Biotin
F1	H2b 13-33 K20ac	Ac-GSKKAVT△AQKKDGKKRKRSR-spacer-Biotin
F2	H2b 13-33 K23ac	Ac-GSKKAVTKAQ△KDGKKRKRSR-spacer-Biotin
F3	H2b 13-33 K24ac	Ac-GSKKAVTKAQK△DGKKRKRSR-spacer-Biotin
F4	H2b 13-33 K20acK23acK24ac	Ac-GSKKAVT△AQ△△DGKKRKRSR-spacer-Biotin
F5	H3 1-21	ARTKQTARKSTGGKAPRKQLA-spacer-Biotin
F6	H3 1-21 K4ac	ART△QTARKSTGGKAPRKQLA-spacer-Biotin
F7	H3 1-21 K9ac	ARTKQTAR△STGGKAPRKQLA-spacer-Biotin
F8	H3 1-21 K14ac	ARTKQTARKSTGG△APRKQLA-spacer-Biotin
F9	H3 1-21 K4acK9ac	ART△QTAR△STGGKAPRKQLA-spacer-Biotin
F10	H3 1-21 K4acK14ac	ART△QTARKSTGG△APRKQLA-spacer-Biotin
F11	H3 1-21 K9acK14ac	ARTKQTAR△STGG△APRKQLA-spacer-Biotin
F12	H3 1-21 K4acK9acK14ac	ART△QTAR△STGG△APRKQLA-spacer-Biotin
G1	H3 11-31	Ac-TGGKAPRKQLATKAARKSAPA-spacer-Biotin
G2	H3 11-31 K14ac	Ac-TGG△APRKQLATKAARKSAPA-spacer-Biotin
G3	H3 11-31 K18ac	Ac-TGGKAPR△QLATKAARKSAPA-spacer-Biotin
G4	H3 11-31 K23ac	Ac-TGGKAPRKQLAT△AARKSAPA-spacer-Biotin
G5	H3 11-31 K27ac	Ac-TGGKAPRKQLATKAAR△SAPA-spacer-Biotin
G6	H3 11-31 K14acK18ac	Ac-TGG△APR△QLATKAARKSAPA-spacer-Biotin
G7	H3 11-31 K14acK23ac	Ac-TGG△APRKQLAT△AARKSAPA-spacer-Biotin
G8	H3 11-31 K14acK27ac	Ac-TGG△APRKQLATKAAR△SAPA-spacer-Biotin
G9	H3 11-31 K18acK23ac	Ac-TGGKAPR△QLAT△AARKSAPA-spacer-Biotin
G10	H3 11-31 K18acK27ac	Ac-TGGKAPR△QLATKAAR△SAPA-spacer-Biotin
G11	H3 11-31 K23acK27ac	Ac-TGGKAPRKQLAT△AAR△SAPA-spacer-Biotin
G12	H3 11-31 K14acK23acK27ac	Ac-TGG△APRKQLAT△AAR△SAPA-spacer-Biotin
H1	H3 11-31 K18acK23acK27ac	Ac-TGGKAPR△QLAT△AAR△SAPA-spacer-Biotin
H2	H3 11-31 K14acK18acK23ac	Ac-TGG△APR△QLAT△AARKSAPA-spacer-Biotin
H3	H3 11-31 K14acK18acK23acK27ac	Ac-TGG△APR△QLAT△AAR△SAPA-spacer-Biotin
H4	H3 23-43	Ac-KAARKSAPATGGVKKPHRYRP-spacer-Biotin
H5	H3 23-43 K36ac	Ac-KAARKSAPATGGV△KPHRYRP-spacer-Biotin
H6	H3 23-43 K37ac	Ac-KAARKSAPATGGVK△PHRYRP-spacer-Biotin
H7	H3 23-43 K36acK37ac	Ac-KAARKSAPATGGV△△PHRYRP-spacer-Biotin
H8	H3 23-43 K27ac	Ac-KAAR△SAPATGGVKKPHRYRP-spacer-Biotin
H9	H3 23-43 K27acK36ac	Ac-KAAR△SAPATGGV△KPHRYRP-spacer-Biotin
H10	H3 23-43 K27acK37ac	Ac-KAAR△SAPATGGVK△PHRYRP-spacer-Biotin
H11	H3 23-43 K27acK36acK37ac	Ac-KAAR△SAPATGGV△△PHRYRP-spacer-Biotin
H12		Control

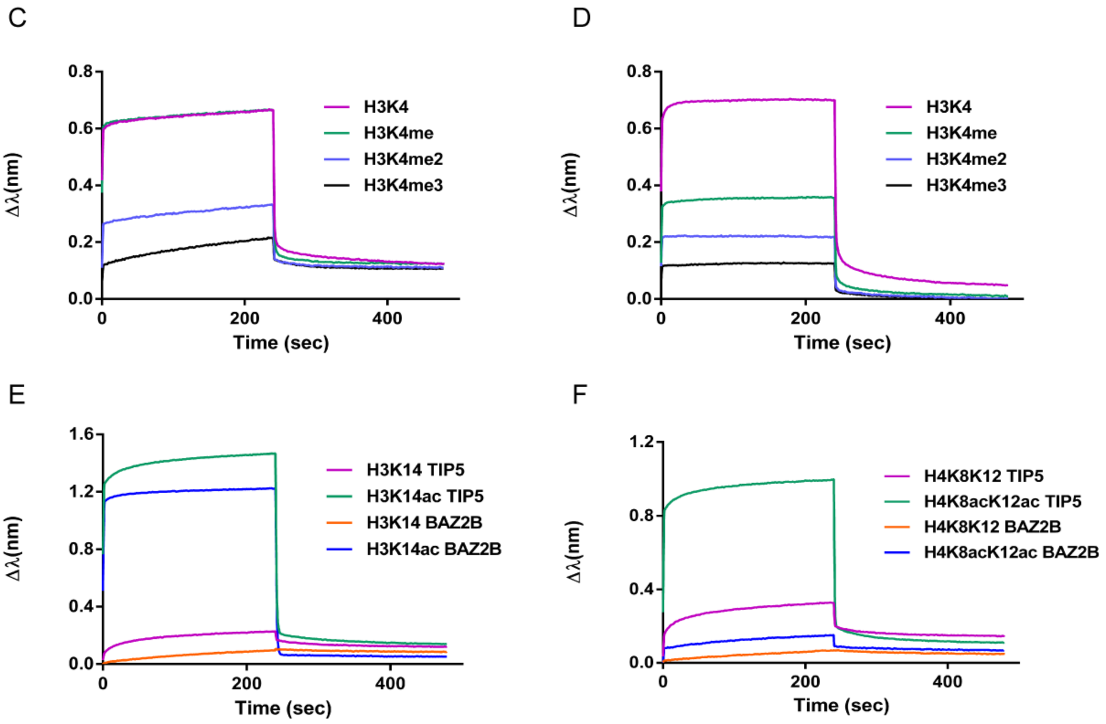

Figure S2 (related to Table 1). A. Heat map obtained from biolayer interferometry measurements against histone peptide arrays. Peptide symbol key: Δ = acetyl-Lys, ϕ = monomethyl-Lys, Π = dimethyl-Lys, Θ = trimethyl-Lys, Σ = phopho-Ser, Ω = phosphor-Thr, Ξ = monomethy-Arg, Ψ = asymdimethyl-Arg. Post-translational modification distributed to H3 and H4 histone array. **B**. Specific histone array for H2A, H2B, H3 and H4 acetylation marks. **C.** Association and dissociation curves of BLI measurements of TIP5 PHD finger binding towards unmodified/mono/di/trimethylation to H3K4. **D** BAZ2B PHD finger binding towards unmodified/mono/di/trimethylation to H3K4. **E** H3K14ac recognition for TIP5 and BAZ2B bromodomains. **F** Comparison of unmodified/acetylated of H4K8K12 for TIP5 and BAZ2B bromodomains.

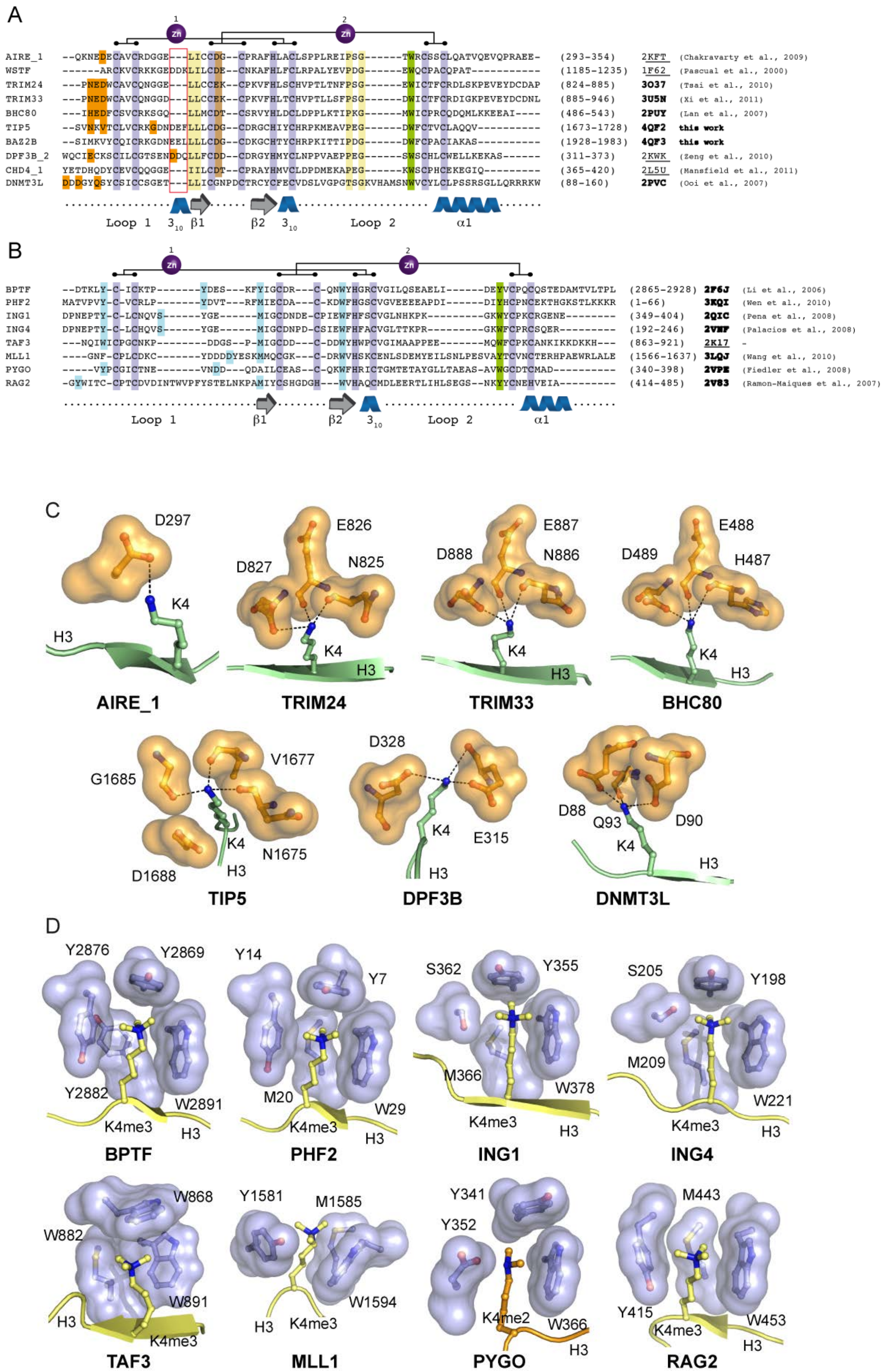
A
AIRE_1 --QKNEDECAVCRDGGE---LICCDG--CPRAFHLACLSPPLREIPSG------TWRCSSCLQATVQEVQPRAEE- (293-354) 2KFT (Chakravarty et al., 2009)
WSTF ------ARCKVCRKKGEDDKLILCDE--CNKAFHLFCLRPALYEVPDG------EWQCPACQPAT---------- (1185-1235) 1F62 (Pascual et al., 2000)
TRIM24 ---PNEDWCAVCQNGGE---LLCCEK--CPKVFHLSCHVPTLTNFPSG------EWICTFCRDLSKPEVEYDCDAP (824-885) 3O37 (Tsai et al., 2010)
TRIM33 ---PNEDWCAVCQNGGD---LLCCEK--CPKVFHLTCHVPTLLSFPSG------DWICTFCRDIGKPEVEYDCDNL (885-946) 3U5N (Xi et al., 2011)
BHC80 ---IHEDFCSVCRKSGQ---LLMCDT--CSRVYHLDCLDPPLKTIPKG------MWICPRCQDQMLKKEEAI---- (486-543) 2PUY (Lan et al., 2007)
TIP5 --SVNKVTCLVCRKGDNDEFLLLCDG--CDRGCHIYCHRPKMEAVPEG------DWFCTVCLAQQV---------- (1673-1728) 4QF2 this work
BAZ2B --SIMKVYCQICRKGDNEELLLLCDG--CDKGCHTYCHRPKITTIPDG------DWFCPACIAKAS---------- (1928-1983) 4QF3 this work
DPF3B_2 WQCIECKSCILCGTSENDDQLLFCDD--CDRGYHMYCLNPPVAEPPEG------SWSCHLCWELLKEKAS------ (311-373) 2KWK (Zeng et al., 2010)
CHD4_1 YETDHQDYCEVCQQGGE---IILCDT--CPRAYHMVCLDPDMEKAPEG------KWSCPHCEKEGIQ--------- (365-420) 2L5U (Mansfield et al., 2011)
DNMT3L DDDGYQSYCSICCSGET---LLICGNPDCTRCYCFECVDSLVGPGTSGKVHAMSNWVCYLCLPSSRSGLLQRRRKW (88-160) 2PVC (Ooi et al., 2007)
Loop 1 3₁₀ β1 β2 3₁₀ Loop 2 α1
B
BPTF --DTKLY-C-ICKTP-------YDES---KFYIGCDR---C-QNWYHGRCVGILQSEAELI------DEYVCPQCQSTEDAMTVLTPL (2865-2928) 2F6J (Li et al., 2006)
PHF2 MATVPVY-C-VCRLP-------YDVT---RFMIECDA---C-KDWFHGSCVGVEEEEAPDI------DIYHCPNCEKTHGKSTLKKKR (1-66) 3KQI (Wen et al., 2010)
ING1 DPNEPTY-C-LCNQVS------YGE------MIGCDNDE-CPIEWFHFSCVGLNHKPK---------GKWYCPKCRGENE-------- (349-404) 2QIC (Pena et al., 2008)
ING4 DPNEPTY-C-LCHQVS------YGE------MIGCDNPD-CSIEWFHFACVGLTTKPR---------GKWFCPRCSQER--------- (192-246) 2VNF (Palacios et al., 2008)
TAF3 ---NQIWICPGCNKP--------DDGS--P-MIGCDD---C-DDWYHWPCVGIMAAPPEE-------MQWFCPKCANKIKKDKKH--- (863-921) 2K17 -
MLL1 ----GNF-CPLCDKC-------YDDDDYESKMMQCGK---C-DRWVHSKCENLSDEMYEILSNLPESVAYTCVNCTERHPAEWRLALE (1566-1637) 3LQJ (Wang et al., 2010)
PYGO -----VYPCGICTNE-------VNDD---QDAILCEAS--C-QKWFHRICTGMTETAYGLLTAEAS-AVWGCDTCMAD--------- (340-398) 2VPE (Fiedler et al., 2008)
RAG2 -GYWITC-CPTCDVDINTWVPFYSTELNKPAMIYCSHGDGH---WVHAQCMDLEERTLIHLSEGS--NKYYCNEHVEIA-------- (414-485) 2V83 (Ramon-Maiques et al., 2007)
Loop 1 β1 β2 3₁₀ Loop 2 α1
C
D297
K4
H3
AIRE_1
E826
D827
N825
K4
H3
TRIM24
E887
D888
N886
K4
H3
TRIM33
E488
D489
H487
K4
H3
BHC80
V1677
G1685
K4
N1675
H3
D1688
TIP5
D328
K4
E315
H3
DPF3B
D88
Q93
D90
K4
H3
DNMT3L
D
Y2876
Y2869
Y2882
W2891
K4me3
H3
BPTF
Y14
Y7
M20
W29
K4me3
H3
PHF2
S362
Y355
M366
W378
K4me3
H3
ING1
S205
Y198
M209
W221
K4me3
H3
ING4
W868
W882
W891
H3
K4me3
TAF3
Y1581
M1585
W1594
H3
K4me3
MLL1
Y341
Y352
K4me2
W366
H3
PYGO
M443
Y415
K4me3
W453
H3
RAG2

Figure S3 (related to Figure 2). A, B Structure-based sequence alignment of histone recognition PHD zinc fingers. The domain length is indicated in brackets. Their related Protein Data Bank accession codes are also shown underlined and in bold for NMR and X-ray structures, respectively. Together, PHD fingers share secondary structure elements and have similar protein folding but they exhibit high sequence variability. The two zinc atoms are shown in purple spheres and are linked to their corresponding pairs of zinc-coordinating residues (light pink), which are highly conserved to all PHD fingers. A tryptophan or tyrosine (green) located at the end of Loop 2 is also a bulky hydrophobic conserved position pointing to the core of the PHD finger to maintain its integrity. Most of the H3K4 recognition residues are found in loop 1. **A**. Unmethylated histone recognition. The sequences are (with GenBank accession numbers): AIRE_1 (O43918 AIRE_HUMAN); WSTF (Q9UIG0 BAZ1B_HUMAN); TRIM24 (O15164 TIF1α_HUMAN); TRIM33 (Q9UPN9 TIF1γ_HUMAN); BHC80 (Q96BD5 PF21A_HUMAN); TIP5 (Q9UIF9 BAZ2A_HUMAN); BAZ2B (Q9UIF8 BAZ2B_HUMAN); DPF3B_2 (Q92784 DPF3_HUMAN); CHD4_1 (Q14839 CHD4_HUMAN); DNMT3L (Q9UJW3 DNM3L_HUMAN). The residues involved in the K4 recognition are highlighted in orange. **B**. Methylated histone recognition. Sequence alignment of methylated histone recognition PHD fingers. It includes: BPTF (Q12830 BPTF_HUMAN); PHF2 (**O75151** PHF2_HUMAN); ING1 (Q9UK53 ING1_HUMAN); ING4 (Q9UNL4 ING4_HUMAN); TAF3 (Q5VWG9 (TAF3_HUMAN); MLL1 (Q03164 KMT2A_HUMAN); PYGO (Q9Y3Y4 PYGO1_HUMAN); RAG2 (P55895 RAG2_HUMAN). The residues involved in the K4me2/me3 recognition are highlighted in light blue. A highly conserved tryptophan at the β2 strand is essential for the aromatic cage formation. **C**. Close-up view of histone recognition sites of the subclass of H3K4me0-specific PHD fingers. In most cases they possess a cluster of acidic residues and carbonyl groups from amide bonds forming hydrogen bonds and salt bridges with the side chain of unmodified K4. Methylation states of K4 decrease or supress these interactions. **D**. Close-up view of histone recognition sites of the subclass of H3K4me2/3-specific PHD fingers. These pockets form aromatic cages with different combinations of two to four aromatic residues. A tryptophan is conserved in all of them (see sequence alignment above). The residue arrangement favours the formation of cation-π, hydrophobic and van der Waals contacts with the di/trimethylammonium moiety of K4. The coordinates for all structures are listed in A, B.

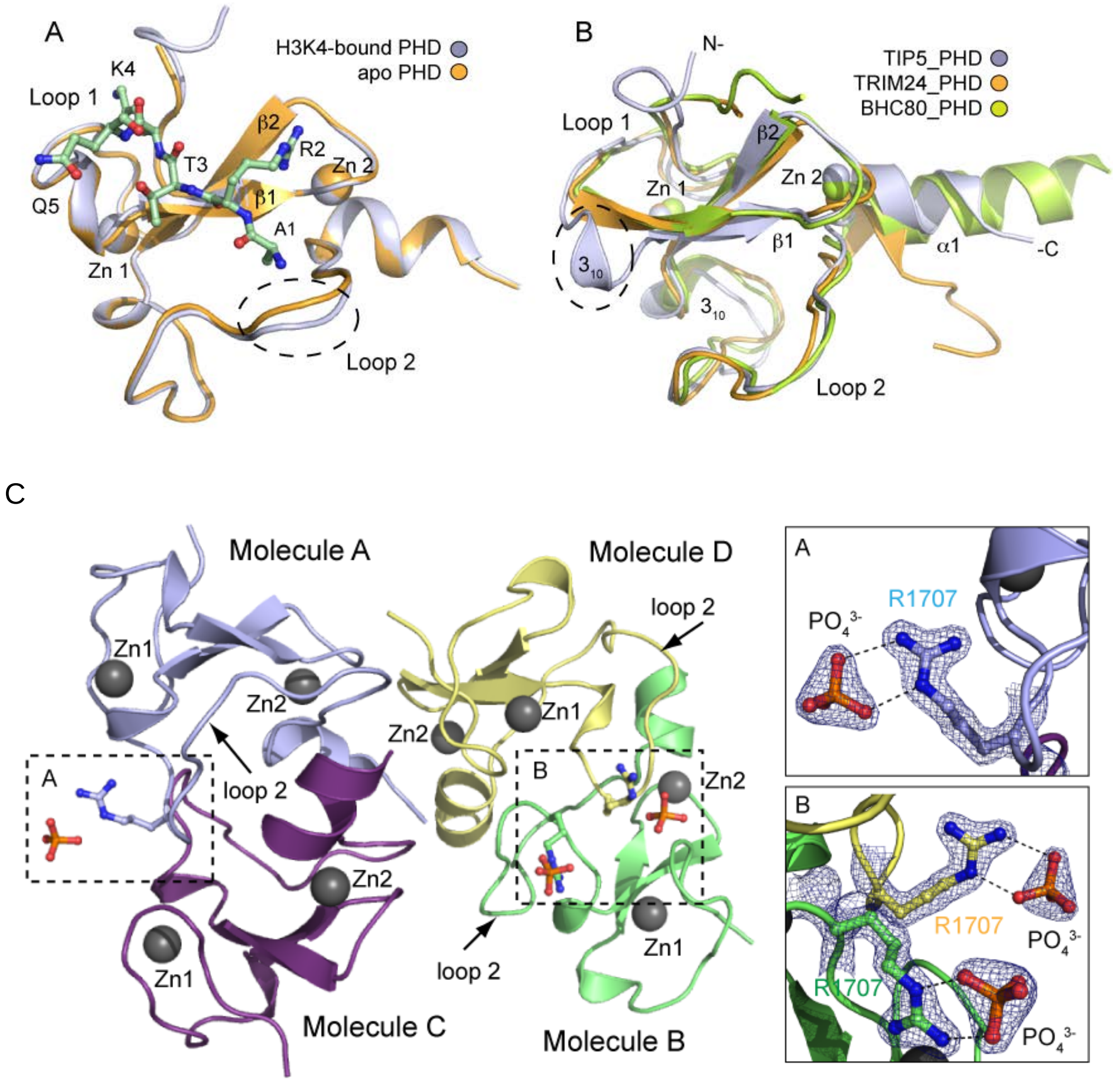

Figure S4 (related to Figure 2 and Table 2). **A.** TIP5 PHD zinc finger Cα superposition of free state and in complex with H3K4. **B**. Superposition of TIP5, TRIM24, BHC80 in complex structures (the H3K4 peptide has been omitted for the figure to highlight the difference in the surface-exposed cavity). **C.** The asymmetric unit of TIP5 PHD zinc finger in the free state. The four molecules that constitute the asymmetric unit are shown in different colours and displayed in cartoon representation. The exposed side chain of R1707 located at the beginning of loop 2 shows promiscuous affinity towards phosphate molecules from the crystallization buffer.

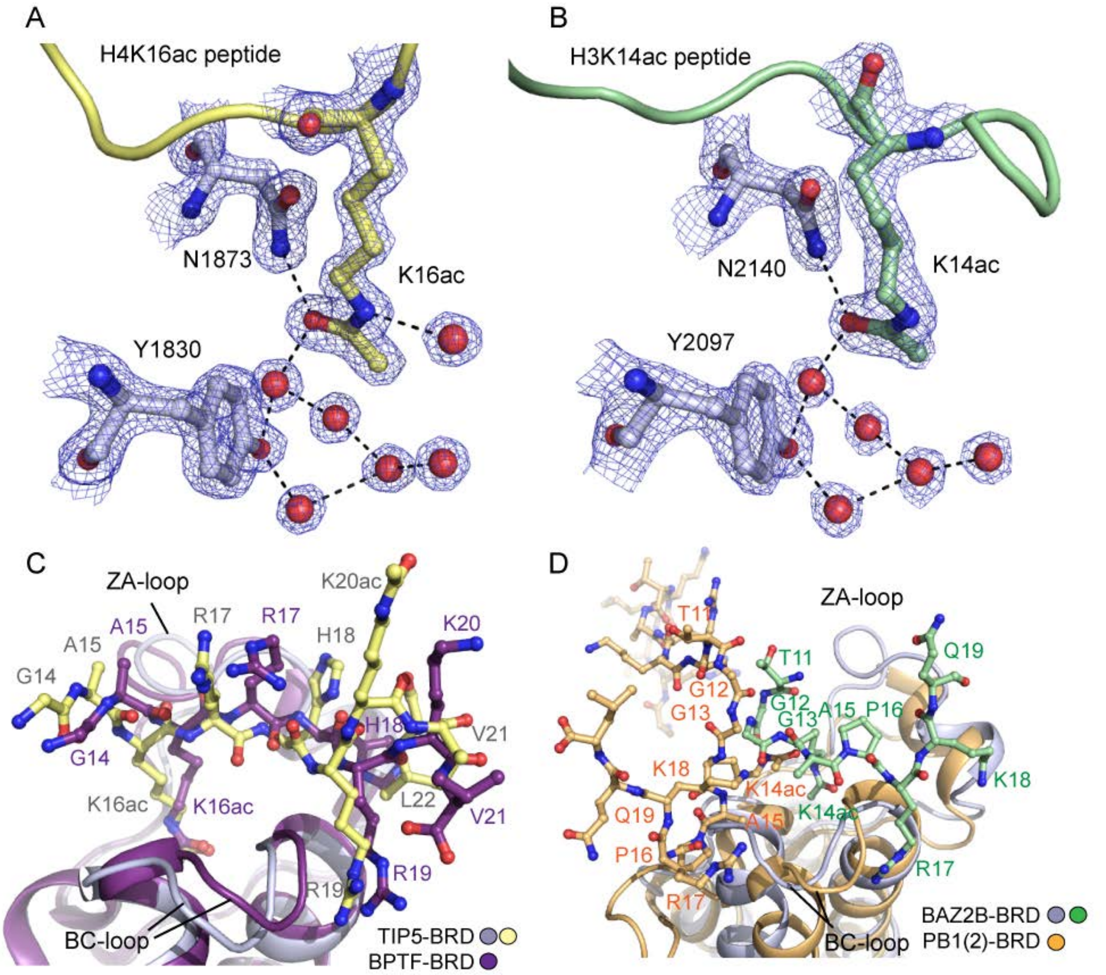

Figure S5 (related to Figure 3 and Table 2). A, B. Zoom in of the N-acetyl binding site displaying 2Fo-Fc electron density contoured at 1.8 σ. The acetyl group is surrounded by a ring of water molecules (red spheres) in both complex structures of TIP5 + H4K16ac (A) and BAZ2B + H3K14ac (B). **C.** Superposition of co-crystal structures of TIP5 and BPTF (PDB: 3QZS) on bromodomain Cα positions. **D.** Superposition of co-crystal structure of BAZ2B and solution structure of PB1 domain 2 (PDB: 2KTB) on bromodomain Cα positions.

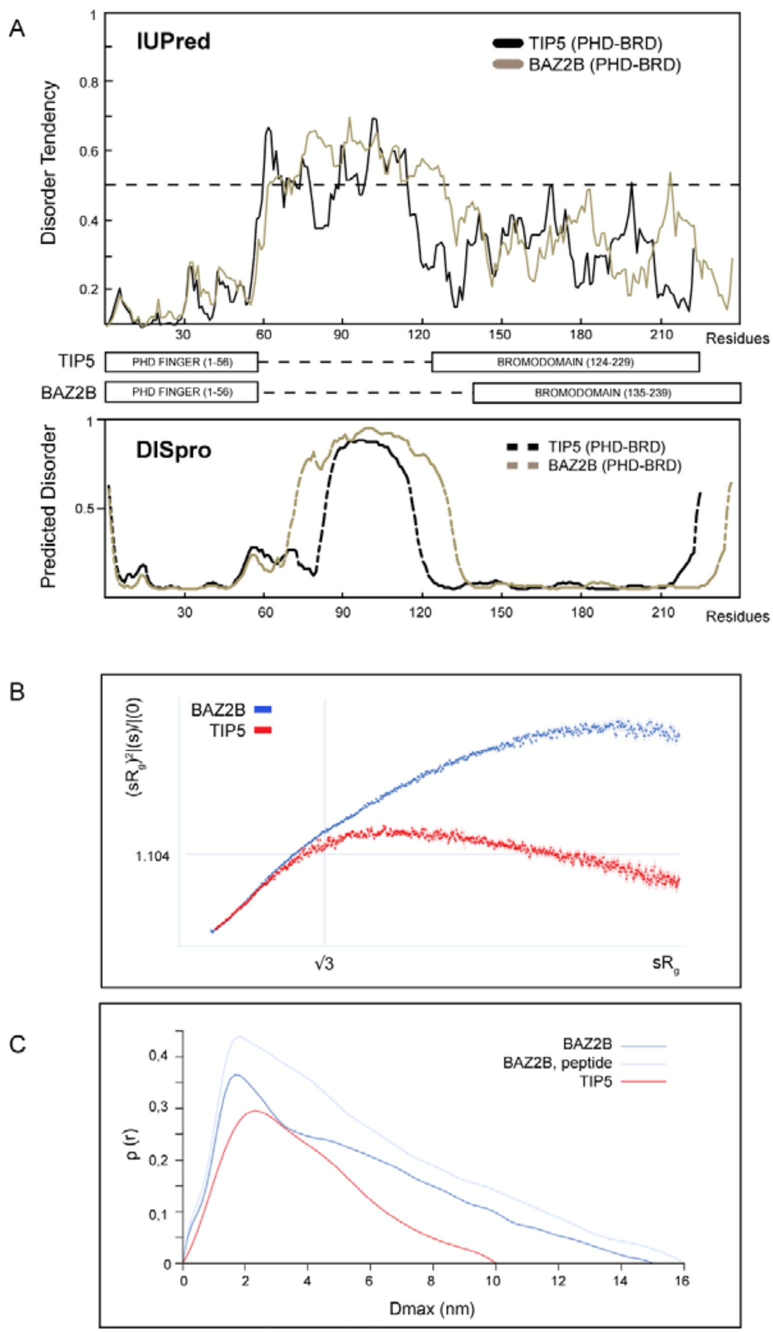

Figure S6 (related to Figure 5). Predicted disordered regions of the C-terminus part of human TIP5 and BAZ2B proteins (PHD-Bromodomain). Top panel: disorder output from IUPred software, which is able to predict intrinsically unstructured regions based on estimated energy content from amino acid composition. Bottom panel: *ab initio* prediction of disordered regions with DISpro, which is based on evolutionary information, predicted secondary structure and relative solvent accessibility. **B.** Kratky plots of the experimental data for TIP5 (red curve) and BAZ2B (blue curve) tandem domain in the free state. **C.** Distance distribution function of TIP5 and BAZ2B tandem domains in the free form (red and dark blue, respectively) and BAZ2B in complex with the peptide (light blue).

Table S1 (related to Figure 5). Statistics of the SAXS data collected at X33-DORIS and P12-PETRA III beamlines for BAZ2B and TIP5, respectively. Included are the data collection parameters, structural parameters, molecular mass determination and the software employed for the data processing and model determination.

Table S1: SAXS Data-collection and scattering-derived parameters

**Data collection parameters**	Apo TIP5 (PHD-BRD)	Apo BAZ2B (PHD-BRD)	Complex BAZ2B (PHD-BRD) with H3K14ac
Instrument	P12 (PETRA III)	X33 (DORIS)	X33 (DORIS)
Beam geometry (mm^2)	0.2 x 0.12	2 × 0.6	2 × 0.6
Wavelenght (Å)	1.24	1.5	1.5
q range ($Å^{-1}$)	0.007 – 0.465	0.009 - 0.601	0.009 - 0.601
Concentration range (mg ml^{-1})	0.7 - 9.6	1.2 - 10.7	2 - 6
Temperature (K)	283	283	283
**Structural parameters**			
I (0) (A.U.) [from P(r)]	1800 ± 100	28.5 ± 0.5	37.5 ± 0.5
R_g (Å) [from P(r)]	31 ± 1	42 ± 1	45 ± 1
I (0) (A.U.) [from Guinier]	1800 ± 100	28.0 ± 0.5	37.5 ± 0.5
R_g (Å) [from Guinier]	30 ± 1	40 ± 1	42 ± 1
D_{max} (Å)	100 ± 10	145 ± 10	160 ± 10
Porod volume estimate ($Å^3$)	45000 ± 2000	43000 ± 2000	52000 ± 2000
**Molecular-mass determination**			
Partial specific volume ($cm^3 g^{-1}$)	0.724	0.724	0.724
Contrast ($\Delta\rho \times 10^{10}$ cm^{-2})	3.047	3.047	3.047
Molecular mass M_r [from *I* (0)]	28000 ± 5000	33000 ± 5000	45000 ± 5000
Molecular mass M_r [from Porod volume]	26000 ± 5000	25000 ± 5000	31000 ± 5000
Calculated monomeric M_r from sequence	26718	27678	32400
**Software employed**			
Primary data reduction	PRIMUS	PRIMUS	PRIMUS
Data processing	AUTOGNOM	AUTOGNOM	AUTOGNOM
Ab initio analysis	DAMMIF	DAMMIF	DAMMIF
Validation and averaging	DAMAVER	DAMAVER	DAMAVER
Rigid-body modeling	BUNCH	EOM	N/A
Computation of model intensities	CRYSOL	N/A	N/A
Three-dimensional graphic representations	PyMOL	PyMOL	PyMOL

EXTENDED EXPERIMENTAL PROCEDURES

Constructs

The cDNAs encoding tandem PHD zinc finger and bromodomain of human TIP5 (residues 1673–1901, UniProt: Q9UIF9) and human BAZ2B (residues 1928-2168, GeneBank: Q9UIF8) were synthesized by GenScript with codons optimized for *E. coli.* expression and were subcloned into a modified pET15 vector using NdeI/XhoI sites for expression as recombinant proteins with a His6 tag fused with small ubiquitin like modifier (SUMO)-1 fusion tag at the N terminus. cDNA sequences were used as a template to amplify single PHD zinc fingers (residues 1673-1728 for TIP5; residues 1928-1983 for BAZ2B) and bromodomains (residues 1797-1899 for TIP5, residues 2054-2168 for BAZ2B) regions. Single PHD zinc fingers were introduced into the same modified SUMO tagged plasmid whereas single bromodomains were further subcloned into pET28 derived expression vectors, pNIC28-Bsa4 using ligation independent cloning (Stols et al., 2002).

Protein Expression and Purification

Best expression constructs encoding all target proteins were transformed into *E. coli* competent BL21(DE3) Rosetta cells (Invitrogen) or into BL21 (DE3)-R3-pRARE2 cells (phage-resistant derivative with a pRARE plasmid encoding rare codon tRNAs). Cells were grown at 37 ºC in Terrific Broth (Sigma) from overnight cultures until A600 reached between 0.6-0.8, then the media was cooled and 0.2 mM isopropyl-β-Dthiogalactopyranoside (IPTG) was added to induce the protein expression at 20 ºC for 16 hours. An extra 0.1 mM $ZnCl_2$ was added in the protein expression to those targets that contained a PHD zinc finger.

Different purification procedures have been optimized for each target protein. In common, *E. coli* overexpressed cells were lysed using an EmulsiFlex-C5 high-pressure homogenizer (Avestin - Mannheim, Germany) or high-pressure cell disruption (Constant Systems Limited) in loading buffer A from the nickel affinity column (HisTrap Chelating FF 5ml) in the presence of Protease Inhibitor Cocktail EDTA-free (Roche). Lysates were cleared by centrifugation (14,000 x g for 45min at 4 ºC, JLA 16.250 rotor, on a Beckman Coulter Avanti J-20 XP centrifuge). After centrifugation, the supernatant was loaded onto the nickel column and eluted in an imidazole linear gradient. The

eluted proteins were collected and treated overnight with SENP1 (SUMO endoprotease-1) or TEV (Tobacco Etch Virus) proteases at 4 ºC to remove their respective N terminal tags. Digested proteins were loaded onto a nickel column again to remove the cleaved 6His-SUMO tag and the hexa-histidine expression tag proteases used. The flow through containing the untagged proteins were collected and further dialysed and purified by an ion exchange chromatography (RESOURCE Q 6 ml or RESOURCE S 6 ml GE Healthcare Life Sciences), depending on the target protein). Proteins were eluted in a linear gradient of NaCl. Finally, a polishing step was performed through a size exclusion chromatography (HiLoad 16/600 Superdex 75 or 200 GE Healthcare Life Sciences). The major peak was pooled and concentrated to high concentration in order to set up crystallization trials. ESI-MS (electrospray ionization mass spectrometry) analysis of the purified samples was performed under native conditions to elucidate the oligomerization state of the tandem domain and the single PHD zinc finger domain (both length construct). For all cases, spectra showed unambiguously monomer species corresponding to their molecular weights. TIP5 and BAZ2B tandem protein have molecular weights of 26828.44 ± 17.13 Da and 27788.94 ± 14.66 Da.

Crystallization

Crystallization of PHD fingers of TIP5 and BAZ2B in the free-form: Crystals of the free-form of single TIP5 and BAZ2B PHD Zinc fingers were grown by mixing equal volumes of 6 and 5 mg/ml protein, respectively, and crystallization buffer (2.2-1.8 M Na/K phosphate pH 8) at 20 ºC. In follow-up screens, we obtained crystals appearing after two days and reaching their full lengths within a week. In both cases, the protein buffer was 20 mM Tris·HCl pH 8, 200 mM NaCl, 2 mM DTT, 20 µM $ZnCl_2$. The crystals were flash-frozen at 100 K in a nitrogen gas stream in the cryoprotectant with 20 % glycerol.

Histone peptide H3K4 complex crystallization with TIP5 PHD finger: peptide was soaked into pre-formed apo TIP5 PHD finger crystals grown in 2 M malic acid pH 7 as crystallization condition. Soaking solution contained 1.6 M malic acid and 12 mM H3K4 5-mer peptide and the crystals were soaked for > 12h at 20ºC. Crystals were cryo-protected in 1.4 M malic acid, 6 mM H3K4 and 20 % ethyleneglycol.

Crystallization of Bromodomain of TIP5 in the free-form: clusters of thin needle-shaped crystals of the free-form of single TIP5 bromodomain were obtained by mixing equal volumes of 23 mg/ml and crystallization buffer (0.1 M Tris·HCl pH 8.4, 0.25 M $MgCl_2$, 22 % PEG 3350) at 4ºC. Several rounds of macro-seeding were needed to obtain

thicker and isolated bar-shaped crystals using the sitting drop vapour diffusion method. The protein buffer was 20 mM Tris·HCl pH 7.5, 200 mM NaCl, 2 mM DTT. A single crystal was flash-frozen at 100 K in a nitrogen gas stream in cryoprotectant solution containing 15 % ethylene glycol.

Histone peptide H4K16acK20ac complex crystallization with TIP5 bromodomain: protein was concentrated up to 17 mg/ml and co-crystallised with 1:5 M excess of the 9-mer peptide in 150 nl + 150 nl + 20 nl seeds (from 2nd round) sitting drop at 4 ºC. Prism-like crystals appeared in 2-3 days and the seeding helped to prevent twinning issues. The optimised crystallisation conditions were 0.1 M potassium phosphate monobasic, 0.1M MES pH 6.5, 2 M sodium chloride. 20 % glycerol was used as the cryoprotectant.

Histone peptides H3K14ac and H4K8acK12ac complex crystallization with BAZ2B bromodomain: Protein was concentrated up to 23 mg/ml and co-crystallised with 1:5 M excess of each peptide in 150 nl + 150 nl sitting drop at 4 ºC. The optimised crystallisation conditions were 0.1 M MES pH 5.9, 0.01m zinc sulphate, 25 % PEG methyl ether 550 for H3K14ac complex crystals; 0.1M cacodylate pH 6.5, 0.2 M calcium acetate, 18 % PEG8000 for H4K8acK12ac complex crystals; 20 % glycerol was used as the cryoprotectant.

SUPPLEMENTAL REFERENCES

Chakravarty, S., Zeng, L., and Zhou, M.M. (2009) Structure and site-specific recognition of histone H3 by the PHD finger of human autoimmune regulator. Structure *17*, 670-679.

Fiedler, M., Sanchez-Barrena, M.J., Nekrasov, M., Mieszczanek, J., Rybin, V., Muller, J., Evans, P., and Bienz, M. (2008). Decoding of methylated histone H3 tail by the Pygo-BCL9 Wnt signaling complex. Mol Cell *30*, 507-518.

Lan, F., Collins, R.E., De Cegli, R., Alpatov, R., Horton, J.R., Shi, X., Gozani, O., Cheng, X., and Shi, Y. (2007). Recognition of unmethylated histone H3 lysine 4 links BHC80 to LSD1-mediated gene repression. Nature *448*, 718-722.

Li, H., Ilin, S., Wang, W., Duncan, E.M., Wysocka, J., Allis, C.D., and Patel, D.J. (2006). Molecular basis for site-specific read-out of histone H3K4me3 by the BPTF PHD finger of NURF. Nature *442*, 91-95.

Mansfield, R.E., Musselman, C.A., Kwan, A.H., Oliver, S.S., Garske, A.L., Davrazou, F., Denu, J.M., Kutateladze, T.G., and Mackay, J.P. (2011) Plant homeodomain (PHD) fingers of CHD4 are histone H3-binding modules with preference for unmodified H3K4 and methylated H3K9. J Biol Chem *286*, 11779-11791.

Ooi, S.K., Qiu, C., Bernstein, E., Li, K., Jia, D., Yang, Z., Erdjument-Bromage, H., Tempst, P., Lin, S.P., Allis, C.D., *et al.* (2007). DNMT3L connects unmethylated lysine 4 of histone H3 to de novo methylation of DNA. Nature *448*, 714-717.

Palacios, A., Munoz, I.G., Pantoja-Uceda, D., Marcaida, M.J., Torres, D., Martin-Garcia, J.M., Luque, I., Montoya, G., and Blanco, F.J. (2008). Molecular basis of histone H3K4me3 recognition by ING4. J Biol Chem *283*, 15956-15964.

Pascual, J., Martinez-Yamout, M., Dyson, H.J., and Wright, P.E. (2000). Structure of the PHD zinc finger from human Williams-Beuren syndrome transcription factor. J Mol Biol *304*, 723-729.

Pena, P.V., Hom, R.A., Hung, T., Lin, H., Kuo, A.J., Wong, R.P., Subach, O.M., Champagne, K.S., Zhao, R., Verkhusha, V.V.*, et al.* (2008). Histone H3K4me3 binding is required for the DNA repair and apoptotic activities of ING1 tumor suppressor. J Mol Biol *380*, 303-312.

Petoukhov, MV., Franke, D., Shkumatov, AV., Tria, G., Kikhney AG., Gajda, M., Gorba, C., Mertens HDT., Konarev, PV., and Svergun DI. (2012) New developments in the *ATSAS* program package for small-angle scattering data analysis. J. Appl. Cryst. 45, 342-350.

Ramon-Maiques, S., Kuo, A.J., Carney, D., Matthews, A.G., Oettinger, M.A., Gozani, O., and Yang, W. (2007). The plant homeodomain finger of RAG2 recognizes histone H3 methylated at both lysine-4 and arginine-2. Proc Natl Acad Sci U S A *104*, 18993-18998.

Stols, L., Gu, M.Y., Dieckman, L., Raffen, R., Collart, F.R., and Donnelly, M.I. (2002). A new vector for high-throughput, ligation-independent cloning encoding a tobacco etch virus protease cleavage site. Protein Expr. Purif. 25, 8–15.

Wang, Z., Song, J., Milne, T.A., Wang, G.G., Li, H., Allis, C.D., and Patel, D.J. (2010) Pro isomerization in MLL1 PHD3-bromo cassette connects H3K4me readout to CyP33 and HDAC-mediated repression. Cell *141*, 1183-1194.

Wen, H., Li, J., Song, T., Lu, M., Kan, P.Y., Lee, M.G., Sha, B., and Shi, X. (2010) Recognition of histone H3K4 trimethylation by the plant homeodomain of PHF2 modulates histone demethylation. J Biol Chem *285*, 9322-9326.

Zeng, L., Zhang, Q., Li, S., Plotnikov, A.N., Walsh, M.J., and Zhou, M.M. (2010) Mechanism and regulation of acetylated histone binding by the tandem PHD finger of DPF3b. Nature *466*, 258-262.
