# Supplementary material for: Molecular Basis of Histone Tail Recognition by Human TIP5 PHD Finger and Bromodomain of the Chromatin Remodeling Complex NoRC
Source: Structure. 2015 Jan 6;23(1):80–92. doi: 10.1016/j.str.2014.10.017 (PMC4291147; doi:10.1016/j.str.2014.10.017)
Supplement: Document S2. Article plus Supplemental Information [file mmc2.pdf]

Structure
Article

Molecular Basis of Histone Tail Recognition by Human TIP5 PHD Finger and Bromodomain of the Chromatin Remodeling Complex NoRC

Cynthia Tallant,[1,2,3] Erica Valentini,[4] Oleg Fedorov,[2,3] Lois Overvoorde,[1] Fleur M. Ferguson,[1] Panagis Filippakopoulos,[2,5] Dmitri I. Svergun,[4] Stefan Knapp,[2,3] and Alessio Ciulli[1,6,*]
[1]Department of Chemistry, University of Cambridge, Lensfield Road, Cambridge CB2 1EW, UK
[2]Structural Genomics Consortium, Nuffield Department of Clinical Medicine, University of Oxford, Old Road Campus Research Building, Roosevelt Drive, Oxford OX3 7DQ, UK
[3]Target Discovery Institute, Nuffield Department of Clinical Medicine, University of Oxford, NDM Research Building, Roosevelt Drive, Oxford OX3 7FZ, UK
[4]European Molecular Biology Laboratory, Hamburg Outstation, c/o DESY, Notkestrasse 85, 22603 Hamburg, Germany
[5]Ludwig Institute for Cancer Research, Nuffield Department of Clinical Medicine, University of Oxford, Roosevelt Drive, Oxford OX3 7DQ, UK
[6]Division of Biological Chemistry and Drug Discovery, College of Life Sciences, University of Dundee, Dow Street, Dundee DD1 5EH, UK
*Correspondence: a.ciulli@dundee.ac.uk
http://dx.doi.org/10.1016/j.str.2014.10.017
This is an open access article under the CC BY license (http://creativecommons.org/licenses/by/3.0/).

SUMMARY

Binding of the chromatin remodeling complex NoRC to RNA complementary to the rDNA promoter mediates transcriptional repression. TIP5, the largest subunit of NoRC, is involved in recruitment to rDNA by interactions with promoter-bound TTF-I, pRNA, and acetylation of H4K16. TIP5 domains that recognize posttranslational modifications on histones are essential for recruitment of NoRC to chromatin, but how these reader modules recognize site-specific histone tails has remained elusive. Here, we report crystal structures of PHD zinc finger and bromodomains from human TIP5 and BAZ2B in free form and bound to H3 and/or H4 histones. PHD finger functions as an independent structural module in recognizing unmodified H3 histone tails, and the bromodomain prefers H3 and H4 acetylation marks followed by a key basic residue, KacXXR. Further low-resolution analyses of PHD-bromodomain modules provide molecular insights into their *trans* histone tail recognition, required for nucleosome recruitment and transcriptional repression of the NoRC complex.

INTRODUCTION

Chromatin remodeling complex NoRC (*n*ucleolar *r*emodeling *c*omplex) interacts with the rDNA promoter regions of silent genes repressing rDNA transcription. It consists of TIP5 (*T*TF-I *i*nteracting *p*rotein *5*), also known as BAZ2A (*b*romodomain [BRD] *a*djacent to *z*inc finger domain protein *2A*), which is the largest subunit, and the adenosine triphosphatase (ATPase) SNF2h (*s*ucrose *n*on*f*ermenting protein *2* *h*omologue). Human TIP5 is a 1,908 amino acid multidomain protein of a molecular weight of 211 kDa and consists of several protein and DNA interaction domains (Grummt, 2007). The biological function of the related human 240 kDa protein BAZ2B (*B*RD *a*djacent to *z*inc finger domain protein *2B*) remains unknown, although it is postulated to regulate nucleosome mobilization through the ATP-dependent chromatin remodeling factor ISWI (*i*mitation *swi*tch), resulting in transcriptional regulation (Eberharter et al., 2004). BAZ2B shares 29% sequence identity with TIP5 at the full-length level and harbors similar domain organization. Both proteins contain a MBD (*m*ethyl-CpG-*b*inding *d*omain), also known as TAM (*T*TF-IIP5, *A*RBP, *M*eCP1) domain, AT-hook DNA-binding domains, DDT (*D*NA binding homeobox and *d*ifferent *t*ranscription factors), and a PHD (*p*lant *h*omeo*d*omain) zinc finger adjacent to a BRD at their C terminus (Figure 1). Sequence similarity reaches up to 50% to 65% for individual PHDs and BRDs, suggesting related roles of these two proteins (Figure S1A available online). These functional domains are also shared with ACF1 (*A*TP-utilizing *c*hromatin assembly and remodeling *f*actor) and WSTF (*W*illiams *s*yndrome *t*ranscription *f*actor), two large subunits of human chromatin remodeling complexes hCHRAC (*h*uman *ch*romatin *a*ccessibility *c*omplex) and hWICH (*h*uman *W*STF-*I*SWI *ch*romatin remodeling complex) (Wu et al., 2009). A recent study determined how the DNA-binding TAM and AT-hook components of TIP5 increase the association of rDNA with the nuclear matrix regulating large-scale chromatin organization (Zillner et al., 2013). On the other hand, it has been shown that the tandem PHD finger-BRD modules of human TIP5 represent an independent unit that interacts with H4K16ac and triggers the cascade of events that establish the repressed state of rDNA (Zhou and Grummt, 2005). Zhou and coworkers reported that inactive TIP5 BRD mutant at the conserved tyrosine does not repress Pol I transcription and suggested that interaction of the BRD with acetylated histone H4 is a key event in NoRC-mediated gene silencing. Additionally, nuclear localization assays and chromatin immunoprecipitation sequencing with deletion constructs of the PHD finger-BRD region have established a role for TIP5 abrogating NoRC-mediated transcriptional repression and heterochromatic features. Indeed, the PHD zinc finger adjacent to the BRD has an important role for the NoRC function, binding to DNMTs (*DN*A *m*ethyl*t*ransferases),

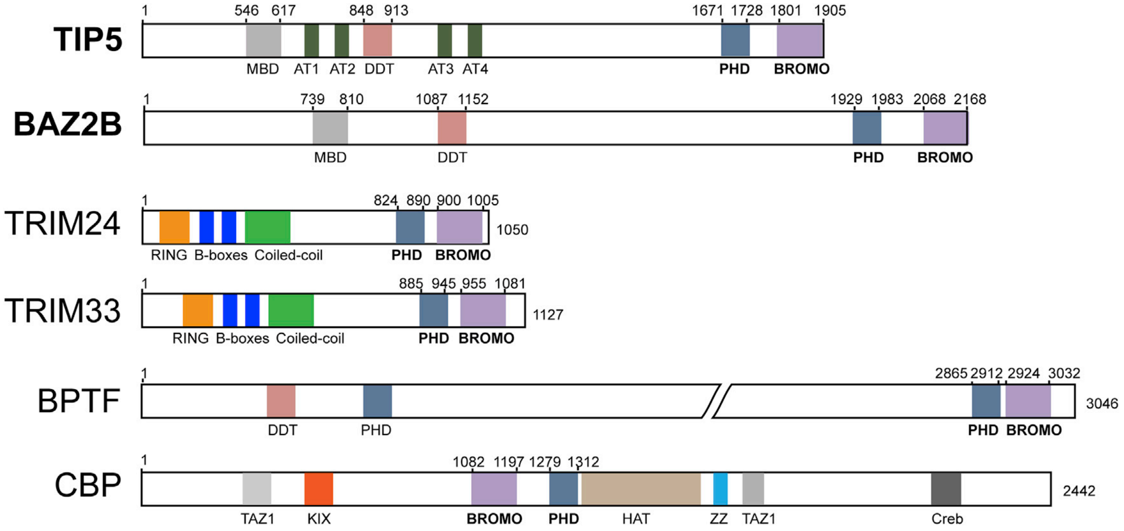

Figure 1. Domain Organization of Human Chromatin Associated Proteins Containing PHD Zinc Fingers Adjacent to BRDs
See Figure S1.

HDAC1 (*h*istone *deac*etylase *1*), and the ATPase subunit SNF2h, further establishing heterochromatic features (including DNA methylation, hypoacetylation of histone H4 and methylation of H3K9) at the rDNA promoter (Zhou et al., 2009). However, structural elucidation of the tandem PHD-BRD reader modules of TIP5 and BAZ2B as well as their recognition specificity toward histone tails have to date remained elusive.

PHDs represent a diverse family of protein interaction domains that may specifically recognize methylated or unmodified histone tails, whereas BRDs recognize their target sequences usually in an acetylation-dependent manner (Sanchez and Zhou, 2011; Filippakopoulos et al., 2012). Coexistence of PHD-BRD module is evolutionary conserved and considerably more frequent than the pairing of other epigenetic effector domains (Figure 1). Synergistic combinatorial readout of epigenetic marks by PHD zinc finger and BRD modules has been characterized in several chromatin-associated proteins (Figure S1B). Structural studies on the tripartite motif (TRIM)-containing proteins revealed single tightly linked functional PHD-BRD units and a multivalent recognition on the same H3 histone tail mediated by interaction with unmodified K4 and acetylated K23 (for TRIM24) and unmodified K4, trimethylated K9, and acetylated K18 (for TRIM33) (Tsai et al., 2010; Xi et al., 2011). In contrast, interaction with two histones (H3 and H4) has been reported for the tandem PHD-BRD of BPTF (Ruthenburg et al., 2011), where the PHD finger recognizes methylation signatures at H3K4me2/3 and the BRD selectively binds to the H4K16ac acetylation mark. The simultaneous interaction with two histones is imparted by a rigid α-helical linker of 12 residues in length, preventing direct PHD-BRD interactions and enabling a trans histone intranucleosomal association. Another example of a BRD-PHD arrangement is found in the crystal structure of the human transcriptional activator CBP (Plotnikov et al., 2014) as well as in the structure of the entire HAT/RING/PHD-BRD unit, which exhibited tight integration of all domains in a single structural assembly (Delvecchio et al., 2013). Thus, the conserved PHD-BRD module constitutes a diverse structural scaffold for recognition of one or two histones depending on the spatial organization of the individual interaction modules (Figure S1B).

The BAZ2 tandem PHD-BRD module is characterized by a long linker region. Here we report the high-resolution crystal structures of the PHD finger and BRD of TIP5 and BAZ2B in complex with their specific target sequences identified by histone peptide array screens. Our crystal structures combined with biophysical and mutagenesis data demonstrate that PHD zinc fingers recognize N-terminal unmodified H3K4 tail, whereas the TIP5 and BAZ2B BRDs recognize H4K16ac and H3K14ac, respectively, with specificity pattern KacXXR. Low-resolution small-angle X-ray scattering (SAXS) studies in solution show that both domains are loosely tethered by a flexible linker region and act as structurally independent units.

RESULTS AND DISCUSSION

Histone Peptide Recognition of TIP5 and BAZ2B

Systematic biophysical screening and validation were carried out in order to determine posttranslational modifications (PTMs) on histones that are recognized by the BAZ2 PHD finger and BRD modules. We used biolayer interferometry (BLI) as a biosensor technology to monitor and quantify interactions of biotinylated histone peptides immobilized on optical fiber streptavidin-coated biosensor tips with individual and tandem PHD finger and BRDs of TIP5 and BAZ2B in solution. Interestingly, when analyzing PTMs in histone 3 and 4 (Figures S2A and S2B), we observed a stronger response for unmodified H3 peptides and decreasing binding with increasing K4 methylation levels (K4 $\geq$ K4me > K4me2 > K4me3) (Figures S2C–S2F). The binding data generated on isolated PHD fingers suggested that these reader domains preferentially recognize unmodified K4. Additionally, using either single PHD finger and the dual-domain construct, acetylation on H3K9ac and phosphorylation of H3S10 and H3T11 increased binding response. The TIP5 and BAZ2B

Table 1. Summary of Thermodynamic Analysis

Histone Peptides	Protein	K_D (μM)	N	ΔH (kcal/mol)	TΔS (kcal/mol)	ΔG (kcal/mol)
H3(1–9)	TIP5 PHD	2.51 ± 0.63	0.928 ± 0.002	−11.510 ± 0.041	−3.874	−7.636
	BAZ2B PHD	3.07 ± 0.71	1.016 ± 0.003	−9.809 ± 0.038	−2.289	−7.520
H3(1–9)K4me	TIP5 PHD	2.50 ± 0.43	1.015 ± 0.003	−8.253 ± 0.040	−0.608	−7.645
	BAZ2B PHD	3.98 ± 0.61	1.211 ± 0.005	−6.717 ± 0.042	0.647	−7.364
H3(1–9)K4me3	TIP5 PHD	28.18 ± 0.34	1.101 ± 0.014	−3.549 ± 0.073	2.419	−6.202
	BAZ2B PHD	19.67 ± 0.21	1.020 ± 0.012	−6.567 ± 0.117	−0.143	−6.424
H3(11–20)	TIP5 PHD	ND	ND	ND	ND	ND
	BAZ2B PHD	ND	ND	ND	ND	ND
H3(9–19)K14ac	TIP5 BRD	33.38 ± 0.68	0.904 ± 0.014	−10.120 ± 0.208	−4.102	−6.018
	BAZ2B BRD	10.35 ± 0.20	1.050 ± 0.008	−9.147 ± 0.095	−2.455	−6.692
H4(1–10)K5acK8ac	TIP5 BRD	65.91 ± 0.46	0.554 ± 0.070	−7.780 ± 0.118	−2.171	−5.609
	BAZ2B BRD	ND	ND	ND	ND	ND
H4(6–13)K8acK12ac	TIP5 BRD	63.25 ± 0.11	0.639 ± 0.026	−5.963 ± 0.307	−0.331	−5.632
	BAZ2B BRD	ND	ND	ND	ND	ND
H4(9–17)K12acK16ac	TIP5 BRD	44.76 ± 0.73	1.087 ± 0.021	−3.839 ± 0.103	1.992	−5.831
	BAZ2B BRD	ND	ND	ND	ND	ND
H4(14–22)K16ac	TIP5 BRD	23.69 ± 0.62	1.186 ± 0.015	−12.740 ± 0.226	−6.533	−6.207
	BAZ2B BRD	46.82 ± 0.73	1.062 ± 0.064	−2.820 ± 0.223	2.989	−5.809
H4(14–22)K16acK20ac	TIP5 BRD	26.08 ± 0.37	0.984 ± 0.017	−15.400 ± 0.369	−9.259	−6.141
	BAZ2B BRD	42.41 ± 0.42	1.124 ± 0.033	−2.382 ± 0.097	3.4878	−5.869
H3(1–18)K14ac	TIP5 PHD-BRD	1.87 ± 0.29	1.238 ± 0.190	−10.350 ± 0.071	−3.106	−7.244
	BAZ2B PHD-BRD	2.33 ± 0.19	1.118 ± 0.014	−16.600 ± 0.037	−9.054	−7.546

ITC-based binding parameters for complex formation between different histone peptides and wild-type of TIP5 and BAZ2B single PHD and BRD proteins and tandem PHD-BRD proteins. Error values represent errors of the nonlinear least-squares fit. Data obtained for stoichiometry of n = 0.5 binding constants are likely to represent two independent binding events. Buffer conditions used for the titrations were 20 mM HEPES (pH 7.5–8), 150 mM NaCl, and 0.5 mM TCEP. See also Figure S2). ND, not determined.

BRDs recognized preferentially the K14ac mark from a systematic H3 PTM array, demonstrating interactions of the TIP5 BRD with acetylated H3 and confirming previous studies showing recognition of this mark by the BAZ2B BRD (Filippakopoulos et al., 2012; Philpott et al., 2011). Calorimetric results showed 3-fold stronger potency of H3K14ac peptide for BAZ2B (K_D = 10 μM) than for TIP5 (K_D = 33 μM) BRD (Table 1). Regarding H4 histone Kac recognition, the TIP5 BRD was more promiscuous than the BAZ2B. We detected significant interaction of the TIP5 BRD with singly acetylated peptides of H4 K5, K8, K12, K16, and K20 and increased affinities for peptides harboring multiple acetylation sites (Figure S2B). Isothermal titration calorimetry (ITC) data suggest a preference of the TIP5 BRD for K16ac (Table 1). In a similar fashion, an unexpected and significant interaction with H2A acetylation marks was evident in experiments using both the tandem PHD-BRD and the isolated BRD of TIP5 in our BLI study. Additionally, we analyzed the likelihood of single *cis*-histone engagement of tandem domains comparing combinations of marks and unmodified peptides sequences localized on the same peptide. The strongest interaction was observed for peptides containing H3K14ac and unmodified K4. Further calorimetric analysis confirmed a single-digit micromolar binding, with K_D values of 2 μM for TIP5 and BAZ2B (PHD-BRD) for the peptide H3K14ac (1–18). These data are not consistent with a dual recognition for the tandem domain due to marginal potency enhancement. In contrast, binding studies with *cis*-reading PHD-BRD-related human proteins such as TRIM24 and TRIM33 confirmed a combinatorial readout in which the binding affinity was 90-fold increased for histone H3 (residues 1–33) bearing unmodified H3K4 and H3K23ac mark in comparison with shorter H3 peptides with individual marks for TRIM24 (Tsai et al., 2010) and strongest binding to H3 (1–28) containing H3K4ac and K18ac for TRIM33 (Xi et al., 2011).

Histone Recognition of the NoRC PHD Zinc Finger: Structural Insights with Unmodified H3 Histone

In order to gain structural insight into the histone recognition of NoRC, we determined the crystal structures of the PHD zinc fingers of human TIP5 and BAZ2B in the free state as well as in complex with N-terminal H3 histone peptide in the case of TIP5 (Table 2). Human TIP5 and BAZ2B adopt the canonical PHD finger "cross-braced" topology with two zinc-binding sites coordinated by the Cys4-His-Cys3 motif (Figures 2A and 2B). Interestingly, TIP5 and BAZ2B PHD fingers adopted a right-handed 3_{10} helix localizing residues D1688, E1689, F1670 and E1943, E1944, and L1945, respectively, into the binding surface. This is a key structural feature to modulate the H3-binding site. A peptide soak of unmodified H3 histone to apo crystals allowed us to determine the peptide-binding at high resolution (1.91 Å) (Figure 2E). The structure of the complex revealed that the N terminus of H3 is recognized by main chain hydrogen-bond interactions with backbone carbonyl groups of P1714, E1715,

CellPress

Table 2. Crystallographic Data Collection and Refinement Statistics

Protein ID	TIP5 PHD	TIP5 PHD	TIP5 BRD	TIP5 BRD	BAZ2B PHD	BAZ2B BRD	BAZ2B BRD
Ligand		H3K4		H4K16acK20ac		H3K14ac	H4K8acK12ac
Data Collection							
Space group	$P4_32_12$	$P4_32_12$	$P3_12$ 1	$P4_1$	$P2_12_12_1$	$P2_12_12_1$	$P2_1$
Cell dimensions							
a, b, c (Å)	72.01, 72.01, 99.06	71.87, 71.87, 98.77	95.42, 95.42, 33.05	64.87, 64.87, 71.64	38.49, 45.60, 65.25	29.72, 67.01, 136.10	61.67, 32.40, 62.03
α, β, γ (°)	90.0, 90.0, 90.0	90.0, 90.0, 90.0	90.0, 90.0, 120.0	90.0, 90.0, 90.0	90.0, 90.0, 90.0	90.0, 90.0, 90.0	90.0, 90.0, 90.0
Resolution (Å)[a]	29.12 (1.70)	29.06 (1.91)	27.54 (1.76)	29.01 (1.65)	29.41 (1.60)	29.04 (1.99)	29.18 (1.60)
Unique observations	28,791 (3,972)	20,562 (1,313)	17,392 (2,512)	35,633 (1,743)	15,620 (2,199)	19,403 (1,322)	30,743 (1,522)
Completeness (%)	98.0 (95.1)	99.8 (98.3)	100.0 (100.0)	99.6 (100.0)	99.6 (98.4)	99.7 (96.2)	99.4 (97.1)
Redundancy	14.8 (14.9)	7.0 (7.0)	9.9 (9.7)	8.5 (8.8)	5.4 (5.2)	8.4 (8.1)	4.3 (4.4)
R_{sym} or R_{merge}	0.063 (0.514)	0.062 (1.020)	0.063 (0.850)	0.067 (0.446)	0.029 (0.140)	0.068 (0.635)	0.019 (0.044)
$I/\sigma I$	24.9 (5.9)	20.4 (2.2)	20.0 (2.6)	15.6 (3.4)	29.7 (8.1)	17.6 (3.6)	45.4 (23.2)
Wavelength	1.2822	0.9796	0.9686	0.9796	1.2816	0.9796	0.9796
Phasing	Zn-SAD	MR	MR	MR	Zn-SAD	MR	MR
Refinement							
R_{work}/R_{free} (%)	18.22/21.50	18.29/21.86	17.60/21.70	18.53/20.50	18.99/22.13	21.23/25.57	16.77/18.70
Number of atoms							
Protein/other/solvent	1635/29/120	1672/126/70	842/23/103	1763/166/224	897/4/77	1731/138/94	1756/61/275
B factors ($Å^2$)							
Protein/other/solvent	31.2/41.6/32.6	35.6/53.7/35.7	39.6/49.9/40.7	22.8/29.6/29.9	28.9/20.4/31.3	36.0/48.8/36.0	14.0/15.4/22.2
Rmsd bond (Å)	0.012	0.012	0.015	0.010	0.014	0.015	0.009
Rmsd angle (°)	1.926	1.553	1.617	1.386	1.660	1.658	1.307
Ramachandran Statistics							
Allowed (%)	100.00	100.00	100.00	100.00	100.00	100.00	100.00
Favored (%)	99.50	99.07	98.00	99.55	100.00	99.55	99.54
Outliers (%)	0.00	0.00	0.00	0.00	0.00	0.00	0.00
PDB accession number	4QF2	4Q6F	4LZ2	4QBM	4QF3	4QC1	4QC3

[a]Highest resolution shell shown in parentheses.

and G1716 located in loop 2 and by hydrophobic interaction of the side chain of H3A1, which was well accommodated in a hydrophobic pocket formed by L1692 and P1714 (Figure 2D). Peptide binding was associated with structural changes in loop 2, which was shifted about 2 Å in the peptide bound state (Figure S4A). As observed in other PHD finger complexes, the H3 peptide binds to the surface in an antiparallel β sheet conformation with the TIP5 β1 stabilized by backbone hydrogen bonds with residues L1693, L1691, and D1688. An important feature of the structure is the recognition of the H3K4 side chain, in which the N terminus and loop1 of the TIP5 PHD finger harbor the lysine side chain, forming a shallow but well-defined groove. The H3K4 epsilon amino group interacts through hydrogen bonds with two main chain carbonyl oxygens (V1677, G1685) and a long-range salt bridge (4.2 Å) with D1688 located in the TIP5 3_{10} helix. The limited space in the lysine binding cavity and the absence of the typical aromatic cage present in methyl-lysine interacting PHD domains restricts binding of this PHD finger to unmodified lysine side chains. However, our thermodynamic binding data suggests that binding to single methylation mark can still be accommodated with similar binding potency. ITC measurements using a histone H3 peptide containing K4, K4me, and K4me$_3$ resulted in K_D values of 2.51, 2.50, and 28.18 μM for TIP5 (Figure 2C) and 3.07, 3.98, and 19.67 μM for BAZ2B (Figure 2F). Therefore, increasing the degree of methylation resulted in a marked decrease in binding affinity, as suggested by the structure. However, despite similar binding affinity the enthalpic contribution to binding were more favorable for the unmodified K4, suggesting more optimal interaction. Additionally, on the basis of our biophysical screening (Figure S2A), methylation of R2 and acetylation of K9 did not significantly affect peptide binding. Interestingly, we observed a favorable increase of the affinity toward H3S10ph and H3T11ph but not to other phosphorylation marks. We examined the crystal packing of the TIP5 PHD finger in the free form and highlighted a phosphate ion establishing a solid attractive interaction with the guanidinium group of the

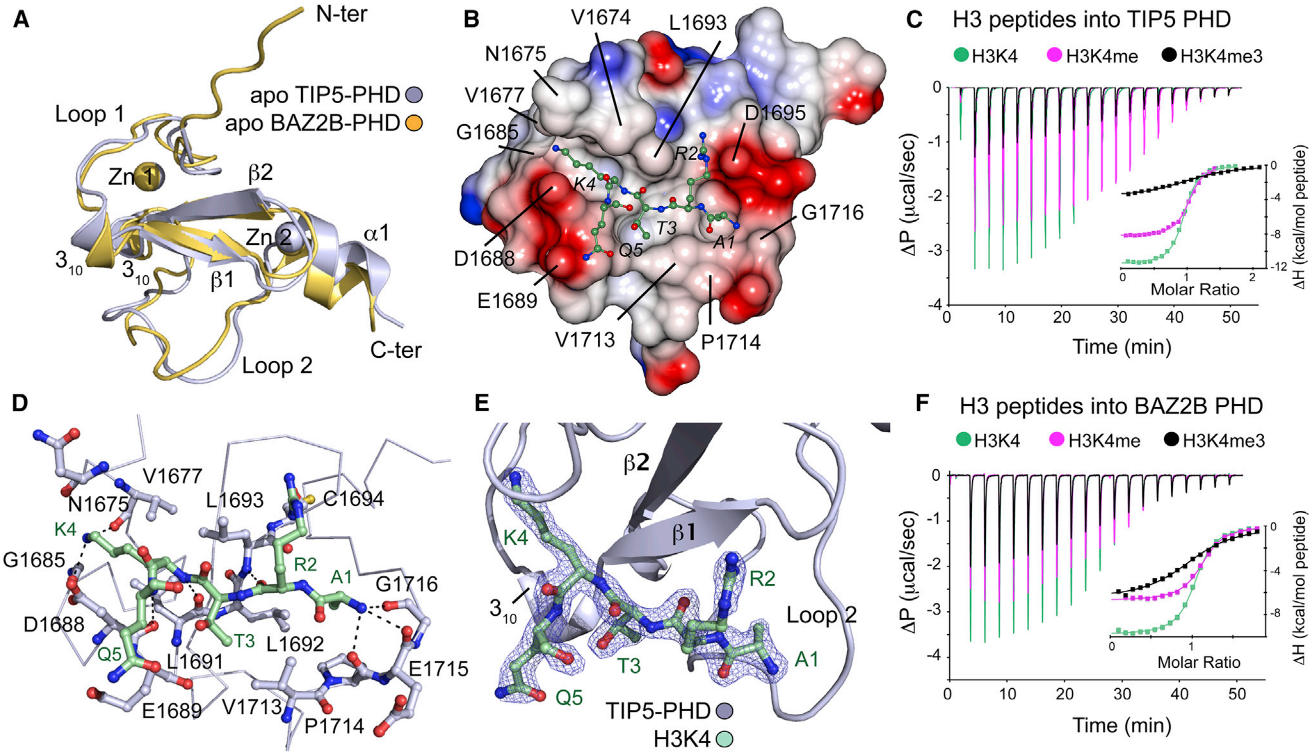

Figure 2. Structures of TIP5 and BAZ2B PHD Zinc Fingers in Free State and in Complex with H3K4 Peptide

(A) New crystal structures superposition on ribbon representation of TIP5 (PDB accession number 4QF2) and BAZ2B (PDB accession number 4QF3) PHD zinc fingers on Cα positions (rmsd = 1.595).

(B) Positioning of the unmodified peptide H3K4 (ART[**K**]Q) on the surface of TIP5. The binding interface of BAZ2B and TIP5 are characterized of being hydrophobic and slightly acidic. Regions of positive and negative electrostatic potential are shown in blue and red from +5 to −5 V, respectively (CCP4mg; McNicholas et al., 2011).

(D) Detailed interactions between PHD finger of TIP5 and H3K4 peptide.

(E) View of the peptide-binding pocket. Simulated-annealing 2Fo-Fc maps showing the electron density for the H3 peptide. Map was calculated at 1.91 Å resolution and contoured at 1.2 σ above the mean.

(C and F) ITC binding curves of PHD fingers from TIP5 and BAZ2B with H3 histone peptides bearing different PTM marks on K4. The resulting integrated ΔH data (kcal/mol) plotted versus the molar ratio of total ligand and protein concentrations are shown in the inset panel.

See also Figures S3 and S4.

same arginine residue (R1707) for three of the four molecules (Figure S4C). On the basis of our structural analysis, we could envisage that longer H3 peptides including K4 and phosphorylated marks at S10 and T11 can bind to the peptide-binding site and further stabilize the histone peptide through electrostatic interactions and hydrogen bonds when interacting to R1707 positioned at the beginning of loop 2.

As expected from our peptide binding and structural data, protein sequence alignment of human PHD fingers that specifically recognize unmodified H3 (Figures S3A and S3B) revealed the absence of aromatic residues common to PHD fingers that recognize methylation marks in K4 (Figures S3C and S3D). Within this group, we now can subclassify the PHD fingers of TIP5, BAZ2B, WSTF, and DPF3B as those containing the 3_{10} helix, which prevents the histone tail grapping around the reader domain next to the K4-binding surface. Superposition of TRIM24 (Cα rmsd = 1.48 Å), BHC80 (Cα rmsd = 1.20 Å) with TIP5 crystal structures in complex of H3K4 also demonstrated this feature (Figure S4B). A comparison of available structures of PHD fingers in complex with H3K4me0 highlights how the lysine side chain recognition is essentially formed by acidic residues and interactions through carbonyl backbone groups (Figure S3C). In the case of PHD finger in complex of H3K4me2/3, an aromatic cage preferentially harbors methylated lysines (Figure S3D).

NoRC BRDs Favor Binding to KacXXR Motifs

To gain insight into the recognition of acetylation marks by the NoRC complex, we solved crystal structures of the TIP5 BRD in its free form and in complex with a H4K16acK20ac histone peptide and two cocrystal structures of BAZ2B BRD bound to H3K14ac and H4K8acK12ac peptides (Table 2).

The crystal structure of BAZ2B BRD with acetylated H3K14 revealed sequence-specific interactions of the BRD. The histone peptide was well defined in the electron density map, allowing unambiguous tracing of residues 12 to 19 interacting with the acetyl-lysine binding site (Figure 3H). As expected, the carbonyl group of H3K14ac formed the canonical hydrogen bond with the conserved N2140 as well as a water-mediated hydrogen bond with Y2097 (Figure S5B). The complex was further stabilized by additional hydrogen bonds mediated between the BRD

backbone and H3 peptide amide bonds (Figures 3F and 3G). The typical network of water molecules located at the bottom of the acetyl-lysine binding pocket was conserved (Figure S5B) (Filippakopoulos et al., 2012). Interestingly, two key interactions were observed comprising a hydrophobic stacking interaction between H3P16 with F2139 from the end of helix αB, as well as a sequence-specific interaction involving H3R17 and residues located in the BC loop. The BAZ2B BC loop contains two acidic residues (E2141 and D2142) that formed electrostatic interactions with the side chain of H3R17. These salt bridges are particularly important in determining the binding specificity. An additional hydrogen bond was formed between Nε-H of the arginine side chain and the carbonyl of the E2137 backbone (Figure 3F). We observed only little structural changes comparing the BAZ2B BRD in its free (Protein Data Bank [PDB] accession number 3G0L) and peptide-bound states, suggesting a rigid interaction between the BRD and its targeted acetylated substrate. The most notable difference comparing both structures was the ordering of the side chain of E2141 in the peptide complex. The key residues involved in the H3K14ac recognition (F2139, E2141, and D2142) are conserved in the TIP5 BRD but not in the related BRD proteins BAZ1A and BAZ1B. Sequence alignment (Figure 3B) and thermodynamic binding data (Table 1) suggested a similar molecular recognition of the H3 tail by the TIP5 BRD. Interestingly, moderate to weak binding affinities have been reported for other BRDs to recognize the H3K14ac mark, such as SMARCA2 (K_D = 285 μM by ITC) (Filippakopoulos et al., 2012), SMARCA4 (K_D = 1.2 mM by saturation transfer difference nuclear magnetic resonance [STD-NMR]) (Shen et al., 2007), PCAF (K_D = 128 μM by STD-NMR, K_D = 188 μM by ITC) (Zeng et al., 2008; Filippakopoulos et al., 2012), or PB1 BRD 2 (K_D = 500 μM by STD-NMR) (Charlop-Powers et al., 2010). Our binding studies and molecular characterization with the histone peptide interaction exhibit, unexpectedly, much stronger binding with NoRC BRDs to the acetylated K14 in the low micromolar range (K_D = 10 and 33 μM for BAZ2B and TIP5 BRDs, respectively). A structural comparison with the available NMR structure of the human polybromo BRD 2 in complex with H3K14ac uncovers a diverse peptide-binding mode compared with the BAZ2B complex (Figure S5D). The superposed structures (Cα rmsd = 2.30 Å) exhibit distinct binding cavities. The shallower surface area for the PB1 BRD displays how the peptide extends through the ZA loop, forming weak protein-protein interactions with selectivity driven through H3A15 specific contacts, whereas the deeper hydrophobic binding cavity of BAZ2B accommodates more efficiently the ε-acetyl-lysine 14.

Apart from interaction of TIP5 and BAZ2B with unmodified K4 and acetylated K14ac of H3, we also identified interactions of these two BRDs with acetylated lysines in H4. In order to compare the molecular mechanism of the recognition of these sites, we solved high-resolution crystal structures of TIP5 and BAZ2B BRDs with histone H4 peptides bearing acetylation marks at K8, K12, and K16.

The crystal structures of free-state TIP5 BRD (Figure 3A) as well as in complex with H4K16acK20ac (Figures 3C–3E) were solved at 1.76 and 1.65 Å resolution, respectively. The H4 peptide conformation extended from its N terminus at the ZA loop region to the C terminus at the BC loop region of the BRD. Several residues V1822, V1827, F1872, and V1879 provided a hydrophobic environment to accommodate the hydrophobic segment of the K16ac side chain orienting the N-acetyl group toward the amide nitrogen of N1873. Similar to the BAZ2B complex, further stabilizing contributions were provided by interaction with the conserved water network (Figure S5A). Only few side-chain interactions with BRD residues were involved in the molecular interface, which was mediated mainly through the peptide backbone hydrogen bond interactions with TIP5 residues N1873, F1872, E1874, and R1832. An interesting interaction involved the guanidine group of H4R19 that oriented toward the BC loop, forming a hydrogen bond between its Nε-H and the carbonyl of Q1870, and a salt bridge with the two acidic residues E1874 and D1875 (Figure 3D). These features demonstrate a conservation of BRD-histone interactions between TIP5 and BAZ2B in this region. Analyses of our peptide arrays, ITC data, and crystal structure suggests that much of the recognition specificity for H4K16ac by the TIP5 BRD in vitro is due to the intricate network of interactions formed by the R19 side chain. ITC determined an affinity of 23 μM for H4K16ac (G14-A15-K16ac-R17-H18-R19-K20-V21-L22), whereas binding affinity decreased 2-fold for the H4K12acK16ac (G9-L10-G11-K12ac-G13-G14-A15-K16ac-R17). We compared the cocrystal structure of TIP5 BRD with the NURF (nucleosome remodeling factor) subunit BPTF (BRD and PHD finger-containing transcription factor), which was also crystallized with H4K16ac (Cα rmsd = 0.61 Å) (Figure S5C). We observed a similar conformation of R19 folding toward the BC loop. However, the NoRC BRDs are characterized by a more negative electrostatic potential in this region, favoring interaction with positively charged residues such as H4R19, thus offering a rationale for the weaker interaction of this peptide with BPTF. We determined K_D values of 23 μM for TIP5 and 46 μM for BAZ2B in comparison with a K_D of 99 μM for the BPTF BRD. Indeed, a second crystal form of a complex of BPTF with H4K16ac showed an inverse peptide orientation most likely reflecting the lower specificity for this mark (Ruthenburg et al., 2011).

In contrast to H3K14ac and H4K16ac, the diacetylated peptide H4(6–13)K8acK12ac simultaneously bound two BAZ2B BRD molecules in the complex crystal structure that was solved at 1.6 Å resolution (Figures 3I–3K). Interestingly, in this dual-recognition binding mode, the segment H4(6–10) containing the K8ac mark targets one BRD molecule, whereas the H4(10–13) is recognized by a second BRD module. The linking residue L10 may favor the crystallographic packing in its well-accommodated hydrophobic cage formed by F2139 by each BRD. ITC measurements also confirmed a 2:1 stoichiometry of protein to peptide, hence two N-acetyl lysine binding sites for a single histone tail with two acetylation marks. Although this complex is unlikely to be physiological, structural data suggest that BAZ2B BRD recognize only single lysine acetylation marks per binding site.

Regarding surface properties around the acetyl-lysine cavity, all three cocrystal structures show a slightly negatively charged electrostatic potential (Figures 3C, 3F, and 3I), which is in agreement with recognition of largely basic peptides. Interestingly, both TIP5 and BAZ2B BRD contain the aromatic “WPF shelf” at the ZA loop shared with BRD families such as the BET family. However, in the BAZ BRDs, these three aromatic residues adopt a closer conformation compared with the more open form of

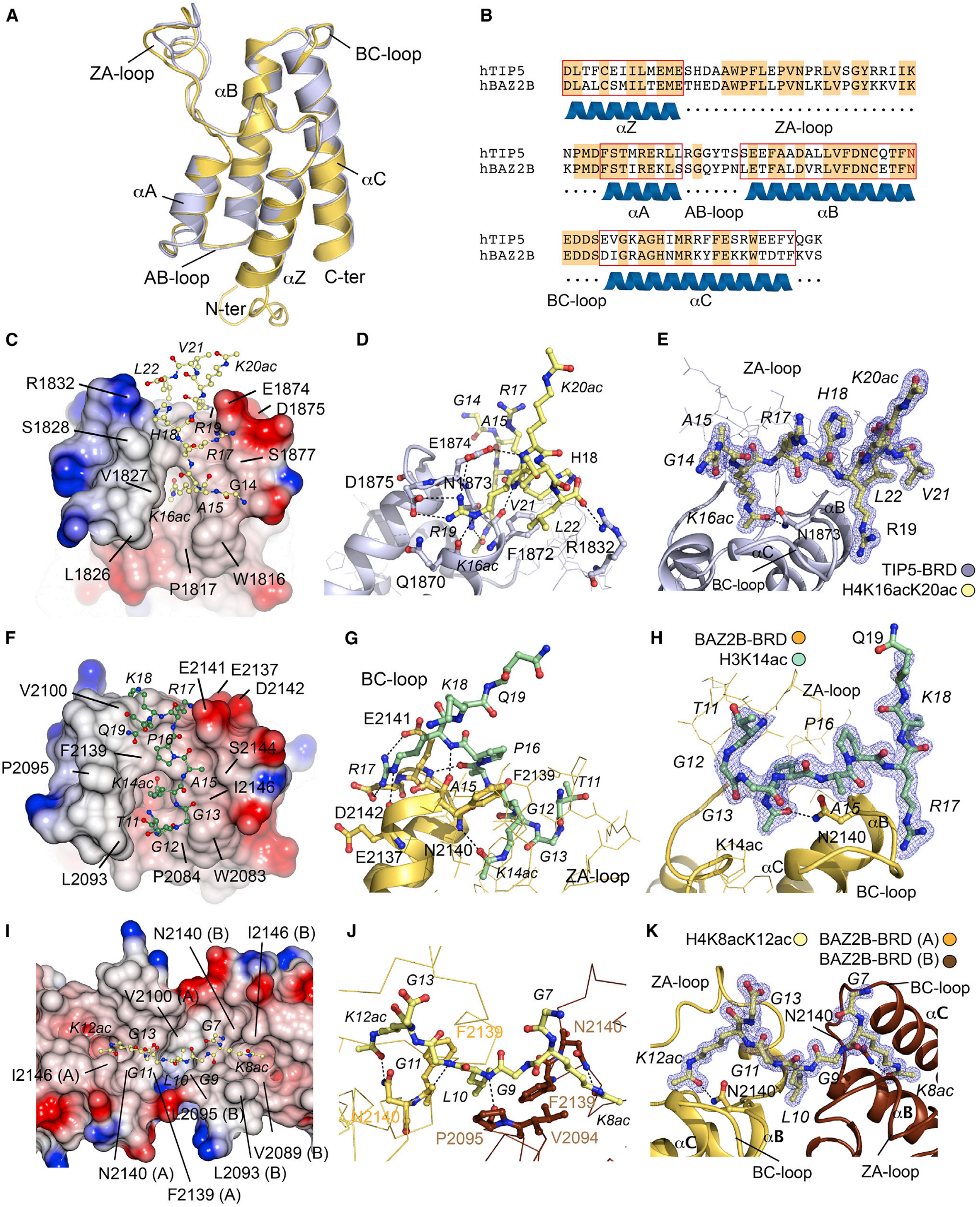

Figure 3. Structures of TIP5 and BAZ2B BRDs in the Free State and in Complex with H3 and H4 Peptides

(A) Crystal structures superposition on ribbon representation of novel TIP5 (PDB accession number 4LZ2) and BAZ2B (PDB accession number 3G0L) BRDs on Cα positions (rmsd = 0.488).

(legend continued on next page)

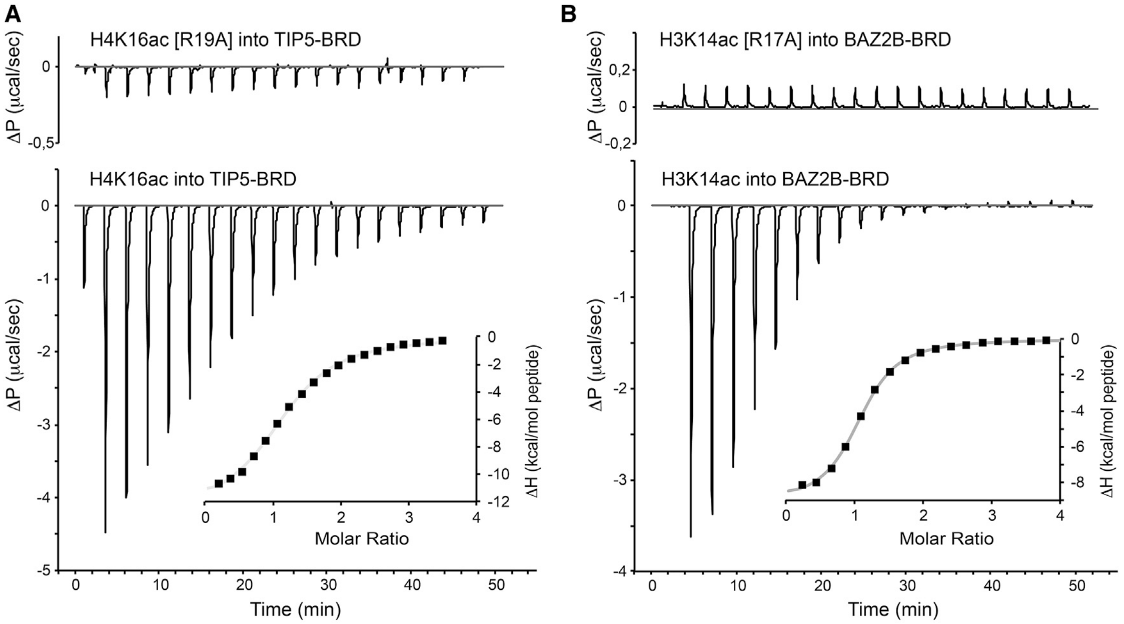

C

Peptides	Protein	K_D (μM)	N	ΔH (kcal/mol)	TΔS (kcal/mol)	ΔG (kcal/mol)
H4(14-22)K16ac	TIP5 BRD	23.69 ± 0.62	1.186 ± 0.015	-12.740 ± 0.226	-6.533	-6.206
H4(14-22)K16ac [R19A]	TIP5 BRD	no binding				
H3(9-19)K14ac	BAZ2B BRD	10.35 ± 0.20	1.050 ± 0.008	-9.147 ± 0.095	-2.455	-6.692
H3(9-19)K14ac [R17A]	BAZ2B BRD	no binding				

Figure 4. ITC-Based Binding Curves of Individual BRDs with Histone Peptides and Further Analysis on the Specificity of the Recognition Sequence Kac-X-X-R

(A) H4K16ac (Gly-Ala-Kac-Arg-His-Arg-Lys-Val-Leu) and the specific mutated H4K16ac (R19A) (Gly-Ala-Kac-Arg-His-**Ala**-Lys-Val-Leu) peptides are titrated separately into a solution of TIP5 BRD. The single mutation on the residue R19 abolishes the binding to the BRD.

(B) A similar analysis has been performed for BAZ2B and its binding specificity to H3R17. Wild-type H3K14ac (Lys-Ser-Thr-Gly-Gly-Kac-Ala-Pro-Arg-Gln) and mutated H3K14ac (R17A) (Lys-Ser-Thr-Gly-Gly-Kac-Ala-Pro-**Ala**-Gln) are titrated into a solution of BAZ2B BRD. No binding is observed for the mutated R17A peptide.

(C) Thermodynamic parameters summary and comparison of wild-type and mutated peptides at the specific arginine position. The same sequence length peptides were purchased with their corresponding arginine mutation.

BET BRDs that creates a wider binding site sufficient to accommodate two acetyl-lysine side chains in BET (Morinière et al., 2009; Filippakopoulos et al., 2012).

Our studies demonstrate how TIP5 and BAZ2B BRDs specifically recognize the K14ac-X-X-R17 motif, thereby discriminating against other acetylation marks in histone H3. The specificity of these reader domains is more variable in the case of histone H4 peptides, but we also demonstrated that histone H4 recognition is also favored in the case of the K16ac-X-X-R19 motif.

In order to further confirm this sequence motif preference, mutated H4 histone peptide at position R19 to A19 and H3 histone peptide at position R17 to A17 were used to measure binding affinities against TIP5 and BAZ2B BRDs, respectively. The results obtained (Figure 4) show no binding toward the peptides containing the arginine mutation, confirming the role of this specific arginine position for the histone recognition. Furthermore, methylation at the R residue of the K14ac-X-X-R17 motif significantly decreased histone peptide binding in BLI (Figure S2A), reinforcing the role of unmodified Arg side chain

(B) Structure-based sequence alignment of single BRDs from the human TIP5 and BAZ2B. The conserved asparagine involved in the histone recognition is highlighted in red.

(C, F, and I) Surface representations of the peptide-bound structures of single BRDs. (C) Corresponds to TIP5-BRD in complex with H4K16acK20ac (GA**Kac**RHRKacVL), with the acetylation recognized by the conserved asparagine highlighted in bold. (F) BAZ2B-BRD in complex with H3K14ac (TGG**Kac**APRKQ). (I) BAZ2B-BRD in complex with H4K8acK12ac (GG**Kac**GLG**Kac**G). Electrostatic surface potentials are shown between +5 V (blue) and −5 V (red).

(D, G, and J) Detailed interactions between BRD of TIP5 with H4K16acK20ac and BRD of BAZ2B with H3K14ac and H4K8acK12ac.

(E, H, and K) View of the peptide-binding pocket. Simulated-annealing 2Fo-Fc maps showing the electron density for the H3 and H4 peptides. Maps were calculated at 1.65, 1.99, and 1.60 Å resolution and contoured between 1.2 and 1.5 σ above the mean.

See also Figure S5.

in the molecular recognition. Taken together, our data in the context of isolated histone peptides strongly suggest a general recognition specificity of KacXXR motif for this subclass of BRDs.

The NoRC PHD and BRD Act as *trans* Histone Recognition Modules

PHD/BRD reader motifs are evolutionary-conserved peptide-binding modules. Previous crystal structures revealed either a tightly connected dual-reader unit (Tsai et al., 2010; Xi et al., 2011; Plotnikov et al., 2014) or a rigidly linked dual-domain structure (Ruthenburg et al., 2011). We therefore investigated the role of the two adjacent reader domains of TIP5 and BAZ2B and asked whether they perform simultaneous engagement to the same substrate or to two different substrates. Our attempts at crystallizing constructs harboring both reader domains have failed to date. Likely the unstructured nature of the linker amino acid sequence between the PHD finger and the BRD prevented our efforts in crystallizing the full-length tandem proteins. Indeed, analysis of the sequences linking both domains suggests that this region is intrinsically disordered using different bioinformatics analysis tools (IUPred and DISpro) that predict protein disorder from amino acid sequences (Dosztányi et al., 2005; Linding et al., 2003; Cheng et al., 2005). The output consistently indicated a high likelihood of a disordered or flexible linker between the PHD finger domain and the BRD (approximately 65 and 70 residues junction between reader domains of TIP5 and BAZ2B) (Figure S6A). Size exclusion chromatographic analysis of mixtures of individual PHD finger and BRD of each protein showed single elution peaks, suggesting no intermolecular interaction between reader domains (data not shown). However, the ability of a potential spring motion of the middle junction between domains leading to a *cis* engagement to H3 on the basis of recognition of unmodified K4 and acetylated K14 was not clear with our thermodynamic data, in which there is no evidence of an enhancement on the binding affinity (K_D values of 1.9 μM for TIP5 and 2.3 μM BAZ2B tandem domains) (Table 1).

In order to examine the plasticity of this junction between reader domains in solution, we further analyzed tandem TIP5 and BAZ2B proteins (PHD-BRD) using SAXS. The scattering data were collected from free tandem domains and, for BAZ2B, also in complex with the histone H3 peptide with the K14 acetylation mark H3(1–18)K14ac (Table S1). The experimental molecular mass (MM) of both proteins (28 ± 5 kDa for TIP5 and 33 ± 5 kDa for BAZ2B) points to monomeric state of the two PHD-BRD constructs in solution. The Kratky plots (Figure S6B) for the tandems in the free state do not display a bell shape, expected for compact particles, but instead indicate an extended shape and potential flexibility. Comparing the two curves in Figure S6B, TIP5 tandem shows somewhat higher compactness, whereas BAZ2B follows a pattern closer to that of flexible proteins. These qualitative conclusions are corroborated by the overall parameters of the two constructs, radius of gyration (R_g) and maximum diameter (D_{max}) (R_g = 31 ± 1 Å, D_{max} = 100 ± 10 Å for TIP5; R_g = 42 ± 1 Å, D_{max} = 145 ± 10 Å for BAZ2B). These parameters are indicative of extended structures, whereby TIP5 is more compact than BAZ2B. The distance distribution functions $p(r)$ displayed skewed shapes characteristic for elongated particles (Figure S6C). To characterize the potential protein mobility, an ensemble optimization method (EOM; Bernadó et al., 2007) was used, which selects an ensemble of conformers fitting the experimental data from a large pool of generated models with random linker conformations. The R_g and D_{max} distribution analyses of TIP5 from the EOM-selected ensemble (Figures 5C–5E) indicated that the ensemble is more compact than the random pool, suggesting a limited flexibility of the linker between reader domains. In contrast, the EOM results on the collected SAXS data of BAZ2B (Figures 5F–5H), showed broad R_g and D_{max} distributions, which are more extended in comparison with the pool, thereby suggesting high flexibility on the linker. The combined data strongly proposed an extended and flexible linker conformation for BAZ2B and a rather more rigid and compact arrangement for TIP5. The limited flexibility of TIP5 allowed us to reconstruct its low-resolution shape ab initio and also to build a hybrid model of the construct in which the linker is represented by a chain of dummy residues. The models presented in Figures 5A and 5B fit well the experimental data and display similar overall shapes revealing the probable conformation that TIP5 can adopt in solution.

The SAXS data were also measured for the complex of the tandem domains with the H3 histone peptide. However, only interpretable data were obtained for the complex of the BAZ2B tandem. The H3 bound form provides an MM of 45 ± 5 kDa and displays a distance distribution profile $p(r)$ similar to that of the free protein (Figure S6C) but with an increased maximum size (D_{max} = 160 ± 10 Å). R_g also increases for the bound compared with the free state (Table S1). These results clearly indicate that the peptide interacts with the individual reader domains independently, as simultaneous binding to both of them would have decreased, not increased, R_g and D_{max}. The intrinsic dynamic motion of the junction between domains would not favor interactions of both domains in *cis* in the case of the extended H3K14ac (residues 1–18). In summary, the SAXS data and analysis have provided us with a low-resolution model of the overall structure of TIP5 PHD/BRD revealing well-separated individual domains. Besides, for BAZ2B, our biophysical and low-resolution structural data support a flexible linker model between the PHD and BRD, but we had no evidence of *cis* interaction to the same histone peptide but most favorable recognition in a *trans* manner with two histones tails.

Conclusions

In this study, we used biochemical screening, structural, and biophysical approaches in vitro to investigate the molecular mechanism of TIP5 and BAZ2B histone recognition.

The crystal structures of free and bound TIP5 BRD with H4K16ac demonstrated the selectivity of the reader domain to this epigenetic mark, as previously suggested by Zhou and Grummt (2005). Interestingly, we found that TIP5 and BAZ2B BRDs also have considerable affinity for acetylated sequences in histone H3. By far, the most favorable mark in H3 was K14ac. Cocrystal structures with H4K16ac and H3K14ac revealed single acetylation recognition to the Kac-X-X-R sequence motif, where the second X is preferably an hydrophobic or aromatic residue. Also, polar interactions with the BC loop of the BRD are conserved. Such substrate specificity of the TIP5 and BAZ2B BRDs for H3 and H4 marks reinforces the argument

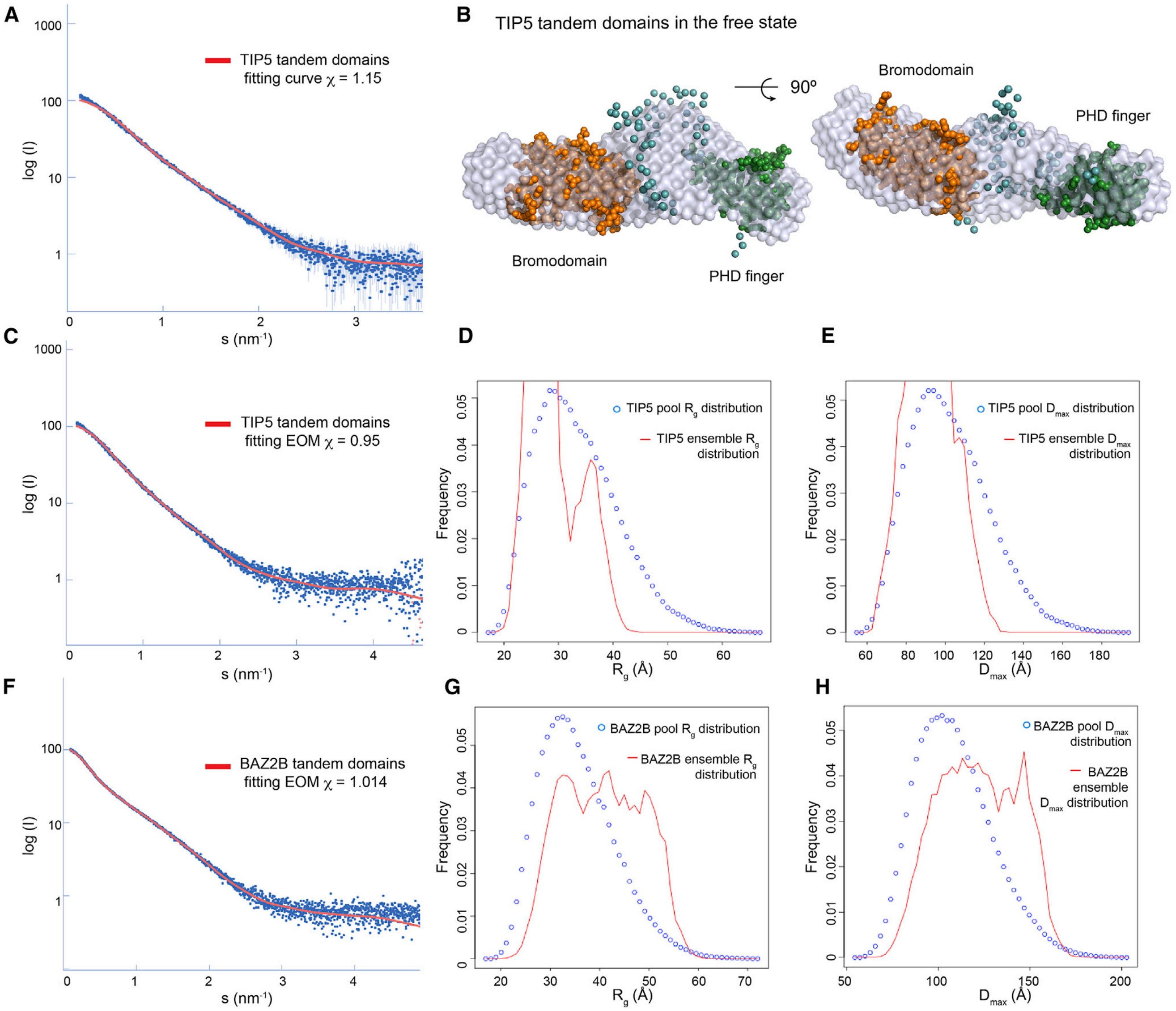

Figure 5. SAXS Analyses Are Shown for Tandem TIP5 PHD-BRD and BAZ2B PHD-BRD in the Free State

(A) The experimental SAXS profile (log intensities as a function of the momentum transfer) for free TIP5 (blue spotty curve) and the fitting curve (red) using the corresponding crystallographic structures from the BRD and PHD finger.

(B) The ab initio calculated SAXS model is superimposed on the individual crystal structures linked with dummy atoms (cyan spheres) representing the linker between modules for TIP5 (BRD, orange spheres; PHD finger, green spheres).

(C) The experimental SAXS profile for TIP5 with the fitting curve on the basis of the EOM distribution calculations.

(D) R_g distributions from EOM-selected ensemble (red) for TIP5 tandem and that corresponding to the pool (blue).

(E) D_{max} distribution from the pool (blue) and optimized ensemble (red) for TIP5.

(F) Same as (C) but with BAZ2B.

(G) Same as (D) but with BAZ2B.

(H) Same as (E) but with BAZ2B.

See also Figure S6 and Table S1.

that a major function of this domain is to facilitate the coupling between histone acetylation and the process of nucleosome remodeling mediated by the ATPase subunit.

Our work further provides the structural elucidation of two previously uncharacterized PHD fingers, which are adjacent to the BRDs from the C terminus of TIP5 and BAZ2B. Screening with histone peptide PTM marks revealed the specificity of the PHD fingers for unmodified or singly-methylated H3K4. Additional high-resolution crystal structure of the unmodified peptide bound deciphers how this peptide accommodates well in the PHD zinc finger shallow surface. However, an important outcome of our analysis established that unmodified H3K4 and acetylated H3K14 are not recognized in a combinatorial manner by PHD-BRD tandem modules of BAZ2B in solution. In summary,

complex structures analysis established that NoRC readout of histone PTMs potentially depend on an unmodified state of H3K4 and acetylation states of preferred positions on H3K14 and H4K16.

The disclosure of our structural work on the BAZ2 BRD with histone substrate will aid in the rational design for targeting these reader domains with small molecules. Additionally, structural elucidation of the BAZ2 PHD zinc finger domains alone and in complex with the histone substrate provide the structural basis to consider targeting this other reader domain as well. Recently, the BAZ2 proteins have emerged as new epigenetic drug targets because of their involvement in human diseases as evidenced by microRNA expression profiles in prostate cancer progression (Leite et al., 2011) or upregulated BAZ2A gene in patients with chronic lymphocytic leukemia (Hanlon et al., 2009). Therefore, a rising pharmacological interest on targeting BAZ2 reader domains has evolved with the discovery and optimization of micromolar fragment-like molecules (Ferguson et al., 2013) and selective nanomolar compounds GSK2801 and BAZ2-ICR (see the Structural Genomics Consortium [SGC] website at http://www.thesgc.org) targeting the BAZ2 BRD.

SIGNIFICANCE

Molecular recognition of the PTM H4K16ac by the TIP5 PHD-BRD tandem facilitates the recruitment of the NoRC complex to chromatin and initiates the cascade of events that trigger gene silencing. Using an in vitro PTM histone biophysical screening approach, we demonstrate more extended histone recognition profile of the PHD finger and BRD to H3 and H4. Our results allow us to classify the TIP5 and BAZ2B PHD fingers as unmodified H3K4 recognition subclass with single-digit μM affinities. Structural details of this interaction confirm the absence of an aromatic cage and the presence of negatively charged residues that form hydrogen bonds with the K4 side chain. Our work has also provided molecular insights into the BRD function of the NoRC complex. Crystal structure complexes with histone tails explain how TIP5 and BAZ2B BRDs recognize the specific pattern of H3 and H4 histones: Kac-X-X-R. Additionally, our structural studies of the tandem PHD-BRD in solution suggest an extended conformation with potential independent histone recognition. The precise understanding of the histone recognition of the NoRC/BAZ2B reader domains will aid in targeting these reader domains in ligand discovery campaigns.

EXPERIMENTAL PROCEDURES

Cloning, Protein Expression, and Purification

Analytical details for construct design, protein overexpression, purification, and characterization are given in Supplemental Experimental Procedures.

BLI of Modified Histone Peptide Arrays

Biotinylated histone sets were purchased from AltaBioscience. Libraries contain different combination of PTMs of histones N-terminal tails (Figures S2A and S2B). Peptides were immobilized on two trays of Super Streptavidin (SSA) Biosensors (FórteBio). All binding experiments were conducted at 25°C in buffer 20 mM Tris·HCl (pH 8), 200 mM NaCl, 2 mM dithiothreitol, using an Octet[Red] 384 instrument (FórteBio). The final nontagged protein concentration of all samples was 20 μM, dispensed in a volume of 100 μl per well. Assays were performed in black, solid, 384-well, flat-bottom plates harboring the analytes and agitated to 1,000 rpm. Common cycles steps for analysis included 120 s of biosensor baseline equilibration step, associations in wells containing the free label protein for 240 s, and dissociations in buffer wells for 240 s. Reference subtraction was performed with the FórteBio data analysis software to subtract the effect of baseline drift and the effect of nonspecific binding to biosensor tips without immobilized peptides.

ITC Measurements

All calorimetric experiments were performed on an ITC200 or VP-ITC microcalorimeters (MicroCal). Protein solutions were buffer exchanged by gel filtration into buffer (20 mM HEPES [pH 8], 150 mM NaCl, 0.5 mM Tris [2-carboxyethyl] phosphine [TCEP]), and lyophilized histone peptides were dissolved in the same buffer. All measurements were carried out at 293 or 298 K. Protein solutions (60–120 μM) in the calorimetric cell were titrated with the peptide solutions (800–1,500 μM) in the syringe. The data were analyzed with the MicroCal ORIGIN software package using, in most cases, a single binding site model. The first data point was excluded from the analysis. Thermodynamic parameters and binding constants are given in Table 1.

SAXS Experiments

Synchrotron radiation X-ray scattering data were collected at two different beamlines for BAZ2B apo form and in complex with H314ac (1–18) and TIP5 apo form and in complex with H3K14ac (1–18). For BAZ2B, four different concentrations in the range 1.1 to 10.5 mg/ml in 20 mM Tris (pH 8), 500 mM NaCl, 2 mM DDT, and 10 μM $ZnCl_2$ were collected on the X33 camera of the European Molecular Biology Laboratory (EMBL) storage ring DORIS III (Deutsches Elektronen-Synchrotron [DESY]). Data were collected using a photon-counting Pilatus 1M detector at a sample-detector distance of 2.7 m and a wavelength of λ = 1.5 Å; the range of momentum transfer $0.09 < s < 6.0$ nm^{-1} was covered ($s = 4\pi \sin\theta/\lambda$, where θ is the scattering angle). To monitor radiation damage, eight successive 15 s exposures were compared, and no significant changes were observed. For TIP5, four different concentrations in the range 1.1 to 10.5 mg/ml in 20 mM Tris (pH 8), 500 mM NaCl, 2 mM DDT, and 10 μM $ZnCl_2$ were collected on the P12 camera of the EMBL storage ring PETRA III (DESY). Data were collected using a photon-counting Pilatus 2M detector at a sample-detector distance of 3.1 m and a wavelength of λ = 1.24 Å; the range of momentum transfer $0.07 < s < 4.6$ nm^{-1} was covered. To monitor radiation damage, 20 successive 1 s exposures were compared, and no significant changes were observed.

In both cases, the data were normalized to the intensity of the transmitted beam and radially averaged; the scattering of the buffer was subtracted, and the difference curves were scaled for protein concentration. The low-angle data collected were obtained from single-concentration scattering curves, except for TIP5, for which two different concentrations were merged. The forward scattering $I(0)$, the radius of gyration R_g, the pair distribution of the particle $p(r)$, and the maximum dimension D_{max} were computed using the automated SAXS data analysis pipeline (Franke et al., 2012). The MM of BAZ2B and TIP5 was evaluated by comparison of the forward scattering with that from a reference solution of BSA (MM = 66 kDa). DAMMIF (Franke and Svergun, 2009) was used to compute the ab initio models, BUNCH (Petoukhov and Svergun, 2005) was used to model TIP5, and EOM (Bernadó et al., 2007) was used to assess the flexibility of both BAZ2B and TIP5.

Crystallization

Highly purified proteins were crystallized at either 4°C or 20°C using vapor diffusion methods. Details on the protein crystallization in the free state and in complex with peptides are provided in Supplemental Experimental Procedures.

Data Collection and Structure Determination

Zinc single-wavelength anomalous diffraction data were collected at peak wavelength for both crystals of TIP5 and BAZ2B PHD Zinc fingers at the Diamond Light Source (beamlines I02 and I24) and processed to 1.70 and 1.60 Å, respectively, using XDS (Kabsch 2010) and SCALA (Evans 2006). The zinc positions in both proteins were determined using SHELXC/D (Sheldrick 2010), the SAD data enabled to find eight zinc sites per asymmetric unit in TIP5 and four zinc sites per asymmetric unit in BAZ2B (two per monomer for both cases). Phases and density modification were calculated using the program SHELXE (Sheldrick 2010). A total of 50 cycles of automated protein

chain tracing starting from experimental phases were computed using ARP/wARP (Langer et al., 2008), obtaining 209 of 232 residues in the ASU for BAZ2A-PHD and 109 of 116 for BAZ2B-PHD. Further manual model building was performed using Coot (Emsley and Cowtan, 2004) alternated with crystallographic refinement with REFMAC5 (Murshudov et al., 1997) within the CCP4 suite until the final model was obtained. Crystal structure of the TIP5-PHD zinc finger with unmodified H3K4 peptide bound was elucidated by molecular replacement with the apo TIP5 PHD finger structure using PHASER (McCoy et al., 2005). Omit map showed clear electron density to fit the 5-mer H3K4 peptide. Further manual building and refinement were carried out using Coot and REFMAC5.

A complete data set was collected for the TIP5 BRD apo crystals at the Diamond Light Source (beamline I04) and processed to 1.78 Å using XDS and AIMLESS. Crystal structure of the free-state BRD was solved by molecular replacement using PHASER using an ensemble of different BRDs as the searching templates (PDB accession numbers: PCAF, 3GG3; BPTF, 3UV2; BAZ2B, 3G0L; BRD2[1], 1X0J; BRD2[2], 2DVV; BRD3[2], 2OO1; BRD4[1], 2OSS; BRD4[2], 2OUO; GCN5, 3D7C; ATAD2, 3DAI; CREBBP, 3DWY; TAF1L[2], 3HMH). The unique and initial solution was improved in a total of 50 cycles of automated protein chain tracing starting from the existing model, computed using ARP/wARP. Further manual building (Coot) and refinement against maximum likelihood target were performed using REFMAC5.

Data sets were collected for the cocrystals of histone peptides with TIP5 and BAZ2B BRD at Diamond Light Source (beamline I04) and processed to 1.65, 1.99, and 1.60 Å (TIP5-H4K16acK20ac, BAZ2B-H3K14ac, and BAZ2B-H4K8acK12ac, respectively) using XDS and AIMLESS. Three crystal structures were solved by molecular replacement using PHASER with the same ensemble of BRDs mentioned above as the searching templates. All three coordinate solutions were further manually built and refined using Coot and REFMAC5.

All model validations were carried out using MolProbity (Chen et al., 2010). All data collections and refinement statistics are shown in Table 2. Figures were generated using PyMOL, CCP4mg, and ICM-Molsoft molecular viewer.

ACCESSION NUMBERS

The atomic coordinates and structure factors of human TIP5 and BAZ2B PHD zinc fingers have been deposited in the PDB with accession numbers 4QF2 and 4QF3. Complex of TIP5 PHD finger with H3 peptide was deposited with PDB accession number 4Q6F. Human TIP5-BRD in the free state was deposited with PDB accession number 4LZ2. Complex structures of TIP5-BRD with H4 histone and BAZ2B-BRD with H3 and H4 histones were deposited with PDB accession numbers 4QBM, 4QC1, and 4QC3, respectively. SAXS experimental data and analysis is deposited in Small Angle Scattering Biological Data Bank (http://www.sasbdb.org) with accession numbers SASDA46 (TIP5 free state), SASDA56 (BAZ2B free state), and SASDA66 (BAZ2B in complex of histone H3(1-18)K14ac peptide).

SUPPLEMENTAL INFORMATION

Supplemental Information includes Supplemental Experimental Procedures, six figures, and one table and can be found with this article online at http://dx.doi.org/10.1016/j.str.2014.10.017.

ACKNOWLEDGMENTS

This work was supported by the UK Biotechnology and Biological Sciences Research Council (grants BB/G023123/1 David Phillips Fellowship to A.C. and BB/J001201/1 to A.C.) and a Federation of European Biochemical Societies short-term fellowship (04-11-12-10 to C.T.). We are grateful to Dr. Dimitri Y. Chirgadze of the Crystallographic X-Ray Facility at the Department of Biochemistry, University of Cambridge, and to the technical support at Diamond Light Source Synchrotron Facilities. We acknowledge support from the European Commission FP7 Programme under BioStruct-X (grant agreement 283570) for SAXS data collection at the EMBL (DESY). The SGC is a registered charity (No. 1097737) that receives funds from AbbVie, Bayer, Boehringer Ingelheim, the Canada Foundation for Innovation, the Canadian Institutes for Health Research, Genome Canada, GlaxoSmithKline, Janssen, Lilly Canada, the Novartis Research Foundation, the Ontario Ministry of Economic Development and Innovation, Pfizer, Takeda, and the Wellcome Trust (092809/Z/10/Z). E.V. is supported by a European Commission FP7 Marie Curie grant IDPbyNMR (contract 264257). P.F. is supported by a Welcome Trust Career Development Fellowship (095751/Z/11/Z).

Received: July 21, 2014
Revised: September 24, 2014
Accepted: October 13, 2014
Published: December 18, 2014

REFERENCES

Bernadó, P., Mylonas, E., Petoukhov, M.V., Blackledge, M., and Svergun, D.I. (2007). Structural characterization of flexible proteins using small-angle X-ray scattering. J. Am. Chem. Soc. *129*, 5656–5664.

Charlop-Powers, Z., Zeng, L., Zhang, Q., and Zhou, M.M. (2010). Structural insights into selective histone H3 recognition by the human Polybromo bromodomain 2. Cell Res. *20*, 529–538.

Chen, V.B., Arendall, W.B., 3rd, Headd, J.J., Keedy, D.A., Immormino, R.M., Kapral, G.J., Murray, L.W., Richardson, J.S., and Richardson, D.C. (2010). MolProbity: all-atom structure validation for macromolecular crystallography. Acta Crystallogr. D Biol. Crystallogr. *66*, 12–21.

Cheng, J., Sweredoski, M., and Baldi, P. (2005). Accurate prediction of protein disordered regions by mining protein structure data. Data Min. Knowl. Discov. *11*, 213–222.

Delvecchio, M., Gaucher, J., Aguilar-Gurrieri, C., Ortega, E., and Panne, D. (2013). Structure of the p300 catalytic core and implications for chromatin targeting and HAT regulation. Nat. Struct. Mol. Biol. *20*, 1040–1046.

Dosztányi, Z., Csizmók, V., Tompa, P., and Simon, I. (2005). IUPred: web server for the prediction of intrinsically unstructured regions of proteins based on estimated energy content. Bioinformatics *21*, 3433–3434.

Eberharter, A., Vetter, I., Ferreira, R., and Becker, P.B. (2004). ACF1 improves the effectiveness of nucleosome mobilization by ISWI through PHD-histone contacts. EMBO J. *23*, 4029–4039.

Emsley, P., and Cowtan, K. (2004). Coot: model-building tools for molecular graphics. Acta Crystallogr. D Biol. Crystallogr. *60*, 2126–2132.

Evans, P. (2006). Scaling and assessment of data quality. Acta Crystallogr. D Biol. Crystallogr. *62*, 72–82.

Ferguson, F.M., Fedorov, O., Chaikuad, A., Philpott, M., Muniz, J.R., Felletar, I., von Delft, F., Heightman, T., Knapp, S., Abell, C., and Ciulli, A. (2013). Targeting low-druggability bromodomains: fragment based screening and inhibitor design against the BAZ2B bromodomain. J. Med. Chem. *56*, 10183–10187.

Filippakopoulos, P., Picaud, S., Mangos, M., Keates, T., Lambert, J.P., Barsyte-Lovejoy, D., Felletar, I., Volkmer, R., Müller, S., Pawson, T., et al. (2012). Histone recognition and large-scale structural analysis of the human bromodomain family. Cell *149*, 214–231.

Franke, D., and Svergun, D.I. (2009). DAMMIF, a program for rapid ab-initio shape determination in small angle scattering. J. Appl. Cryst. *42*, 342–346.

Franke, D., Kikhney, A.G., and Svergun, D.I. (2012). Automated acquisition and analysis of small angle X-ray scattering data. Nucl. Instrum. Methods *689*, 52–59.

Grummt, I. (2007). Different epigenetic layers engage in complex crosstalk to define the epigenetic state of mammalian rRNA genes. Hum. Mol. Genet. *16*, R21–R27.

Hanlon, K., Rudin, C.E., and Harries, L.W. (2009). Investigating the targets of MIR-15a and MIR-16-1 in patients with chronic lymphocytic leukemia (CLL). PLoS ONE *4*, e7169.

Kabsch, W. (2010). XDS. Acta Crystallogr. D Biol. Crystallogr. *66*, 125–132.

Langer, G., Cohen, S.X., Lamzin, V.S., and Perrakis, A. (2008). Automated macromolecular model building for X-ray crystallography using ARP/wARP version 7. Nat. Protoc. *3*, 1171–1179.

Leite, K.R., Sousa-Canavez, J.M., Reis, S.T., Tomiyama, A.H., Camara-Lopes, L.H., Sañudo, A., Antunes, A.A., and Srougi, M. (2011). Change in expression of miR-let7c, miR-100, and miR-218 from high grade localized prostate cancer to metastasis. Urol. Oncol. *29*, 265–269.

Linding, R., Jensen, L.J., Diella, F., Bork, P., Gibson, T.J., and Russell, R.B. (2003). Protein disorder prediction: implications for structural proteomics. Structure *11*, 1453–1459.

McCoy, A.J., Grosse-Kunstleve, R.W., Storoni, L.C., and Read, R.J. (2005). Likelihood-enhanced fast translation functions. Acta Crystallogr. D Biol. Crystallogr. *61*, 458–464.

McNicholas, S., Potterton, E., Wilson, K.S., and Noble, M.E. (2011). Presenting your structures: the CCP4mg molecular-graphics software. Acta Crystallogr. D Biol. Crystallogr. *67*, 386–394.

Morinière, J., Rousseaux, S., Steuerwald, U., Soler-López, M., Curtet, S., Vitte, A.L., Govin, J., Gaucher, J., Sadoul, K., Hart, D.J., et al. (2009). Cooperative binding of two acetylation marks on a histone tail by a single bromodomain. Nature *461*, 664–668.

Murshudov, G.N., Vagin, A.A., and Dodson, E.J. (1997). Refinement of macromolecular structures by the maximum-likelihood method. Acta Crystallogr. D Biol. Crystallogr. *53*, 240–255.

Petoukhov, M.V., and Svergun, D.I. (2005). Global rigid body modeling of macromolecular complexes against small-angle scattering data. Biophys. J. *89*, 1237–1250.

Philpott, M., Yang, J., Tumber, T., Fedorov, O., Uttarkar, S., Filippakopoulos, P., Picaud, S., Keates, T., Felletar, I., Ciulli, A., et al. (2011). Bromodomain-peptide displacement assays for interactome mapping and inhibitor discovery. Mol. Biosyst. *7*, 2899–2908.

Plotnikov, A.N., Yang, S., Zhou, T.J., Rusinova, E., Frasca, A., and Zhou, M.M. (2014). Structural insights into acetylated-histone H4 recognition by the bromodomain-PHD finger module of human transcriptional coactivator CBP. Structure *22*, 353–360.

Ruthenburg, A.J., Li, H., Milne, T.A., Dewell, S., McGinty, R.K., Yuen, M., Ueberheide, B., Dou, Y., Muir, T.W., Patel, D.J., and Allis, C.D. (2011). Recognition of a mononucleosomal histone modification pattern by BPTF via multivalent interactions. Cell *145*, 692–706.

Sanchez, R., and Zhou, M.M. (2011). The PHD finger: a versatile epigenome reader. Trends Biochem. Sci. *36*, 364–372.

Sheldrick, G.M. (2010). Experimental phasing with *SHELXC/D/E*: combining chain tracing with density modification. Acta Crystallogr. D Biol. Crystallogr. *66*, 479–485.

Shen, W., Xu, C., Huang, W., Zhang, J., Carlson, J.E., Tu, X., Wu, J., and Shi, Y. (2007). Solution structure of human Brg1 bromodomain and its specific binding to acetylated histone tails. Biochemistry *46*, 2100–2110.

Tsai, W.W., Wang, Z., Yiu, T.T., Akdemir, K.C., Xia, W., Winter, S., Tsai, C.Y., Shi, X., Schwarzer, D., Plunkett, W., et al. (2010). TRIM24 links a non-canonical histone signature to breast cancer. Nature *468*, 927–932.

Wu, J.I., Lessard, J., and Crabtree, G.R. (2009). Understanding the words of chromatin regulation. Cell *136*, 200–206.

Xi, Q., Wang, Z., Zaromytidou, A.I., Zhang, X.H., Chow-Tsang, L.F., Liu, J.X., Kim, H., Barlas, A., Manova-Todorova, K., Kaartinen, V., et al. (2011). A poised chromatin platform for TGF-β access to master regulators. Cell *147*, 1511–1524.

Zeng, L., Zhang, Q., Gerona-Navarro, G., Moshkina, N., and Zhou, M.M. (2008). Structural basis of site-specific histone recognition by the bromodomains of human coactivators PCAF and CBP/p300. Structure *16*, 643–652.

Zhou, Y., and Grummt, I. (2005). The PHD finger/bromodomain of NoRC interacts with acetylated histone H4K16 and is sufficient for rDNA silencing. Curr. Biol. *15*, 1434–1438.

Zhou, Y., Schmitz, K.M., Mayer, C., Yuan, X., Akhtar, A., and Grummt, I. (2009). Reversible acetylation of the chromatin remodelling complex NoRC is required for non-coding RNA-dependent silencing. Nat. Cell Biol. *11*, 1010–1016.

Zillner, K., Filarsky, M., Rachow, K., Weinberger, M., Längst, G., and Németh, A. (2013). Large-scale organization of ribosomal DNA chromatin is regulated by Tip5. Nucleic Acids Res. *41*, 5251–5262.

 ©2015 The Authors

Structure, Volume *23*

Supplemental Information

Molecular Basis of Histone Tail Recognition by Human TIP5 PHD Finger and Bromodomain of the Chromatin Remodeling Complex NoRC

Cynthia Tallant, Erica Valentini, Oleg Fedorov, Lois Overvoorde, Fleur M. Ferguson, Panagis Filippakopoulos, Dmitri I. Svergun, Stefan Knapp, and Alessio Ciulli

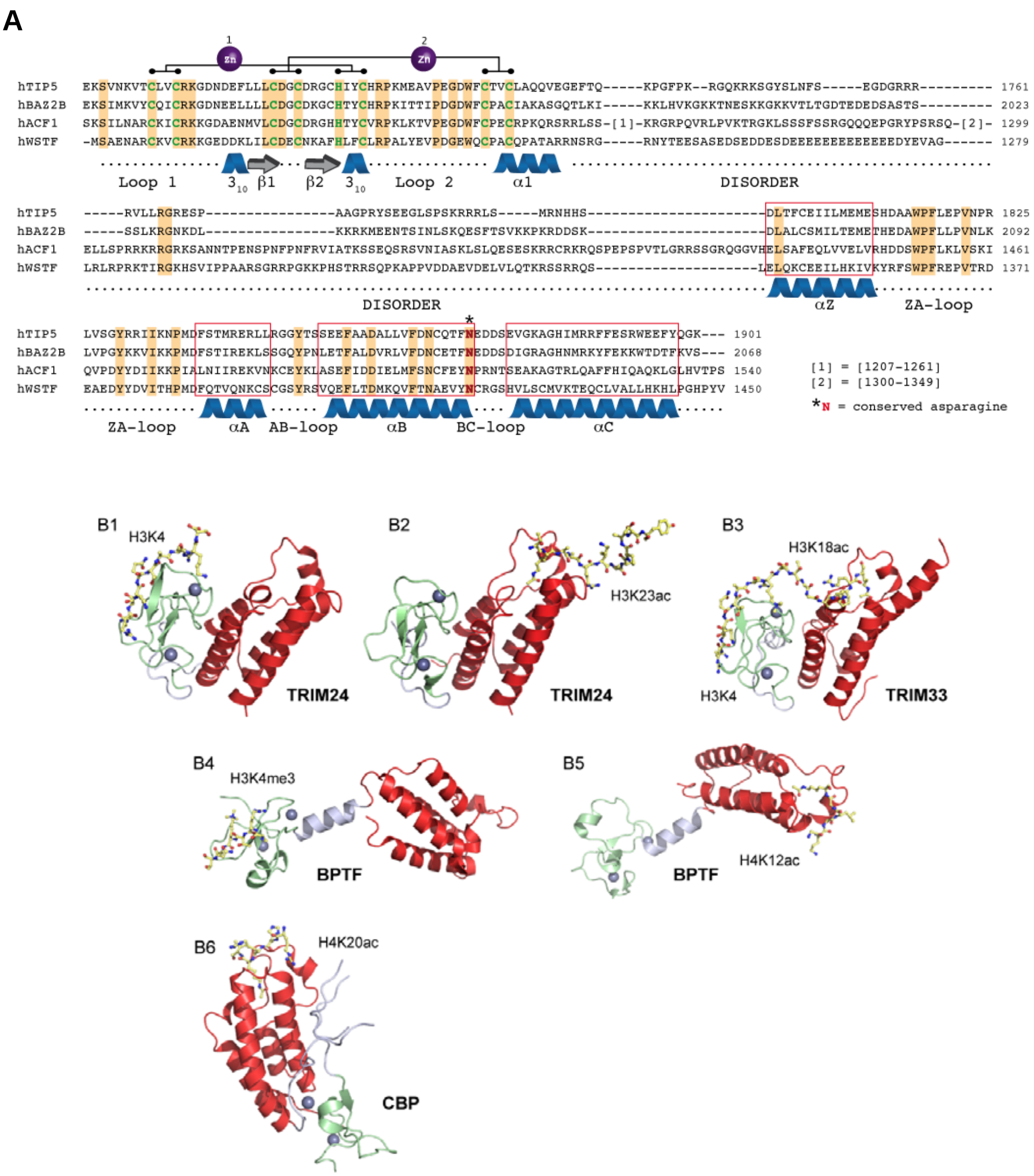

Figure S1 (related to Figure 1). A. Structure-based sequence alignment of the PHD zinc finger and bromodomain region of human chromatin remodelled proteins: hTIP5 (Q9UIF9_BAZ2A), hBAZ2B (Q9UIF8_BAZ2B), hACF1 (Q9NRL2_BAZ1A) and hWSTF (Q9UIG0_BAZ1B). **B.** Histone bound crystal structures of related proteins with PHD zinc fingers adjacent to bromodomains. **B1**. TRIM24 PHD-Bromo in complex with H3(1-10)K4 histone (3O37.pdb). **B2**. TRIM24 PHD-Bromo in complex with H3(13-32)K23ac histone (3O34.pdb). **B3**. TRIM33 PHD-Bromo in complex of single H3(1-28) K9me3K14acK18acK23ac histone (3U5P.pdb). **B4**. BPTF PHD-linker-bromodomain in complex with H3(1-15)K4me3 histone (2F6J.pdb). **B5**. BPTF PHD-linker-bromodomain in complex with H4(7-12)K12ac histone (3QZV.pdb). **B6**. CBP Bromodomain-PHD in complex with H4(13-26)K20ac histone (4N3W.pdb).

A

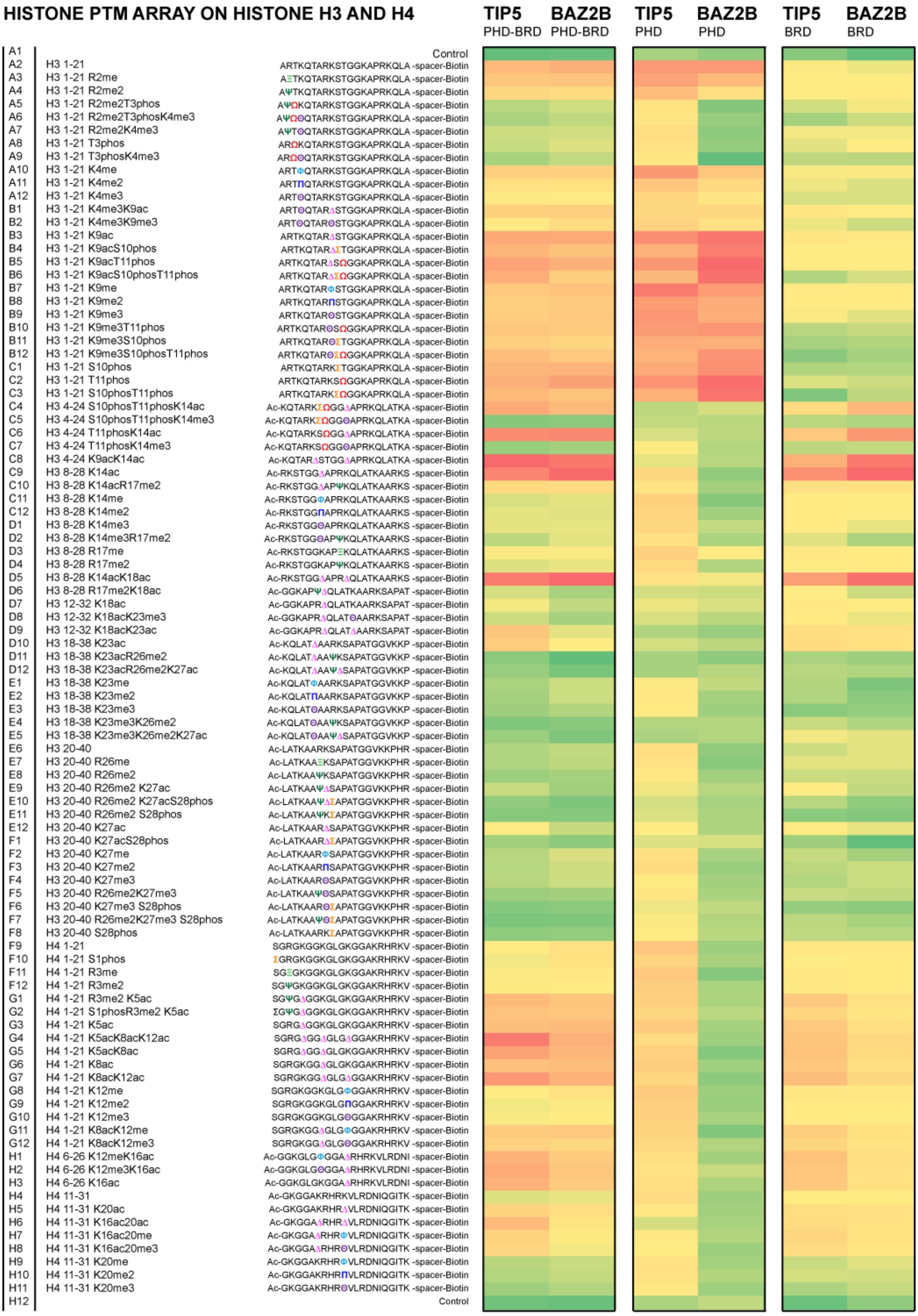
HISTONE PTM ARRAY ON HISTONE H3 AND H4
TIP5 PHD-BRD
BAZ2B PHD-BRD
TIP5 PHD
BAZ2B PHD
TIP5 BRD
BAZ2B BRD
A1 Control
A2 H3 1-21 ARTKQTARKSTGGKAPRKQLA -spacer-Biotin
A3 H3 1-21 R2me AΞTKQTARKSTGGKAPRKQLA -spacer-Biotin
A4 H3 1-21 R2me2 AΨTKQTARKSTGGKAPRKQLA -spacer-Biotin
A5 H3 1-21 R2me2T3phos AΨΩKQTARKSTGGKAPRKQLA -spacer-Biotin
A6 H3 1-21 R2me2T3phosK4me3 AΨΩΘQTARKSTGGKAPRKQLA -spacer-Biotin
A7 H3 1-21 R2me2K4me3 AΨTΘQTARKSTGGKAPRKQLA -spacer-Biotin
A8 H3 1-21 T3phos ARΩKQTARKSTGGKAPRKQLA -spacer-Biotin
A9 H3 1-21 T3phosK4me3 ARΩΘQTARKSTGGKAPRKQLA -spacer-Biotin
A10 H3 1-21 K4me ARTΦQTARKSTGGKAPRKQLA -spacer-Biotin
A11 H3 1-21 K4me2 ARTΠQTARKSTGGKAPRKQLA -spacer-Biotin
A12 H3 1-21 K4me3 ARTΘQTARKSTGGKAPRKQLA -spacer-Biotin
B1 H3 1-21 K4me3K9ac ARTΘQTARΔSTGGKAPRKQLA -spacer-Biotin
B2 H3 1-21 K4me3K9me3 ARTΘQTARΘSTGGKAPRKQLA -spacer-Biotin
B3 H3 1-21 K9ac ARTKQTARΔSTGGKAPRKQLA -spacer-Biotin
B4 H3 1-21 K9acS10phos ARTKQTARΔΣTGGKAPRKQLA -spacer-Biotin
B5 H3 1-21 K9acT11phos ARTKQTARΔSΩGGKAPRKQLA -spacer-Biotin
B6 H3 1-21 K9acS10phosT11phos ARTKQTARΔΣΩGGKAPRKQLA -spacer-Biotin
B7 H3 1-21 K9me ARTKQTARΦSTGGKAPRKQLA -spacer-Biotin
B8 H3 1-21 K9me2 ARTKQTARΠSTGGKAPRKQLA -spacer-Biotin
B9 H3 1-21 K9me3 ARTKQTARΘSTGGKAPRKQLA -spacer-Biotin
B10 H3 1-21 K9me3T11phos ARTKQTARΘSΩGGKAPRKQLA -spacer-Biotin
B11 H3 1-21 K9me3S10phos ARTKQTARΘΣTGGKAPRKQLA -spacer-Biotin
B12 H3 1-21 K9me3S10phosT11phos ARTKQTARΘΣΩGGKAPRKQLA -spacer-Biotin
C1 H3 1-21 S10phos ARTKQTARKΣTGGKAPRKQLA -spacer-Biotin
C2 H3 1-21 T11phos ARTKQTARKSΩGGKAPRKQLA -spacer-Biotin
C3 H3 1-21 S10phosT11phos ARTKQTARKΣΩGGKAPRKQLA -spacer-Biotin
C4 H3 4-24 S10phosT11phosK14ac Ac-KQTARKΣΩGGΔAPRKQLATKA -spacer-Biotin
C5 H3 4-24 S10phosT11phosK14me3 Ac-KQTARKΣΩGGΘAPRKQLATKA -spacer-Biotin
C6 H3 4-24 T11phosK14ac Ac-KQTARKSΩGGΔAPRKQLATKA -spacer-Biotin
C7 H3 4-24 T11phosK14me3 Ac-KQTARKSΩGGΘAPRKQLATKA -spacer-Biotin
C8 H3 4-24 K9acK14ac Ac-KQTARΔSTGGΔAPRKQLATKA -spacer-Biotin
C9 H3 8-28 K14ac Ac-RKSTGGΔAPRKQLATKAARKS -spacer-Biotin
C10 H3 8-28 K14acR17me2 Ac-RKSTGGΔAPΨKQLATKAARKS -spacer-Biotin
C11 H3 8-28 K14me Ac-RKSTGGΦAPRKQLATKAARKS -spacer-Biotin
C12 H3 8-28 K14me2 Ac-RKSTGGΠAPRKQLATKAARKS -spacer-Biotin
D1 H3 8-28 K14me3 Ac-RKSTGGΘAPRKQLATKAARKS -spacer-Biotin
D2 H3 8-28 K14me3R17me2 Ac-RKSTGGΘAPΨKQLATKAARKS -spacer-Biotin
D3 H3 8-28 R17me Ac-RKSTGGKAPΞKQLATKAARKS -spacer-Biotin
D4 H3 8-28 R17me2 Ac-RKSTGGKAPΨKQLATKAARKS -spacer-Biotin
D5 H3 8-28 K14acK18ac Ac-RKSTGGΔAPRΔQLATKAARKS -spacer-Biotin
D6 H3 8-28 R17me2K18ac Ac-GGKAPΨΔQLATKAARKSAPAT -spacer-Biotin
D7 H3 12-32 K18ac Ac-GGKAPRΔQLATKAARKSAPAT -spacer-Biotin
D8 H3 12-32 K18acK23me3 Ac-GGKAPRΔQLATΘAARKSAPAT -spacer-Biotin
D9 H3 12-32 K18acK23ac Ac-GGKAPRΔQLATΔAARKSAPAT -spacer-Biotin
D10 H3 18-38 K23ac Ac-KQLATΔAARKSAPATGGVKKP -spacer-Biotin
D11 H3 18-38 K23acR26me2 Ac-KQLATΔAAΨKSAPATGGVKKP -spacer-Biotin
D12 H3 18-38 K23acR26me2K27ac Ac-KQLATΔAAΨΔSAPATGGVKKP -spacer-Biotin
E1 H3 18-38 K23me Ac-KQLATΦAARKSAPATGGVKKP -spacer-Biotin
E2 H3 18-38 K23me2 Ac-KQLATΠAARKSAPATGGVKKP -spacer-Biotin
E3 H3 18-38 K23me3 Ac-KQLATΘAARKSAPATGGVKKP -spacer-Biotin
E4 H3 18-38 K23me3K26me2 Ac-KQLATΘAAΨKSAPATGGVKKP -spacer-Biotin
E5 H3 18-38 K23me3K26me2K27ac Ac-KQLATΘAAΨΔSAPATGGVKKP -spacer-Biotin
E6 H3 20-40 Ac-LATKAARKSAPATGGVKKPHR -spacer-Biotin
E7 H3 20-40 R26me Ac-LATKAAΞKSAPATGGVKKPHR -spacer-Biotin
E8 H3 20-40 R26me2 Ac-LATKAAΨKSAPATGGVKKPHR -spacer-Biotin
E9 H3 20-40 R26me2 K27ac Ac-LATKAAΨΔSAPATGGVKKPHR -spacer-Biotin
E10 H3 20-40 R26me2 K27acS28phos Ac-LATKAAΨΔΣAPATGGVKKPHR -spacer-Biotin
E11 H3 20-40 R26me2 S28phos Ac-LATKAAΨKΣAPATGGVKKPHR -spacer-Biotin
E12 H3 20-40 K27ac Ac-LATKAARΔSAPATGGVKKPHR -spacer-Biotin
F1 H3 20-40 K27acS28phos Ac-LATKAARΔΣAPATGGVKKPHR -spacer-Biotin
F2 H3 20-40 K27me Ac-LATKAARΦSAPATGGVKKPHR -spacer-Biotin
F3 H3 20-40 K27me2 Ac-LATKAARΠSAPATGGVKKPHR -spacer-Biotin
F4 H3 20-40 K27me3 Ac-LATKAARΘSAPATGGVKKPHR -spacer-Biotin
F5 H3 20-40 R26me2K27me3 Ac-LATKAAΨΘSAPATGGVKKPHR -spacer-Biotin
F6 H3 20-40 K27me3 S28phos Ac-LATKAARΘΣAPATGGVKKPHR -spacer-Biotin
F7 H3 20-40 R26me2K27me3 S28phos Ac-LATKAAΨΘΣAPATGGVKKPHR -spacer-Biotin
F8 H3 20-40 S28phos Ac-LATKAARKΣAPATGGVKKPHR -spacer-Biotin
F9 H4 1-21 SGRGKGGKGLGKGGAKRHRKV -spacer-Biotin
F10 H4 1-21 S1phos ΣGRGKGGKGLGKGGAKRHRKV -spacer-Biotin
F11 H4 1-21 R3me SGΞGKGGKGLGKGGAKRHRKV -spacer-Biotin
F12 H4 1-21 R3me2 SGΨGKGGKGLGKGGAKRHRKV -spacer-Biotin
G1 H4 1-21 R3me2 K5ac SGΨGΔGGKGLGKGGAKRHRKV -spacer-Biotin
G2 H4 1-21 S1phosR3me2 K5ac ΣGΨGΔGGKGLGKGGAKRHRKV -spacer-Biotin
G3 H4 1-21 K5ac SGRGΔGGKGLGKGGAKRHRKV -spacer-Biotin
G4 H4 1-21 K5acK8acK12ac SGRGΔGGΔGLGΔGGAKRHRKV -spacer-Biotin
G5 H4 1-21 K5acK8ac SGRGΔGGΔGLGKGGAKRHRKV -spacer-Biotin
G6 H4 1-21 K8ac SGRGKGGΔGLGKGGAKRHRKV -spacer-Biotin
G7 H4 1-21 K8acK12ac SGRGKGGΔGLGΔGGAKRHRKV -spacer-Biotin
G8 H4 1-21 K12me SGRGKGGKGLGΦGGAKRHRKV -spacer-Biotin
G9 H4 1-21 K12me2 SGRGKGGKGLGΠGGAKRHRKV -spacer-Biotin
G10 H4 1-21 K12me3 SGRGKGGKGLGΘGGAKRHRKV -spacer-Biotin
G11 H4 1-21 K8acK12me SGRGKGGΔGLGΦGGAKRHRKV -spacer-Biotin
G12 H4 1-21 K8acK12me3 SGRGKGGΔGLGΘGGAKRHRKV -spacer-Biotin
H1 H4 6-26 K12meK16ac Ac-GGKGLGΦGGAΔRHRKVLRDNI -spacer-Biotin
H2 H4 6-26 K12me3K16ac Ac-GGKGLGΘGGAΔRHRKVLRDNI -spacer-Biotin
H3 H4 6-26 K16ac Ac-GGKGLGKGGAΔRHRKVLRDNI -spacer-Biotin
H4 H4 11-31 Ac-GKGGAKRHRKVLRDNIQGITK -spacer-Biotin
H5 H4 11-31 K20ac Ac-GKGGAKRHRΔVLRDNIQGITK -spacer-Biotin
H6 H4 11-31 K16ac20ac Ac-GKGGAΔRHRΔVLRDNIQGITK -spacer-Biotin
H7 H4 11-31 K16ac20me Ac-GKGGAΔRHRΦVLRDNIQGITK -spacer-Biotin
H8 H4 11-31 K16ac20me3 Ac-GKGGAΔRHRΘVLRDNIQGITK -spacer-Biotin
H9 H4 11-31 K20me Ac-GKGGAKRHRΦVLRDNIQGITK -spacer-Biotin
H10 H4 11-31 K20me2 Ac-GKGGAKRHRΠVLRDNIQGITK -spacer-Biotin
H11 H4 11-31 K20me3 Ac-GKGGAKRHRΘVLRDNIQGITK -spacer-Biotin
H12 Control

B

HISTONE ACETYL LYSINE ARRAY ON H2A, H2B, H3, H4

Spot	Peptide	Sequence
A1		Control
A2	H4 1-21	SGRGKGGKGLGKGGAKRHRKV-spacer-Biotin
A3	H4 1-21 K5ac	SGRG△GGKGLGKGGAKRHRKV-spacer-Biotin
A4	H4 1-21 K8ac	SGRGKGG△GLGKGGAKRHRKV-spacer-Biotin
A5	H4 1-21 K12ac	SGRGKGGKGLG△GGAKRHRKV-spacer-Biotin
A6	H4 1-21 K16ac	SGRGKGGKGLGKGGA△RHRKV-spacer-Biotin
A7	H4 1-21 K5acK8ac	SGRG△GG△GLGKGGAKRHRKV-spacer-Biotin
A8	H4 1-21 K5acK12ac	SGRG△GGKGLG△GGAKRHRKV-spacer-Biotin
A9	H4 1-21 K5acK16ac	SGRG△GGKGLGKGGA△RHRKV-spacer-Biotin
A10	H4 1-21 K8acK12ac	SGRGKGG△GLG△GGAKRHRKV-spacer-Biotin
A11	H4 1-21 K8acK16ac	SGRGKGG△GLGKGGA△RHRKV-spacer-Biotin
A12	H4 1-21 K12acK16ac	SGRGKGGKGLG△GGA△RHRKV-spacer-Biotin
B1	H4 1-21 K5acK8acK12ac	SGRG△GG△GLG△GGAKRHRKV-spacer-Biotin
B2	H4 1-21 K8acK12acK16ac	SGRGKGG△GLG△GGA△RHRKV-spacer-Biotin
B3	H4 1-21 K5acK12acK16ac	SGRG△GGKGLG△GGA△RHRKV-spacer-Biotin
B4	H4 1-21 K5acK8acK16ac	SGRG△GG△GLGKGGA△RHRKV-spacer-Biotin
B5	H4 1-21 K5acK8acK12acK16ac	SGRG△GG△GLG△GGA△RHRKV-spacer-Biotin
B6	H4 9-29	Ac-GLGKGGAKRHRKVLRDNIQGI-spacer-Biotin
B7	H4 9-29 K20ac	Ac-GLGKGGAKRHR△VLRDNIQGI-spacer-Biotin
B8	H4 9-29 K16ac	Ac-GLGKGGA△RHRKVLRDNIQGI-spacer-Biotin
B9	H4 9-29 K12acK16ac	Ac-GLG△GGA△RHRKVLRDNIQGI-spacer-Biotin
B10	H4 9-29 K16acK20ac	Ac-GLGKGGA△RHR△VLRDNIQGI-spacer-Biotin
B11	H4 9-29 K12acK16acK20ac	Ac-GLG△GGA△RHR△VLRDNIQGI-spacer-Biotin
B12	H2a 1-21	SGRGKQGGKARAKAKTRSSRA-spacer-Biotin
C1	H2a 1-21 K5ac	SGRG△QGGKARAKAKTRSSRA-spacer-Biotin
C2	H2a 1-21 K9ac	SGRGKQGG△ARAKAKTRSSRA-spacer-Biotin
C3	H2a 1-21 K13ac	SGRGKQGGKARA△AKTRSSRA-spacer-Biotin
C4	H2a 1-21 K15ac	SGRGKQGGKARAKA△TRSSRA-spacer-Biotin
C5	H2a 1-21 K5acK9ac	SGRG△QGG△ARAKAKTRSSRA-spacer-Biotin
C6	H2a 1-21 K5acK13ac	SGRG△QGGKARA△AKTRSSRA-spacer-Biotin
C7	H2a 1-21 K5acK15ac	SGRG△QGGKARAKA△TRSSRA-spacer-Biotin
C8	H2a 1-21 K9acK13ac	SGRGKQGG△ARA△AKTRSSRA-spacer-Biotin
C9	H2a 1-21 K9acK15ac	SGRGKQGG△ARAKA△TRSSRA-spacer-Biotin
C10	H2a 1-21 K13acK15ac	SGRGKQGGKARA△A△TRSSRA-spacer-Biotin
C11	H2a 1-21 K5acK9acK13ac	SGRG△QGG△ARA△AKTRSSRA-spacer-Biotin
C12	H2a 1-21 K5acK13acK15ac	SGRG△QGGKARA△A△TRSSRA-spacer-Biotin
D1	H2a 1-21 K9acK13acK15ac	SGRGKQGG△ARA△A△TRSSRA-spacer-Biotin
D2	H2a 1-21 K5acK9acK15ac	SGRG△QGG△ARAKA△TRSSRA-spacer-Biotin
D3	H2a 1-21 K5acK9acK13acK15ac	SGRG△QGG△ARA△A△TRSSRA-spacer-Biotin
D4	H2b 1-21	PEPAKSAPAPKKGSKKAVTKA-spacer-Biotin
D5	H2b 1-21 K5ac	PEPA△SAPAPKKGSKKAVTKA-spacer-Biotin
D6	H2b 1-21 K11ac	PEPAKSAPAP△KGSKKAVTKA-spacer-Biotin
D7	H2b 1-21 K12ac	PEPAKSAPAPK△GSKKAVTKA-spacer-Biotin
D8	H2b 1-21 K15ac	PEPAKSAPAPKKGS△KAVTKA-spacer-Biotin
D9	H2b 1-21 K5acK11acK16ac	PEPA△SAPAP△KGSK△AVTKA-spacer-Biotin
D10	H2b 1-21 K5acK12ac	PEPA△SAPAPK△GSKKAVTKA-spacer-Biotin
D11	H2b 1-21 K5acK15ac	PEPA△SAPAPKKGS△KAVTKA-spacer-Biotin
D12	H2b 1-21 K5acK16ac	PEPA△SAPAPKKGSK△AVTKA-spacer-Biotin
E1	H2b 1-21 K11acK12ac	PEPAKSAPAP△△GSKKAVTKA-spacer-Biotin
E2	H2b 1-21 K11acK15ac	PEPAKSAPAP△KGS△KAVTKA-spacer-Biotin
E3	H2b 1-21 K11acK16ac	PEPAKSAPAP△KGSK△AVTKA-spacer-Biotin
E4	H2b 1-21 K5acK11acK12ac	PEPA△SAPAP△△GSKKAVTKA-spacer-Biotin
E5	H2b 1-21 K5acK15acK16ac	PEPA△SAPAPKKGS△△AVTKA-spacer-Biotin
E6	H2b 1-21 K11acK12acK15acK16ac	PEPAKSAPAP△△GS△△AVTKA-spacer-Biotin
E7	H2b 1-21 K11acK15acK16ac	PEPAKSAPAP△KGS△△AVTKA-spacer-Biotin
E8	H2b 1-21 K12acK15acK16ac	PEPAKSAPAPK△GS△△AVTKA-spacer-Biotin
E9	H2b 1-21 K11acK12acK15ac	PEPAKSAPAP△△GS△KAVTKA-spacer-Biotin
E10	H2b 1-21 K11acK12acK16ac	PEPAKSAPAP△△GSK△AVTKA-spacer-Biotin
E11	H2b 1-21 K5acK11acK12acK15acK16ac	PEPA△SAPAP△△GS△△AVTKA-spacer-Biotin
E12	H2b 13-33	Ac-GSKKAVTKAQKKDGKKRKRSR-spacer-Biotin
F1	H2b 13-33 K20ac	Ac-GSKKAVT△AQKKDGKKRKRSR-spacer-Biotin
F2	H2b 13-33 K23ac	Ac-GSKKAVTKAQ△KDGKKRKRSR-spacer-Biotin
F3	H2b 13-33 K24ac	Ac-GSKKAVTKAQK△DGKKRKRSR-spacer-Biotin
F4	H2b 13-33 K20acK23acK24ac	Ac-GSKKAVT△AQ△△DGKKRKRSR-spacer-Biotin
F5	H3 1-21	ARTKQTARKSTGGKAPRKQLA-spacer-Biotin
F6	H3 1-21 K4ac	ART△QTARKSTGGKAPRKQLA-spacer-Biotin
F7	H3 1-21 K9ac	ARTKQTAR△STGGKAPRKQLA-spacer-Biotin
F8	H3 1-21 K14ac	ARTKQTARKSTGG△APRKQLA-spacer-Biotin
F9	H3 1-21 K4acK9ac	ART△QTAR△STGGKAPRKQLA-spacer-Biotin
F10	H3 1-21 K4acK14ac	ART△QTARKSTGG△APRKQLA-spacer-Biotin
F11	H3 1-21 K9acK14ac	ARTKQTAR△STGG△APRKQLA-spacer-Biotin
F12	H3 1-21 K4acK9acK14ac	ART△QTAR△STGG△APRKQLA-spacer-Biotin
G1	H3 11-31	Ac-TGGKAPRKQLATKAARKSAPA-spacer-Biotin
G2	H3 11-31 K14ac	Ac-TGG△APRKQLATKAARKSAPA-spacer-Biotin
G3	H3 11-31 K18ac	Ac-TGGKAPR△QLATKAARKSAPA-spacer-Biotin
G4	H3 11-31 K23ac	Ac-TGGKAPRKQLAT△AARKSAPA-spacer-Biotin
G5	H3 11-31 K27ac	Ac-TGGKAPRKQLATKAAR△SAPA-spacer-Biotin
G6	H3 11-31 K14acK18ac	Ac-TGG△APR△QLATKAARKSAPA-spacer-Biotin
G7	H3 11-31 K14acK23ac	Ac-TGG△APRKQLAT△AARKSAPA-spacer-Biotin
G8	H3 11-31 K14acK27ac	Ac-TGG△APRKQLATKAAR△SAPA-spacer-Biotin
G9	H3 11-31 K18acK23ac	Ac-TGGKAPR△QLAT△AARKSAPA-spacer-Biotin
G10	H3 11-31 K18acK27ac	Ac-TGGKAPR△QLATKAAR△SAPA-spacer-Biotin
G11	H3 11-31 K23acK27ac	Ac-TGGKAPRKQLAT△AAR△SAPA-spacer-Biotin
G12	H3 11-31 K14acK23acK27ac	Ac-TGG△APRKQLAT△AAR△SAPA-spacer-Biotin
H1	H3 11-31 K18acK23acK27ac	Ac-TGGKAPR△QLAT△AAR△SAPA-spacer-Biotin
H2	H3 11-31 K14acK18acK23ac	Ac-TGG△APR△QLATKAAR△SAPA-spacer-Biotin
H3	H3 11-31 K14acK18acK23acK27ac	Ac-TGG△APR△QLAT△AAR△SAPA-spacer-Biotin
H4	H3 23-43	Ac-KAARKSAPATGGVKKPHRYRP-spacer-Biotin
H5	H3 23-43 K36ac	Ac-KAARKSAPATGGV△KPHRYRP-spacer-Biotin
H6	H3 23-43 K37ac	Ac-KAARKSAPATGGVK△PHRYRP-spacer-Biotin
H7	H3 23-43 K36acK37ac	Ac-KAARKSAPATGGV△△PHRYRP-spacer-Biotin
H8	H3 23-43 K27ac	Ac-KAAR△SAPATGGVKKPHRYRP-spacer-Biotin
H9	H3 23-43 K27acK36ac	Ac-KAAR△SAPATGGV△KPHRYRP-spacer-Biotin
H10	H3 23-43 K27acK37ac	Ac-KAAR△SAPATGGVK△PHRYRP-spacer-Biotin
H11	H3 23-43 K27acK36acK37ac	Ac-KAAR△SAPATGGV△△PHRYRP-spacer-Biotin
H12		Control

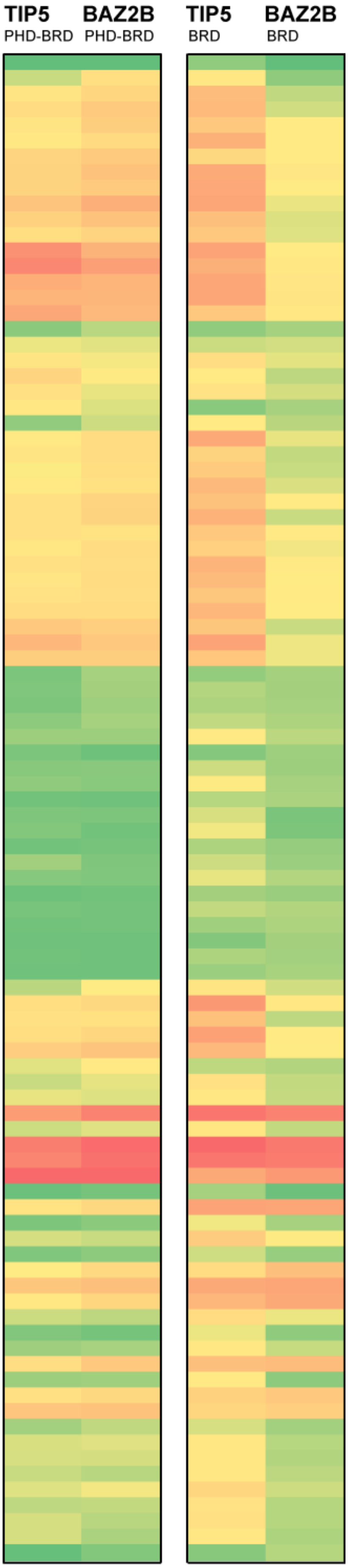

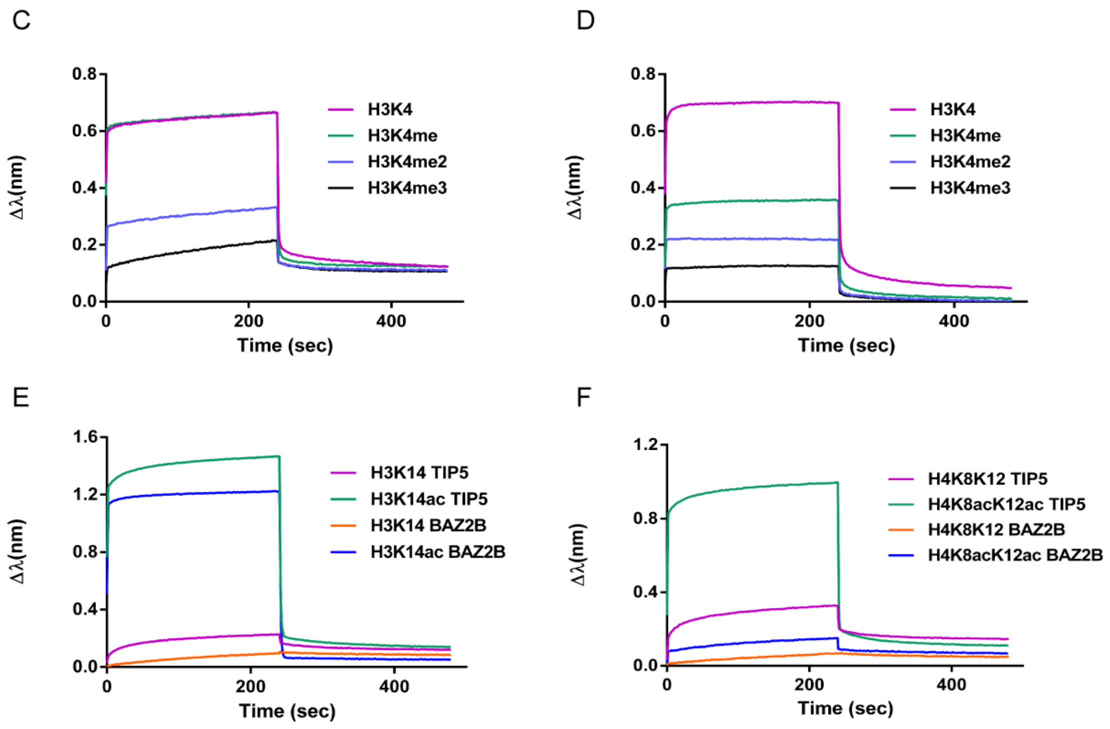

Figure S2 (related to Table 1). A. Heat map obtained from biolayer interferometry measurements against histone peptide arrays. Peptide symbol key: Δ = acetyl-Lys, φ = monomethyl-Lys, Π = dimethyl-Lys, Θ = trimethyl-Lys, Σ = phopho-Ser, Ω = phosphor-Thr, Ξ = monomethy-Arg, Ψ = asymdimethyl-Arg. Post-translational modification distributed to H3 and H4 histone array. **B**. Specific histone array for H2A, H2B, H3 and H4 acetylation marks. **C.** Association and dissociation curves of BLI measurements of TIP5 PHD finger binding towards unmodified/mono/di/trimethylation to H3K4. **D** BAZ2B PHD finger binding towards unmodified/mono/di/trimethylation to H3K4. **E** H3K14ac recognition for TIP5 and BAZ2B bromodomains. **F** Comparison of unmodified/acetylated of H4K8K12 for TIP5 and BAZ2B bromodomains.

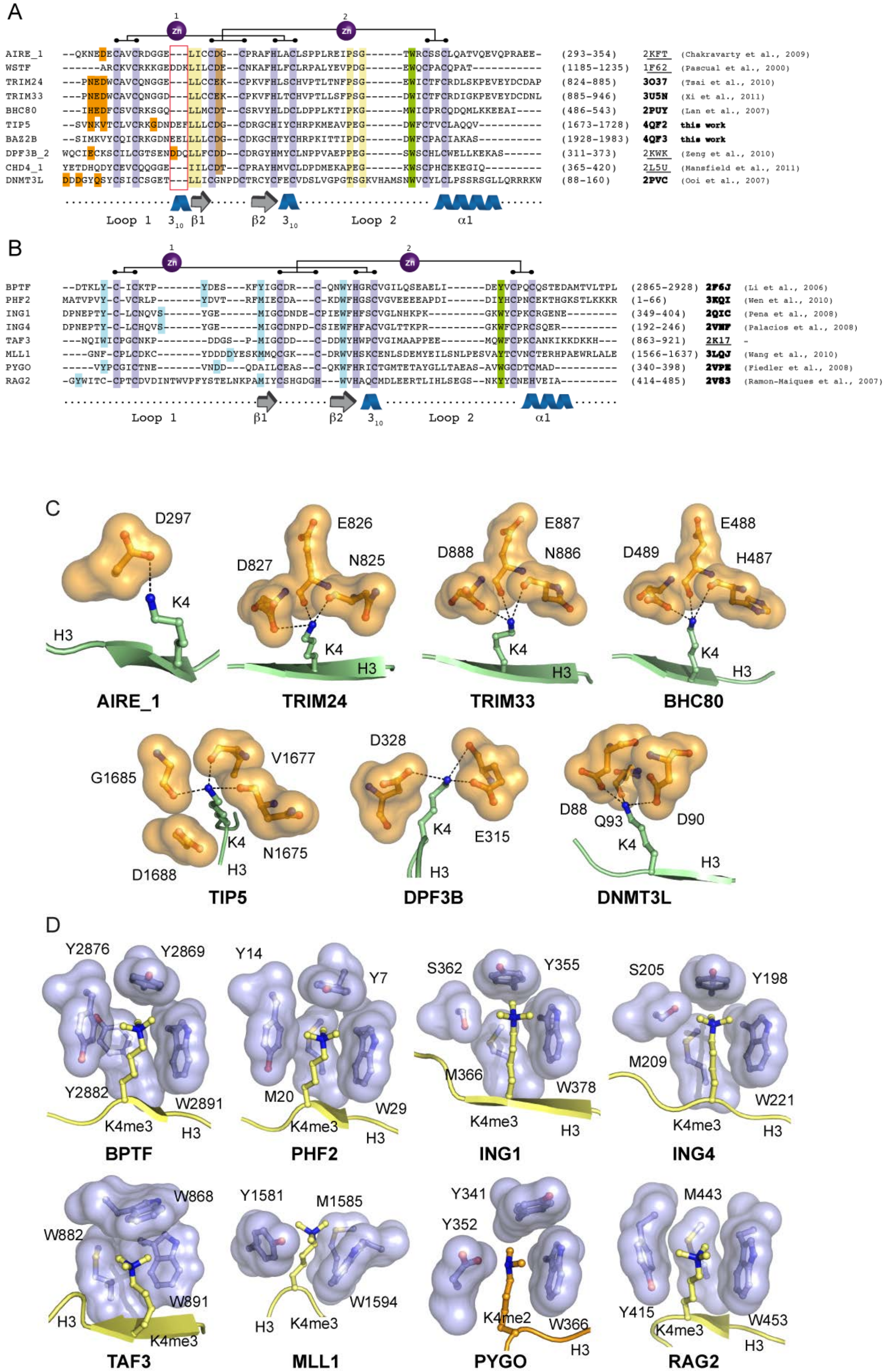
A
AIRE_1
WSTF
TRIM24
TRIM33
BHC80
TIP5
BAZ2B
DPF3B_2
CHD4_1
DNMT3L
(293-354) 2KFT (Chakravarty et al., 2009)
(1185-1235) 1F62 (Pascual et al., 2000)
(824-885) 3O37 (Tsai et al., 2010)
(885-946) 3U5N (Xi et al., 2011)
(486-543) 2PUY (Lan et al., 2007)
(1673-1728) 4QF2 this work
(1928-1983) 4QF3 this work
(311-373) 2KWK (Zeng et al., 2010)
(365-420) 2L5U (Mansfield et al., 2011)
(88-160) 2PVC (Ooi et al., 2007)
Loop 1
β1
β2
Loop 2
α1
B
BPTF
PHF2
ING1
ING4
TAF3
MLL1
PYGO
RAG2
(2865-2928) 2F6J (Li et al., 2006)
(1-66) 3KQI (Wen et al., 2010)
(349-404) 2QIC (Pena et al., 2008)
(192-246) 2VNF (Palacios et al., 2008)
(863-921) 2K17 -
(1566-1637) 3LQJ (Wang et al., 2010)
(340-398) 2VPE (Fiedler et al., 2008)
(414-485) 2V83 (Ramon-Maiques et al., 2007)
Loop 1
β1
β2
Loop 2
α1
C
D297
K4
H3
AIRE_1
E826
D827
N825
K4
H3
TRIM24
E887
D888
N886
K4
H3
TRIM33
E488
D489
H487
K4
H3
BHC80
G1685
V1677
N1675
K4
H3
D1688
TIP5
D328
K4
E315
H3
DPF3B
D88
Q93
D90
K4
H3
DNMT3L
D
Y2876
Y2869
Y2882
W2891
K4me3
H3
BPTF
Y14
Y7
M20
W29
K4me3
H3
PHF2
S362
Y355
M366
W378
K4me3
H3
ING1
S205
Y198
M209
W221
K4me3
H3
ING4
W868
W882
W891
H3
K4me3
TAF3
Y1581
M1585
W1594
H3
K4me3
MLL1
Y341
Y352
K4me2
W366
H3
PYGO
M443
Y415
K4me3
W453
H3
RAG2

Figure S3 (related to Figure 2). A, B Structure-based sequence alignment of histone recognition PHD zinc fingers. The domain length is indicated in brackets. Their related Protein Data Bank accession codes are also shown underlined and in bold for NMR and X-ray structures, respectively. Together, PHD fingers share secondary structure elements and have similar protein folding but they exhibit high sequence variability. The two zinc atoms are shown in purple spheres and are linked to their corresponding pairs of zinc-coordinating residues (light pink), which are highly conserved to all PHD fingers. A tryptophan or tyrosine (green) located at the end of Loop 2 is also a bulky hydrophobic conserved position pointing to the core of the PHD finger to maintain its integrity. Most of the H3K4 recognition residues are found in loop 1. **A**. Unmethylated histone recognition. The sequences are (with GenBank accession numbers): AIRE_1 (O43918 AIRE_HUMAN); WSTF (Q9UIG0 BAZ1B_HUMAN); TRIM24 (O15164 TIF1α_HUMAN); TRIM33 (Q9UPN9 TIF1γ_HUMAN); BHC80 (Q96BD5 PF21A_HUMAN); TIP5 (Q9UIF9 BAZ2A_HUMAN); BAZ2B (Q9UIF8 BAZ2B_HUMAN); DPF3B_2 (Q92784 DPF3_HUMAN); CHD4_1 (Q14839 CHD4_HUMAN); DNMT3L (Q9UJW3 DNM3L_HUMAN). The residues involved in the K4 recognition are highlighted in orange. **B**. Methylated histone recognition. Sequence alignment of methylated histone recognition PHD fingers. It includes: BPTF (Q12830 BPTF_HUMAN); PHF2 (**O75151** PHF2_HUMAN); ING1 (Q9UK53 ING1_HUMAN); ING4 (Q9UNL4 ING4_HUMAN); TAF3 (Q5VWG9 (TAF3_HUMAN); MLL1 (Q03164 KMT2A_HUMAN); PYGO (Q9Y3Y4 PYGO1_HUMAN); RAG2 (P55895 RAG2_HUMAN). The residues involved in the K4me2/me3 recognition are highlighted in light blue. A highly conserved tryptophan at the β2 strand is essential for the aromatic cage formation. **C**. Close-up view of histone recognition sites of the subclass of H3K4me0-specific PHD fingers. In most cases they possess a cluster of acidic residues and carbonyl groups from amide bonds forming hydrogen bonds and salt bridges with the side chain of unmodified K4. Methylation states of K4 decrease or supress these interactions. **D**. Close-up view of histone recognition sites of the subclass of H3K4me2/3-specific PHD fingers. These pockets form aromatic cages with different combinations of two to four aromatic residues. A tryptophan is conserved in all of them (see sequence alignment above). The residue arrangement favours the formation of cation-π, hydrophobic and van der Waals contacts with the di/trimethylammonium moiety of K4. The coordinates for all structures are listed in A, B.

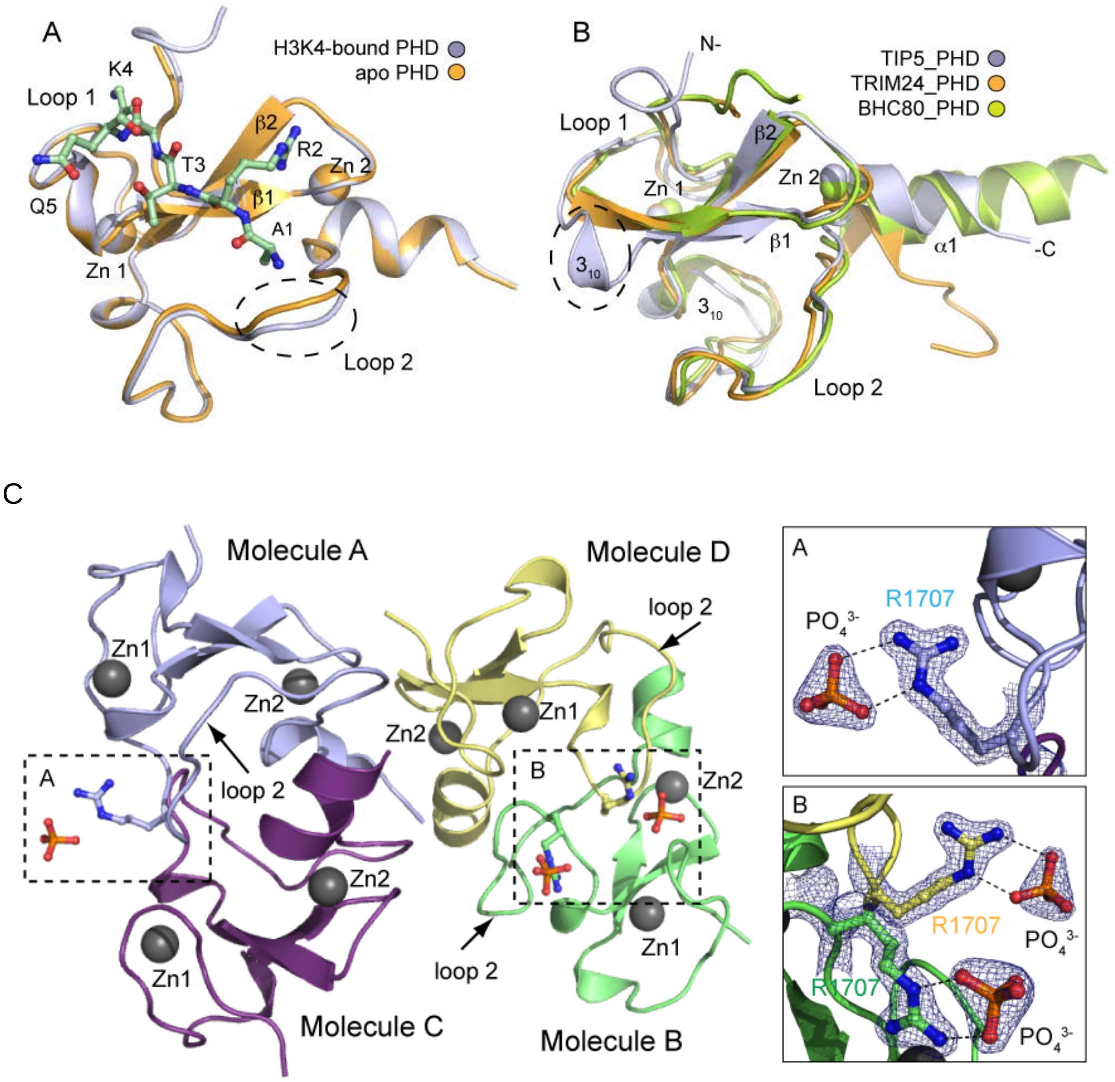

Figure S4 (related to Figure 2 and Table 2). A. TIP5 PHD zinc finger Cα superposition of free state and in complex with H3K4. **B**. Superposition of TIP5, TRIM24, BHC80 in complex structures (the H3K4 peptide has been omitted for the figure to highlight the difference in the surface-exposed cavity). **C.** The asymmetric unit of TIP5 PHD zinc finger in the free state. The four molecules that constitute the asymmetric unit are shown in different colours and displayed in cartoon representation. The exposed side chain of R1707 located at the beginning of loop 2 shows promiscuous affinity towards phosphate molecules from the crystallization buffer.

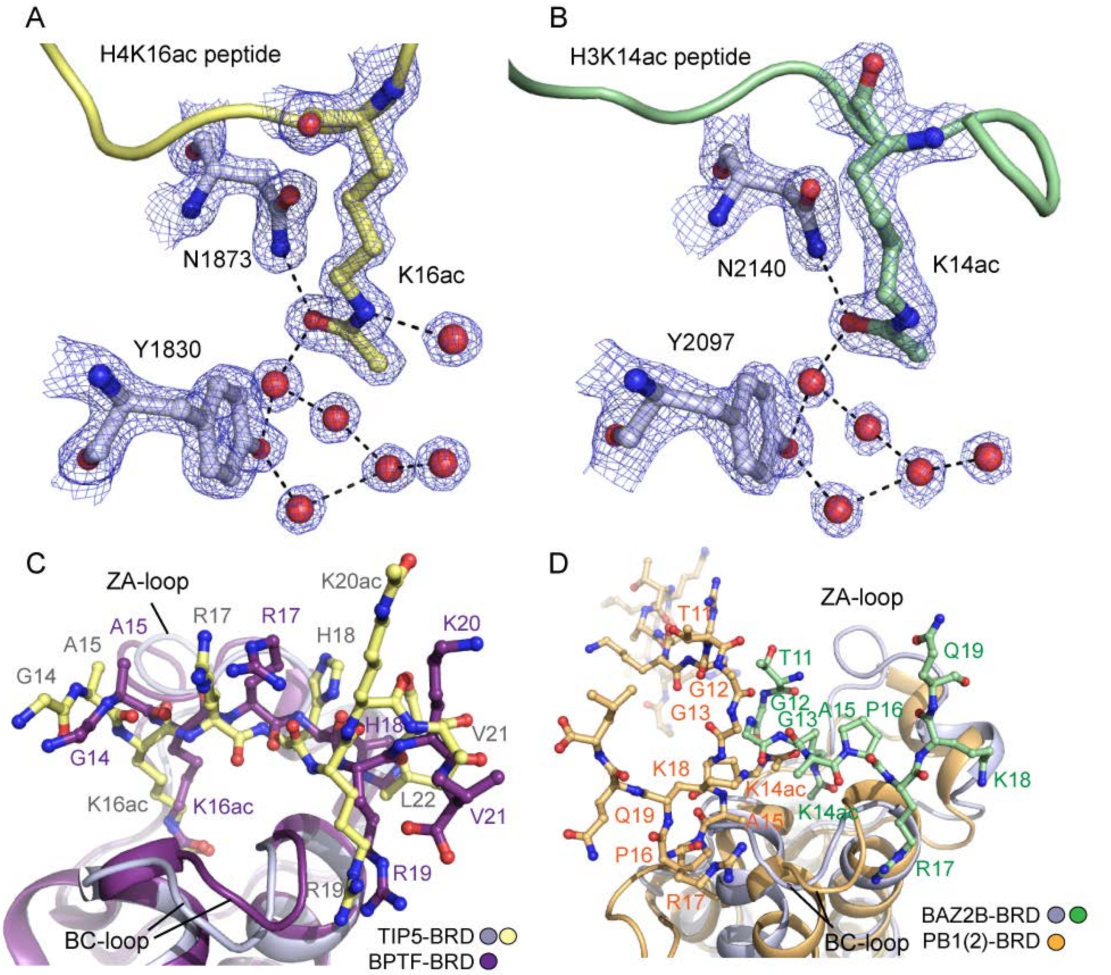

Figure S5 (related to Figure 3 and Table 2). A, B. Zoom in of the N-acetyl binding site displaying 2Fo-Fc electron density contoured at 1.8 σ. The acetyl group is surrounded by a ring of water molecules (red spheres) in both complex structures of TIP5 + H4K16ac (A) and BAZ2B + H3K14ac (B). **C.** Superposition of co-crystal structures of TIP5 and BPTF (PDB: 3QZS) on bromodomain Cα positions. **D.** Superposition of co-crystal structure of BAZ2B and solution structure of PB1 domain 2 (PDB: 2KTB) on bromodomain Cα positions.

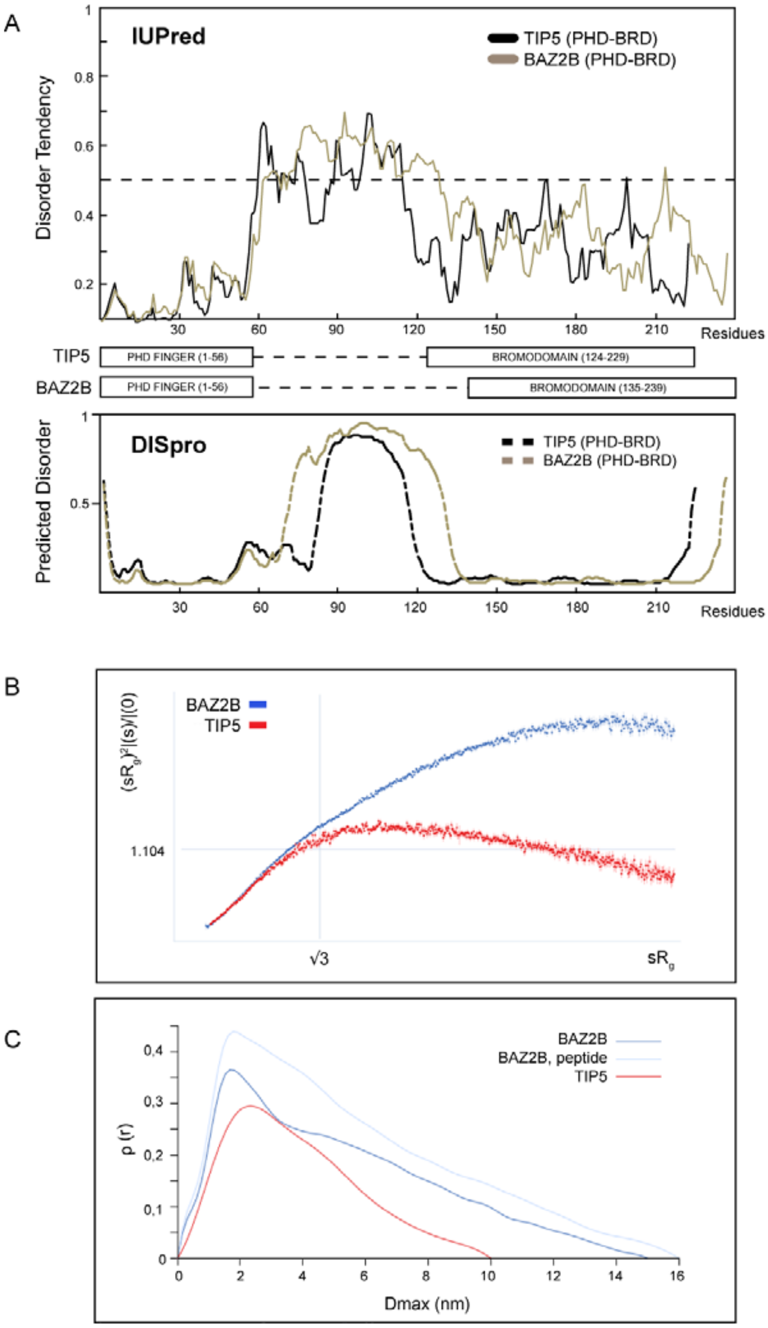

Figure S6 (related to Figure 5). Predicted disordered regions of the C-terminus part of human TIP5 and BAZ2B proteins (PHD-Bromodomain). Top panel: disorder output from IUPred software, which is able to predict intrinsically unstructured regions based on estimated energy content from amino acid composition. Bottom panel: *ab initio* prediction of disordered regions with DISpro, which is based on evolutionary information, predicted secondary structure and relative solvent accessibility. **B.** Kratky plots of the experimental data for TIP5 (red curve) and BAZ2B (blue curve) tandem domain in the free state. **C.** Distance distribution function of TIP5 and BAZ2B tandem domains in the free form (red and dark blue, respectively) and BAZ2B in complex with the peptide (light blue).

Table S1 (related to Figure 5). Statistics of the SAXS data collected at X33-DORIS and P12-PETRA III beamlines for BAZ2B and TIP5, respectively. Included are the data collection parameters, structural parameters, molecular mass determination and the software employed for the data processing and model determination.

Table S1: SAXS Data-collection and scattering-derived parameters

**Data collection parameters**	Apo TIP5 (PHD-BRD)	Apo BAZ2B (PHD-BRD)	Complex BAZ2B (PHD-BRD) with H3K14ac
Instrument	P12 (PETRA III)	X33 (DORIS)	X33 (DORIS)
Beam geometry (mm^2)	0.2 x 0.12	2 × 0.6	2 × 0.6
Wavelenght (Å)	1.24	1.5	1.5
q range ($Å^{-1}$)	0.007 – 0.465	0.009 - 0.601	0.009 - 0.601
Concentration range (mg ml^{-1})	0.7 - 9.6	1.2 - 10.7	2 - 6
Temperature (K)	283	283	283
**Structural parameters**			
I (0) (A.U.) [from P(r)]	1800 ± 100	28.5 ± 0.5	37.5 ± 0.5
R_g (Å) [from P(r)]	31 ± 1	42 ± 1	45 ± 1
I (0) (A.U.) [from Guinier]	1800 ± 100	28.0 ± 0.5	37.5 ± 0.5
R_g (Å) [from Guinier]	30 ± 1	40 ± 1	42 ± 1
D_{max} (Å)	100 ± 10	145 ± 10	160 ± 10
Porod volume estimate ($Å^3$)	45000 ± 2000	43000 ± 2000	52000 ± 2000
**Molecular-mass determination**			
Partial specific volume (cm^3 g^{-1})	0.724	0.724	0.724
Contrast ($\Delta\rho \times 10^{10}$ cm^{-2})	3.047	3.047	3.047
Molecular mass M_r [from *I* (0)]	28000 ± 5000	33000 ± 5000	45000 ± 5000
Molecular mass M_r [from Porod volume]	26000 ± 5000	25000 ± 5000	31000 ± 5000
Calculated monomeric M_r from sequence	26718	27678	32400
**Software employed**			
Primary data reduction	PRIMUS	PRIMUS	PRIMUS
Data processing	AUTOGNOM	AUTOGNOM	AUTOGNOM
Ab initio analysis	DAMMIF	DAMMIF	DAMMIF
Validation and averaging	DAMAVER	DAMAVER	DAMAVER
Rigid-body modeling	BUNCH	EOM	N/A
Computation of model intensities	CRYSOL	N/A	N/A
Three-dimensional graphic representations	PyMOL	PyMOL	PyMOL

EXTENDED EXPERIMENTAL PROCEDURES

Constructs

The cDNAs encoding tandem PHD zinc finger and bromodomain of human TIP5 (residues 1673–1901, UniProt: Q9UIF9) and human BAZ2B (residues 1928-2168, GeneBank: Q9UIF8) were synthesized by GenScript with codons optimized for *E. coli.* expression and were subcloned into a modified pET15 vector using NdeI/XhoI sites for expression as recombinant proteins with a His6 tag fused with small ubiquitin like modifier (SUMO)-1 fusion tag at the N terminus. cDNA sequences were used as a template to amplify single PHD zinc fingers (residues 1673-1728 for TIP5; residues 1928-1983 for BAZ2B) and bromodomains (residues 1797-1899 for TIP5, residues 2054-2168 for BAZ2B) regions. Single PHD zinc fingers were introduced into the same modified SUMO tagged plasmid whereas single bromodomains were further subcloned into pET28 derived expression vectors, pNIC28-Bsa4 using ligation independent cloning (Stols et al., 2002).

Protein Expression and Purification

Best expression constructs encoding all target proteins were transformed into *E. coli* competent BL21(DE3) Rosetta cells (Invitrogen) or into BL21 (DE3)-R3-pRARE2 cells (phage-resistant derivative with a pRARE plasmid encoding rare codon tRNAs). Cells were grown at 37 ºC in Terrific Broth (Sigma) from overnight cultures until A600 reached between 0.6-0.8, then the media was cooled and 0.2 mM isopropyl-β-Dthiogalactopyranoside (IPTG) was added to induce the protein expression at 20 ºC for 16 hours. An extra 0.1 mM $ZnCl_2$ was added in the protein expression to those targets that contained a PHD zinc finger.

Different purification procedures have been optimized for each target protein. In common, *E. coli* overexpressed cells were lysed using an EmulsiFlex-C5 high-pressure homogenizer (Avestin - Mannheim, Germany) or high-pressure cell disruption (Constant Systems Limited) in loading buffer A from the nickel affinity column (HisTrap Chelating FF 5ml) in the presence of Protease Inhibitor Cocktail EDTA-free (Roche). Lysates were cleared by centrifugation (14,000 x g for 45min at 4 ºC, JLA 16.250 rotor, on a Beckman Coulter Avanti J-20 XP centrifuge). After centrifugation, the supernatant was loaded onto the nickel column and eluted in an imidazole linear gradient. The

eluted proteins were collected and treated overnight with SENP1 (SUMO endoprotease-1) or TEV (Tobacco Etch Virus) proteases at 4 ºC to remove their respective N terminal tags. Digested proteins were loaded onto a nickel column again to remove the cleaved 6His-SUMO tag and the hexa-histidine expression tag proteases used. The flow through containing the untagged proteins were collected and further dialysed and purified by an ion exchange chromatography (RESOURCE Q 6 ml or RESOURCE S 6 ml GE Healthcare Life Sciences), depending on the target protein). Proteins were eluted in a linear gradient of NaCl. Finally, a polishing step was performed through a size exclusion chromatography (HiLoad 16/600 Superdex 75 or 200 GE Healthcare Life Sciences). The major peak was pooled and concentrated to high concentration in order to set up crystallization trials. ESI-MS (electrospray ionization mass spectrometry) analysis of the purified samples was performed under native conditions to elucidate the oligomerization state of the tandem domain and the single PHD zinc finger domain (both length construct). For all cases, spectra showed unambiguously monomer species corresponding to their molecular weights. TIP5 and BAZ2B tandem protein have molecular weights of 26828.44 ± 17.13 Da and 27788.94 ± 14.66 Da.

Crystallization

Crystallization of PHD fingers of TIP5 and BAZ2B in the free-form: Crystals of the free-form of single TIP5 and BAZ2B PHD Zinc fingers were grown by mixing equal volumes of 6 and 5 mg/ml protein, respectively, and crystallization buffer (2.2-1.8 M Na/K phosphate pH 8) at 20 ºC. In follow-up screens, we obtained crystals appearing after two days and reaching their full lengths within a week. In both cases, the protein buffer was 20 mM Tris·HCl pH 8, 200 mM NaCl, 2 mM DTT, 20 µM $ZnCl_2$. The crystals were flash-frozen at 100 K in a nitrogen gas stream in the cryoprotectant with 20 % glycerol.

Histone peptide H3K4 complex crystallization with TIP5 PHD finger: peptide was soaked into pre-formed apo TIP5 PHD finger crystals grown in 2 M malic acid pH 7 as crystallization condition. Soaking solution contained 1.6 M malic acid and 12 mM H3K4 5-mer peptide and the crystals were soaked for > 12h at 20ºC. Crystals were cryo-protected in 1.4 M malic acid, 6 mM H3K4 and 20 % ethyleneglycol.

Crystallization of Bromodomain of TIP5 in the free-form: clusters of thin needle-shaped crystals of the free-form of single TIP5 bromodomain were obtained by mixing equal volumes of 23 mg/ml and crystallization buffer (0.1 M Tris·HCl pH 8.4, 0.25 M $MgCl_2$, 22 % PEG 3350) at 4ºC. Several rounds of macro-seeding were needed to obtain

thicker and isolated bar-shaped crystals using the sitting drop vapour diffusion method. The protein buffer was 20 mM Tris·HCl pH 7.5, 200 mM NaCl, 2 mM DTT. A single crystal was flash-frozen at 100 K in a nitrogen gas stream in cryoprotectant solution containing 15 % ethylene glycol.

Histone peptide H4K16acK20ac complex crystallization with TIP5 bromodomain: protein was concentrated up to 17 mg/ml and co-crystallised with 1:5 M excess of the 9-mer peptide in 150 nl + 150 nl + 20 nl seeds (from 2nd round) sitting drop at 4 ºC. Prism-like crystals appeared in 2-3 days and the seeding helped to prevent twinning issues. The optimised crystallisation conditions were 0.1 M potassium phosphate monobasic, 0.1M MES pH 6.5, 2 M sodium chloride. 20 % glycerol was used as the cryoprotectant.

Histone peptides H3K14ac and H4K8acK12ac complex crystallization with BAZ2B bromodomain: Protein was concentrated up to 23 mg/ml and co-crystallised with 1:5 M excess of each peptide in 150 nl + 150 nl sitting drop at 4 ºC. The optimised crystallisation conditions were 0.1 M MES pH 5.9, 0.01m zinc sulphate, 25 % PEG methyl ether 550 for H3K14ac complex crystals; 0.1M cacodylate pH 6.5, 0.2 M calcium acetate, 18 % PEG8000 for H4K8acK12ac complex crystals; 20 % glycerol was used as the cryoprotectant.

SUPPLEMENTAL REFERENCES

Chakravarty, S., Zeng, L., and Zhou, M.M. (2009) Structure and site-specific recognition of histone H3 by the PHD finger of human autoimmune regulator. Structure *17*, 670-679.

Fiedler, M., Sanchez-Barrena, M.J., Nekrasov, M., Mieszczanek, J., Rybin, V., Muller, J., Evans, P., and Bienz, M. (2008). Decoding of methylated histone H3 tail by the Pygo-BCL9 Wnt signaling complex. Mol Cell *30*, 507-518.

Lan, F., Collins, R.E., De Cegli, R., Alpatov, R., Horton, J.R., Shi, X., Gozani, O., Cheng, X., and Shi, Y. (2007). Recognition of unmethylated histone H3 lysine 4 links BHC80 to LSD1-mediated gene repression. Nature *448*, 718-722.

Li, H., Ilin, S., Wang, W., Duncan, E.M., Wysocka, J., Allis, C.D., and Patel, D.J. (2006). Molecular basis for site-specific read-out of histone H3K4me3 by the BPTF PHD finger of NURF. Nature *442*, 91-95.

Mansfield, R.E., Musselman, C.A., Kwan, A.H., Oliver, S.S., Garske, A.L., Davrazou, F., Denu, J.M., Kutateladze, T.G., and Mackay, J.P. (2011) Plant homeodomain (PHD) fingers of CHD4 are histone H3-binding modules with preference for unmodified H3K4 and methylated H3K9. J Biol Chem *286*, 11779-11791.

Ooi, S.K., Qiu, C., Bernstein, E., Li, K., Jia, D., Yang, Z., Erdjument-Bromage, H., Tempst, P., Lin, S.P., Allis, C.D*., et al.* (2007). DNMT3L connects unmethylated lysine 4 of histone H3 to de novo methylation of DNA. Nature *448*, 714-717.

Palacios, A., Munoz, I.G., Pantoja-Uceda, D., Marcaida, M.J., Torres, D., Martin-Garcia, J.M., Luque, I., Montoya, G., and Blanco, F.J. (2008). Molecular basis of histone H3K4me3 recognition by ING4. J Biol Chem *283*, 15956-15964.

Pascual, J., Martinez-Yamout, M., Dyson, H.J., and Wright, P.E. (2000). Structure of the PHD zinc finger from human Williams-Beuren syndrome transcription factor. J Mol Biol *304*, 723-729.

Pena, P.V., Hom, R.A., Hung, T., Lin, H., Kuo, A.J., Wong, R.P., Subach, O.M., Champagne, K.S., Zhao, R., Verkhusha, V.V.*, et al.* (2008). Histone H3K4me3 binding is required for the DNA repair and apoptotic activities of ING1 tumor suppressor. J Mol Biol *380*, 303-312.

Petoukhov, MV., Franke, D., Shkumatov, AV., Tria, G., Kikhney AG., Gajda, M., Gorba, C., Mertens HDT., Konarev, PV., and Svergun DI. (2012) New developments in the *ATSAS* program package for small-angle scattering data analysis. J. Appl. Cryst. 45, 342-350.

Ramon-Maiques, S., Kuo, A.J., Carney, D., Matthews, A.G., Oettinger, M.A., Gozani, O., and Yang, W. (2007). The plant homeodomain finger of RAG2 recognizes histone H3 methylated at both lysine-4 and arginine-2. Proc Natl Acad Sci U S A *104*, 18993-18998.

Stols, L., Gu, M.Y., Dieckman, L., Raffen, R., Collart, F.R., and Donnelly, M.I. (2002). A new vector for high-throughput, ligation-independent cloning encoding a tobacco etch virus protease cleavage site. Protein Expr. Purif. 25, 8–15.

Wang, Z., Song, J., Milne, T.A., Wang, G.G., Li, H., Allis, C.D., and Patel, D.J. (2010) Pro isomerization in MLL1 PHD3-bromo cassette connects H3K4me readout to CyP33 and HDAC-mediated repression. Cell *141*, 1183-1194.

Wen, H., Li, J., Song, T., Lu, M., Kan, P.Y., Lee, M.G., Sha, B., and Shi, X. (2010) Recognition of histone H3K4 trimethylation by the plant homeodomain of PHF2 modulates histone demethylation. J Biol Chem *285*, 9322-9326.

Zeng, L., Zhang, Q., Li, S., Plotnikov, A.N., Walsh, M.J., and Zhou, M.M. (2010) Mechanism and regulation of acetylated histone binding by the tandem PHD finger of DPF3b. Nature *466*, 258-262.
